# Cyclohexanohemicucurbit[8]uril Inclusion Complexes With Heterocycles and Selective Extraction of Sulfur Compounds From Water

**DOI:** 10.3389/fchem.2021.786746

**Published:** 2021-12-03

**Authors:** Tatsiana Shalima, Kamini A. Mishra, Sandra Kaabel, Lukas Ustrnul, Simona Bartkova, Kaia Tõnsuaadu, Ivo Heinmaa, Riina Aav

**Affiliations:** ^1^ Department of Chemistry and Biotechnology, School of Science, Tallinn University of Technology, Tallinn, Estonia; ^2^ Department of Chemistry, McGill University, Montreal, QC, Canada; ^3^ Laboratory of Inorganic Materials, School of Engineering, Institute of Materials and Environmental Technology, Tallinn University of Technology, Tallinn, Estonia; ^4^ Laboratory of Chemical Physics, National Institute of Chemical Physics and Biophysics, Tallinn, Estonia

**Keywords:** Hemicucurbituril, solid-phase extraction, heterocycles, inclusion complex, lipoic acid, sorbent recycling, SC-XRD, MAS NMR

## Abstract

Solid-phase extraction that utilizes selective macrocyclic receptors can serve as a useful tool for removal of chemical wastes. Hemicucurbiturils are known to form inclusion complexes with suitably sized anions; however, their use in selective binding of non-charged species is still very limited. In this study, we found that cyclohexanohemicucurbit[8]uril encapsulates five- and six-membered sulfur- and oxygen-containing unsubstituted heterocycles, which is investigated by single-crystal X-ray diffraction, NMR spectroscopy, isothermal titration calorimetry, and thermogravimetry. The macrocycle acts as a promising selective sorption material for the extraction of sulfur heterocycles, such as 1,3-dithiolane and *α*-lipoic acid, from water.

## Introduction

**FIGURE 1:**
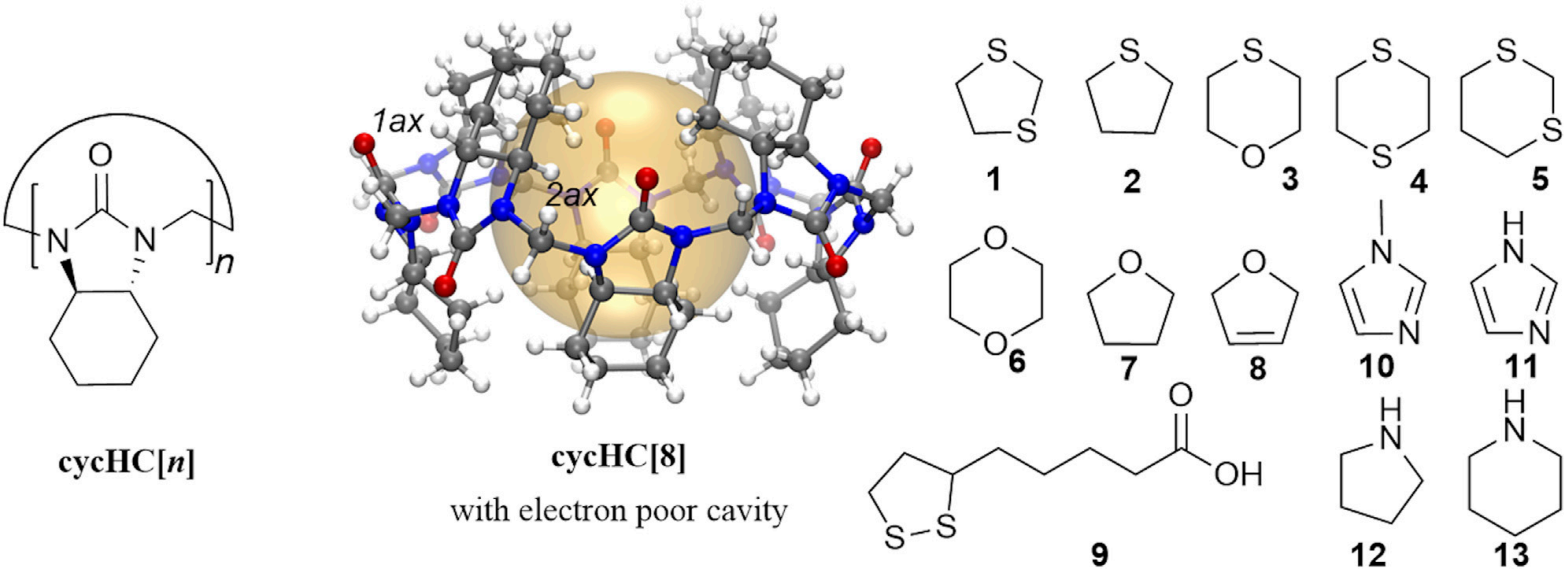
Structure of cycHC[*n*] and cycHC[8], highlighting the electron-poor cavity for guest binding and characteristic atom numbers, and the scope of studied guests.

Hemicucurbiturils, formed in templated single-step oligomerization reactions (Kaabel and Aav, 2018), are single-bridged cucurbituril-type macrocycles (Andersen et al., 2018; Lizal and Sindelar, 2018; Xi et al., 2018) that bear an electron-deficient hydrophobic cavity. The latter grants these macrocycles the ability to encapsulate anions (Buschmann et al., 2005; Cucolea et al., 2016; Kaabel et al., 2017; Assaf and Nau, 2018; Reany et al., 2018; Vázquez and Sindelar, 2018; Andersen et al., 2019; Kandrnálová et al., 2019; Valkenier et al., 2019; Maršálek and Šindelář, 2020); in addition, the formation of complexes with acids and some neutral species has been reported in the previous work. In particular, unsubstituted hemicucurbit[*n*]urils (*n* = 6, 12) bind phenol derivatives (Jin et al., 2016a; 2016b) and ferrocene (Jin et al., 2017), and cyclohexanohemicucurbit[*n*]urils cycHC [*n*] (*n* = 6, 8, 12) (Li et al., 2009; Aav et al., 2013; Prigorchenko et al., 2015, 2019; Mishra et al., 2020) form external complexes with both inorganic and organic acids (Öeren et al., 2014; Ustrnul et al., 2019, 2021). We envisioned that heterocycles **1–13** have relatively high electron densities compared to carbocycles and may therefore be able to occupy space within the eight-membered cycHC[8] (Figure 1).

S-heterocycles are compounds of interest as these substances are bioactive (Rezanka et al., 2006; Lamberth et al., 2015) and may contribute to the distinct aromas of food (Mottram and Mottram, 2002; Mahadevan and Farmer, 2006; Schoenauer and Schieberle, 2018), and heterocycle **2** is added as an odorant to the natural gas. Unsubstituted S-containing heterocycles, such as 1,3-dithiolane **1** and 1,4-thioxane **3**, are found in meat (Garbusov et al., 1976) and around chemical warfare dumping sites where they are formed due to the degradation of mustard gas (Røen et al., 2010; Magnusson et al., 2016; Vanninen et al., 2020). Bioactive *α*-lipoic acid **9** exhibits antioxidant properties (Rochette et al., 2013; Salehi et al., 2019), and to date, cyclodextrins have been used to enhance its solubility and bioavailability (Lin-Hui et al., 1995; Maeda et al., 2010; Takahashi et al., 2011; Ikuta et al., 2013; Racz et al., 2013; Caira et al., 2017; Celebioglu and Uyar, 2019). Carbon-based materials are used for solid-phase extraction (SPE) of S-heterocycles from water (Lees et al., 2017; Jõul et al., 2018); however, such systems are designed to non-selectively retain all non-polar to moderately polar components.

In this study, we report that cycHC[8] encapsulates neutral electron-rich heterocycles containing sulfur and oxygen atoms and acts as a selective sorbent material for suitably sized S-heterocycles. Complexation was characterized by ^1^H NMR titration and isothermal titration calorimetry (ITC), single-crystal X-ray diffraction (SC-XRD), ^13^C solid-state CPMAS NMR (ssNMR), and thermogravimetric analysis (TGA) and applied in SPE.

## Materials and Methods

### Materials, Reagents, and Solvents

All reagents and solvents were purchased from commercial suppliers. Ultrapure water for sample preparation in ITC and extraction studies was obtained by means of Milli-Q^®^ IQ 7003/05/10/15. Macrocyclic host compounds (cycHC[*n*]) were synthesized in our laboratory according to the procedures described in the literature (Aav et al., 2013; Prigorchenko et al., 2015; Kaabel et al., 2019).

### Binding of Heterocycles in Solid State

Single crystals of the host–guest complexes were obtained from saturated cycHC[8] solutions in methanol by adding 20 μL of the respective guest compound. SC-XRD data were collected at 123 K on a Rigaku Compact HomeLab diffractometer, equipped with a Saturn 944 HG CCD detector and an Oxford Cryostream cooling system using monochromatic Cu-*Kα* radiation (1.54178Å) from a MicroMax™-003 sealed tube microfocus X-ray source. The crystallographic data are deposited with the Cambridge Crystallographic Data Centre (CCDC 2069875–2069879) and can be obtained free of charge *via* www.ccdc.cam.ac.uk/data_request/cif.

Complexation between 1,3-dithiolane and cycHC[*n*] upon SPE was investigated by simultaneous thermogravimetry and differential thermal analysis coupled with evolved gas mass spectrometric analysis (TG-DTA/EGA-MS). The measurements were performed in the apparatus consisting of a Setaram SetSys-Evo 1600 thermal analyzer and a Pfeiffer OmniStar quadrupole mass spectrometer. Additionally, binding of 1,3-dithiolane and *α*-lipoic acid by cycHC[*n*] was characterized with ^13^C ssNMR spectroscopy. The solid complexes were obtained *via* ball milling of cycHC[8] with the respective guest in the presence of a small amount of water. The ssNMR spectra were acquired on a Bruker Avance II spectrometer at 14.1 T magnetic field (^13^C resonance frequency 150.91 MHz) using a home-built MAS probe for 25 × 4-mm Si_3_N_4_ rotors.

### Binding of Heterocycles in Solution

Complexation-induced shifts of cycHC[8] were studied by ^1^H NMR spectroscopy in 3 mM CD_3_OD solution upon addition of 60 eq. of the respective guest compound. Association constants for the complexation with 1,3-dithiolane, 1,4-thioxane, and 1,4-dioxane were determined by ^1^H NMR titration. ^1^H NMR (400 MHz) spectra in solution were recorded on a Bruker Avance III spectrometer. Thermodynamic measurements by ITC were performed on a MicroCal PEAQ-ITC calorimeter using a 200-μL calorimetric cell and a 40-μL syringe.

### Characterization of the Sorbents

Prior to analysis and further extraction experiments, cycHC[*n*] were milled in an FTS-1000 shaker mill at 30 Hz frequency by using a 14-ml ZrO_2_-coated grinding jar charged with 3 × 7-mm ZrO_2_ milling balls for 30 min. Surface area analysis of the milled cycHC[*n*] was performed on a KELVIN 1040/1042 Sorptometer at 150°C with N_2_ as an adsorptive gas and He as a carrier gas. The obtained data were processed by Kelvin 1042 V3.12 software. Microscopic investigation of cycHC[*n*] particle sizes and their distribution was carried out before and after milling. Solid samples were examined by using an Olympus BX61 microscope. The acquired images were further analyzed by CellProfiler (version 4.0.3) software (Carpenter et al., 2006; McQuin et al., 2018).

### Extraction of Heterocycles From Aqueous Solutions

SPE was performed for 0.4–2.7 mM aqueous solutions of heterocyclic guests. A solid sorbent [5 or 20 M excess of cycHC[*n*] or powdered silicarbon TH90 special, Aktivkohle, taken in the equivalent amount by weight/extraction performance to that of the macrocyclic host] was dispersed in the guest solution and rotated for 30–60 min on a Vortex-Genie 2 mixer or Stuart magnetic stirrer. The heterogeneous mixture was further separated by using either a Hettich Universal 32R centrifuge or RC membrane syringe filters, and the liquid phase was analyzed for the guest content by HPLC or UV spectrophotometry. HPLC determination was performed on an Agilent 1200 Series HPLC system equipped with a multiple wavelength detector (MWD), Macherey-Nagel Nucleoshell RP18 column (150 × 3.0 mm, 2.7 µm), or Phenomenex Kinetex XB-C18 column (150 × 4.6 mm, 2.6 µm). UV absorption was measured by using a Jasco V-730 dual beam spectrophotometer and Varian Cary 50 UV-vis spectrophotometer in 10 mm quartz cuvettes. Mettler Toledo AB204-2 analytical balances (precision 0.1 mg) and Radwag MYA 11.4Y microbalances (precision 0.006 mg) were used in sample preparation.

Reusability of cycHC[8] was investigated by comparing its removal efficiency after four sorption–desorption cycles. The sorption step was performed analogously to the extraction procedure. The desorption step included rinsing the material with water, drying for 6 h at 110–120°C in the oven, followed by additional drying for 3 h under vacuum. The dried macrocycle was milled according to the general procedure and utilized in the subsequent cycle.

## Results and Discussion

### SC-XRD of Inclusion Complexes

**FIGURE 2:**
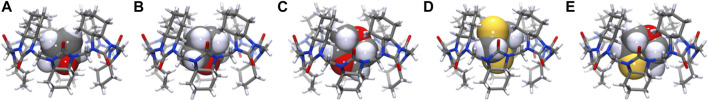
Crystal structures of cycHC[8] inclusion complexes with neutral heterocycles with increasing packing coefficient order: **8**@cycHC[8] **(A)** (PC 0.51), **7**@cycHC[8]; **(B)** (PC 0.54), **6**@cycHC[8]; **(C)**(PC 0.61), **1**@cycHC[8]; **(D)** (PC 0.66), **3**@cycHC[8]; **(E)** (PC 0.69). Their packing coefficient values are the ratios between *V*_*guest*_ to *V*_*cavity*_ (host), reflecting the space filled by the encapsulated guest in the host cavity (Mecozzi and Jr, 1998). For more details, see Supplementary Material S13.

A series of compatibly sized electron-rich S-, O-, and N-heterocyclic compounds were crystallized *via* slow evaporation, and compounds **1, 3, 6, 7**, and **8** formed inclusion complexes with cycHC[8] upon co-crystallization (Figure 2 and Supplementary Materials S4–S13). The N-containing heterocycles, **12** and **13**, and the largest explored guest, **4**, did not yield crystals of inclusion complexes with cycHC[8]. Packing of **1** and **3** inclusion complexes with cycHC[8] gave rise to isomorphous (Z’ = 4) crystal structures (Supplementary Materials S10,11), in an arrangement previously unrecorded for cycHC[8] inclusion complexes. The packing of complexes involving O-containing smaller heterocycles **6**, **7**, and **8** appears to be mainly directed by hydrogen bonding interactions with methanol such that the resulting crystal structures are isomorphous to each other (Supplementary Materials S6–S8) and the previously published methanol solvate of cycHC[8] (Prigorchenko et al., 2015). The smaller guest molecules **6**, **7**, and **8** had a total site occupancy limited to 50–75% of the resolved disorder components. The remaining electron density map exhibited no clear features, making it impossible to resolve whether the diffuse component of the guest disorder also includes partial substitutional disorder from methanol. In contrast, the position of the larger S-containing guests, **1** and **3**, is more conserved within the respective crystal structures (Supplementary Materials S12,13) with almost no diffuse component observed, indicating that these guests have fewer orientations available within cycHC[8]. Notably, analyzing the disorder models of all inclusion complexes reveals a similarity throughout, namely, where guests are oriented with heteroatoms close to the portals of cycHC[8] (Figure 2 and Supplementary Materials S6–S13). In several structures, heterocycles are located at a suitable distance (2.7–2.8 Å) from a methanol molecule at the portal of cycHC[8] and can therefore potentially accept hydrogen bonds *via* the portals of the macrocycle. This process would explain the observed conservation of this type of guest orientation motif. The guest molecules are tightly enwrapped within cycHC[8], especially the largest S-containing compounds, **1** and **3**, that fill close to 70% of the cavity volume (Figure 2, complexes D and E), indicating that guest binding and release must be accompanied by opening and closing of the host portals. Similar conformational dynamics of cycHC[8] have been previously observed and computationally described in the binding of large anionic guests (Kaabel et al., 2017).

### ^1^H NMR and ITC Binding Studies in Solution

**FIGURE 3:**
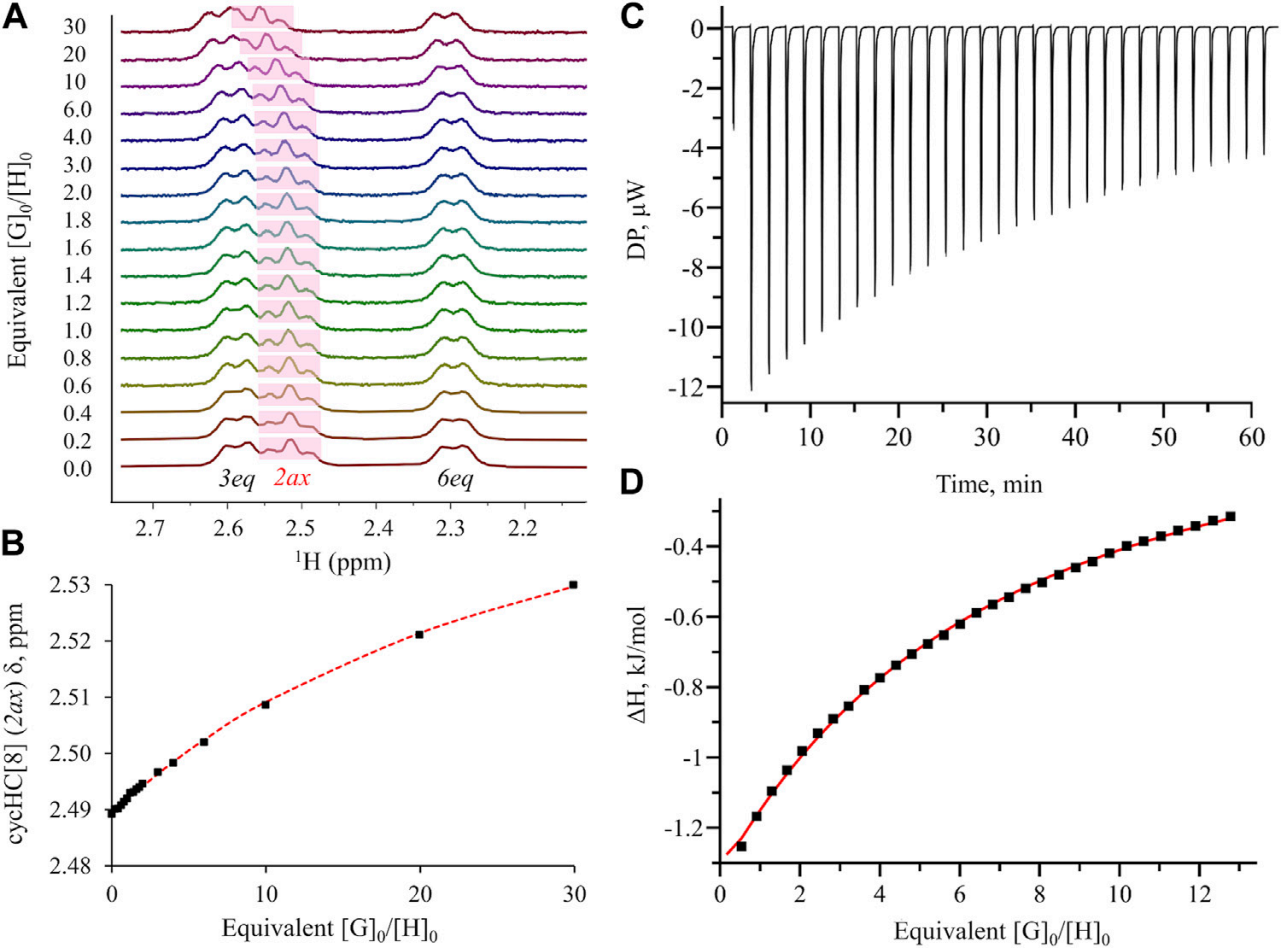
^1^H NMR and ITC titration for cycHC[8] binding with **1** **(A)** spectra for NMR titration in CD_3_OD; **(B)** binding isotherm for NMR titration assuming the 1:1 binding model; **(C)** raw thermogram for ITC measurement in the CH_3_OH:H_2_O (50:50) mixture; **(D)** binding isotherm for ITC measurement using the “one set of sites” model. More details are provided in Supplementary Material S16–S31.

Furthermore, we evaluated host-guest complex formation in CD3OD solution. Inclusion complex formation was followed by an observed chemical shift change of cycHC[8] proton H2ax (Figure 1 and Supplementary Materials S14, S15) positioned inside the cavity. Our screening study revealed that S-containing five-membered heterocycles **1** and **2** caused larger chemical shift changes of 0.064 and 0.048 ppm, respectively, compared to the six-membered heterocycles **3**, **4**, **5**, and **6**. A negligible shift was observed for **7**, while no shift was observed for **8** or the N_heterocycles **10** and **11**. Signals of **9** overlapped with the characteristic cycHC[8] signal, and, therefore, its encapsulation could not be evaluated by ^1^H NMR. Nevertheless, all chemical shift changes were relatively small compared to those that occurred upon binding of anions (Kaabel et al., 2017). The binding of **1**, **3**, and **6** was further evaluated by ^1^H NMR titration (Figures 3A,B). The guest binding in methanol followed the order of logP values (Supplementary Materials S48) and agreed with our screening study; the strongest binding was shown for **1**, followed by **3** and **6**, with values of K = 7.9 ± 0.2 M^−1^, 2.18 ± 0.04 M^−1^, and 1.77 ± 0.04 M^−1^, respectively (Supplementary Materials S16–S22) for the 1:1 binding model.

The thermodynamic characteristics of binding were collected by ITC (Figures 3C,D; Table 1 and Supplementary Materials S23–S31). Binding of **6** with cycHC[8] in methanol was too weak to be determined by ITC; however, *K* values for S-heterocycles **1** and **3** were in agreement with NMR data, showing the strongest binding for guest **1** (Table 1, lines 1 and 3). The binding of both guests in methanol was enthalpically favorable and entropically unfavorable. A similar binding character was observed upon the binding of chaotropic anions to cycHC[8] in protic media (Kaabel et al., 2017). Although chaotropicity is mainly attributed to ionic species, chaotrope-like organic molecules have been reported in studies of crystalline hydrates (Dobrzycki et al., 2019). The chaotropic character (Assaf and Nau, 2018) is most strongly exhibited in aqueous media, and higher solvent polarity can enhance binding to the hydrophobic host. Therefore, we further studied the binding of **1** and **3** in mixtures of CH_3_OH and H_2_O (Supplementary Materials S25–S27,S29). In the presence of water, binding of the guests was indeed stronger, increasing the association constant of **1** from 13 M^−1^ in CH_3_OH to a value of 66 M^−1^ in the 1:1 CH_3_OH:H_2_O (50:50) mixture (Table 1, lines 1–2). For the bulkier and less hydrophobic guest **3**, the observed increase in the association constant was smaller (Table 1, lines 3–4). Binding enthalpy was strongly increased in the presence of water, accompanied by a rise in the entropic penalty for both guests (Table 1). Any further increase in the proportion of water proved impossible due to the limited solubility of cycHC[8].

**TABLE 1:** Thermodynamic parameters from ITC measurements for complexation of guests **1**, **3**, and **6** with cycHC[8] for the 1:1 binding model. All energy values are given in kJ/mol.

No.	Guest	Solvent	ΔH°	–TΔS°	ΔG°	Ka, M_−1_
1	**1**	CH_3_OH	−9.8 ± 0.6	3.6	−6.2	13.1 ± 0.8
2	**1**	CH_3_OH:H_2_O (50:50)	−20.4 ± 0.9	10.2	−10.2	65.6 ± 2.5
3	**3**	CH_3_OH	−13.7 ± 1.2	11.4	−2.3	2.5 ± 0.2
4	**3**	CH_3_OH:H_2_O (50:50)	−42.9 ± 3.9	39.6	−3.3	3.7 ± 0.3

### Characterization of Solid cycHC[*n*] and SPE Experiments

The low water solubility of the hydrophobic cycHC[8] and its ability to form inclusion complexes prompted us to investigate whether cycHC[8] can be used for the sorption of heterocycles from water *via* SPE. In parallel with cycHC[8], powdered silicarbon (TH90) and cycHC[6] were used. The cycHC[6] consists of the same monomers, so the hydrophobic properties of the outer surface are very similar to those of cycHC[8], but its cavity is much smaller (35 Å^3^) (Prigorchenko et al., 2015), and thus, it cannot accommodate the heterocycles studied. Hence, cycHC[6] served as an analog for differentiation between external physisorption and inclusion complex formation during extraction. The commonly used activated carbon-based sorbent TH90 was chosen as a reference to evaluate the efficiency and selectivity of sorption. The cycHC[*n*] compounds were milled before their use in extraction; microscopy studies and image analysis of the cycHC[*n*] showed that milling led to a relatively uniform particle size of 5 μm (Supplementary Materials S32–35). The Brunauer–Emmett–Teller (BET) analysis of cycHC[*n*] by N_2_ adsorption–desorption (Supplementary Materials S36,37) found the available surface area to be relatively similar for both cycHC[6] and cycHC[8] with values of 6.03 m^2^/g and 9.02 m^2^/g, respectively; therefore, the cycHC[*n*] homologs are expected to have similar extraction efficiencies. In contrast, the surface area of commercially available TH90 is much larger (ca. 1000 m^2^/g), which allows us to predict higher extraction efficiency per weight.

**FIGURE 4:**
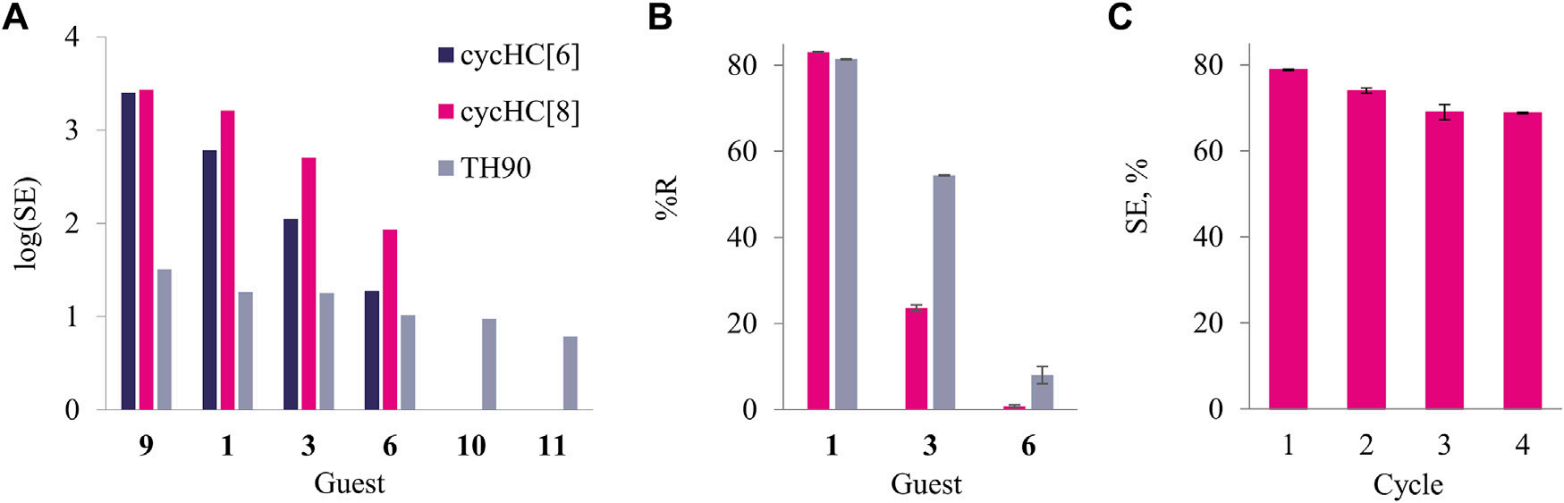
**(A)** SE of cycHC[6], cycHC[8], and TH90 (dark blue, pink, and gray, respectively) in water, displayed in logarithmic scale; **(B)** selectivity of SPE from the mixture of **1, 3**, and **6** by cycHC[8] and TH90, at comparable sorbent amount granting %R ca 80% for **1**; **(C)** %R exhibited by cycHC[8] throughout repeated extraction cycles of 1 [Error bars stand for the standard deviation between parallel experiments (n ≥ 2). For more details, see Supplementary Material S38–S50,S57].

SPE was performed for heterocycles **1, 3, 6, 9, 10**, and **11** by stirring the dispersed solid sorbent in an aqueous solution of each guest; the change in the guest concentration upon extraction was then determined (Supplementary Materials S47,48). The cycHC[*n*] demonstrated negligible removal of O-containing **6**, as well as N-containing **10** and **11**. The larger cycHC[8] proved to efficiently extract the S-containing **1** (78%) and moderately remove **3** (25%), while cycHC[6] was considerably less efficient at removing these guests, with a 16% extraction value for **1** and only 3% value for **3**. As expected, TH90 acts as a non-selective adsorbent, and taken in the same ratio of guest to sorbent (by weight), it removes over 50% of all of the studied heterocycles from water. Sorption efficiency (*SE*) was expressed as the mass of the sorbed guest (μg) per cm^2^ of the respective sorbent surface area and converted into logarithmic scale. The latter demonstrates that cycHC[*n*] possess higher affinity toward hydrophobic S-containing heterocycles, while TH90 exhibits roughly the same performance independently of the guest nature (Figure 4A). Furthermore, we evaluated sorption selectivity using a mixture of guests **1, 3**, and **6**, and the amount of sorbent (cycHC[8] and TH90) providing ca. 80% removal percentage (%R) for guest **1** (Supplementary Materials S49, S50). Both sorbents showed low sorption of the least hydrophobic guest **6**, although TH90 proved to remove ca. 10 times more than cycHC[8]. More importantly, over two times difference was observed between the affinity of two S-heterocycles **1** and **3**; cycHC[8] is 2.3 times more selective than TH90 (Figure 4B and Supplementary Material S50); in addition, cycHC[8] sorbent is reusable after binding of **1**. A simple washing and drying procedure, followed by milling, allows the reactivation of the sorbent’s surface for future use without significant loss in binding efficiency for at least four cycles (Figure 4C and Supplementary Materials S57–58).

### TGA and ^13^C ssNMR Studies

**FIGURE 5:**
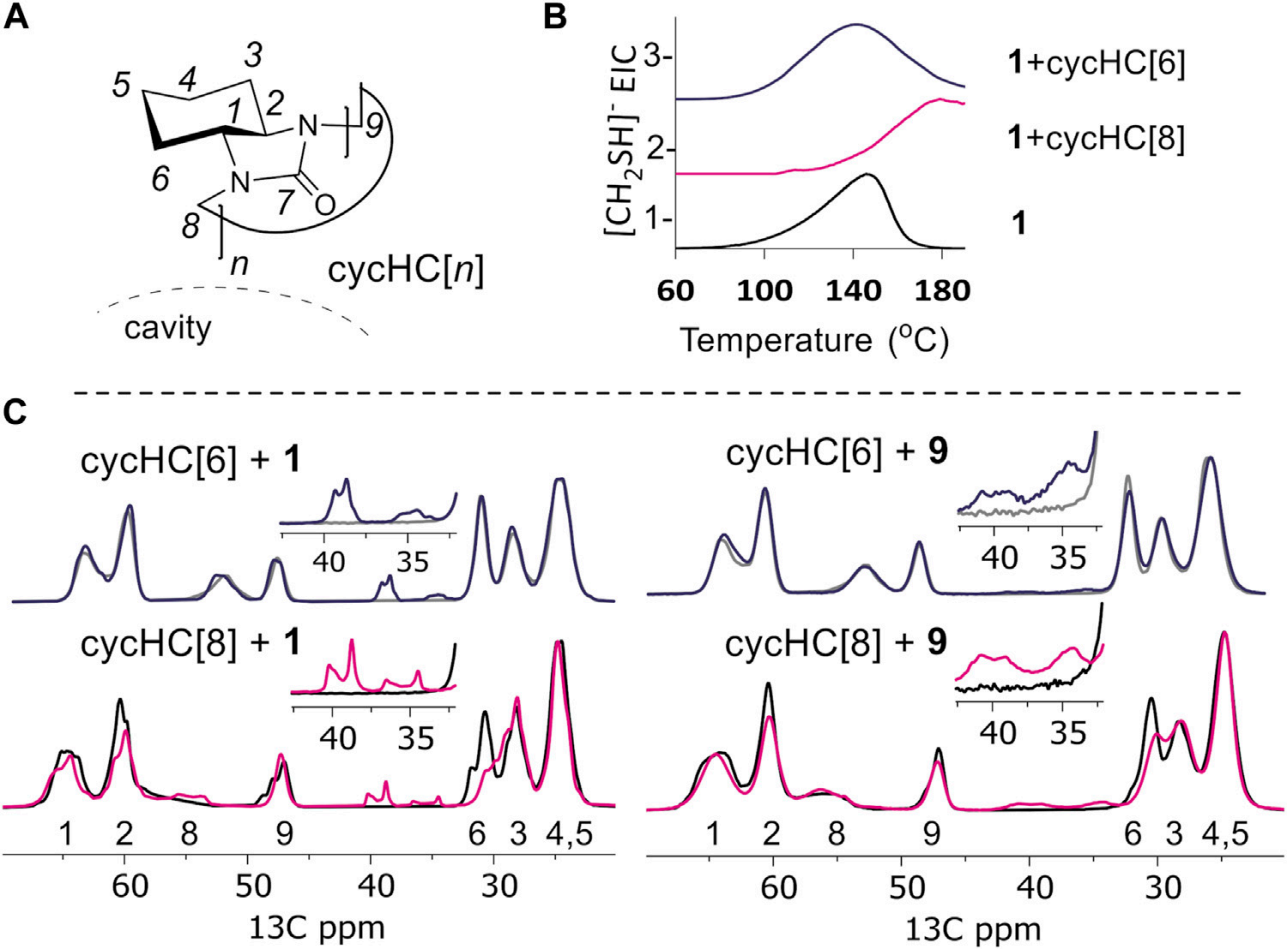
**(A)** Structure of cycHC[*n*] with atom numeration and cavity direction; **(B)** extracted ion current (EIC) of one fragment in TGA: 1 - pure **1**, 2 - cycHC[8] after extraction of **1**, 3 - cycHC[6] after extraction of **1**; **(C)** ^13^C ssNMR stacked spectra of cycHC[*n*] before and after milling with guests: upper left - **1** + cycHC[6] (blue) overlaid with cycHC[6] (gray); lower left–**1** + cycHC[8] (pink) overlaid with cycHC[8] (black); upper right - **9** + cycHC[6] (blue) overlaid with cycHC[6] (gray); lower right–**9** + cycHC[8] (pink) overlaid with cycHC[8] (black).

The formation of inclusion complexes in the cycHC[8] sorbent after SPE of **1** was confirmed by TGA, by identifying the formation of the characteristic fragmentation product [CH_2_SH]¯ (*m/z* 47) of **1** when bound to cycHC[*n*] sorbents and in pure form (see Supplementary Material S51 for details). The evolution profiles for pure **1** and its complex with cycHC[6] (Figure 5B) occur at similar temperatures of 148°C and 138°C, respectively, indicating that no inclusion complexes form with the smaller macrocycle. Furthermore, the complex with cycHC[8] releases the characteristic degradation product at a significantly higher temperature, 189°C, indicating additional interactions between cycHC[8] and one which impact higher thermal stability to **1** (Figure 5B and Supplementary Material S51). SPE of the largest and most hydrophobic guest in this study, **9**, leads to similar efficiency of extraction by cycHC[6] and cycHC[8], with *%R* values of 46 and 78%, respectively (Supplementary Materials S47,48), which may indicate a different binding behavior of **9** to cycHC[*n*] in comparison to **1**. To better understand the interaction of guests **1** and **9** with cycHC[8] and cycHC[6], we investigated complex formation using ^13^C ssNMR spectroscopy (Figure 5C and Supplementary Materials S52–56). Recently, complex formation *via* mechanochemical agitation of cucurbit[7]uril was followed by ssNMR (Dračínský et al., 2021). Powders obtained by liquid-assisted grinding of cycHC[*n*] with the guest in the presence of water served as the model for binding during the extraction process at the solute–solid interphase. Figure 5C illustrates the changes induced upon binding of **1** with cycHC[8] and cycHC[6] (Supplementary Materials S53,54). The most significant change after milling is observed in the ^13^C signal of carbons 2 and 6, pointing inside the cavity of cycHC[8] (Figure 5C lower left and Supplementary Material S54). Intensities of these signals are decreased and the chemical shift in position 6 changed from 30.6 to 29.8 ppm, evidencing the formation of inclusion complex **1**@cycHC[8]. In contrast, the spectra of **1** milled with cycHC[6] do not evidence such changes (Figure 5C upper left and Supplemetary Material S53), confirming that physisorption on the surface of cycHC[*n*] does not have a significant effect on sorbent ssNMR shifts. In an analogous experiment with **9** (Figure 5C right and Supplementary Materials S54–S56) similar trends can be observed, that is, a change in the signal intensities of carbons 2 and 6 of cycHC[8], accompanied by a slight chemical shift change at carbon 6 (Figure 5C lower right) and no change in the spectrum of cycHC[6] (Figure 5C upper right). Thus, the prominent selectivity of cycHC[8] toward binding of five-membered S-heterocycles **1** and **9** is explained by inclusion complex formation during SPE. Unfortunately, no enantioselectivity was observed during extraction of stereoisomers of **9** (Supplementary Material S48).

## Conclusion

Encapsulation of five- and six-membered electron-rich heterocyclic guests by cycHC[8] is mostly related to their size, which must be complementary with the host cavity, and the hydrophobic effect is one of the driving forces of the interaction. From the binding studies of **1** and **3** with cycHC[8] in methanol and a methanol–water mixture, complex formation was found to be even more favorable in the presence of water and the thermodynamic characteristics resembled the binding of chaotropic anions. We showed that cycHC[8] can serve as a selective solid sorbent material for SPE of sulfur heterocycles from aqueous solutions; in addition, cycHC[8] was successfully applied for selective extraction of **1** and **9**. The ssNMR, TGA, and comparative extraction by homologous cycHC[6] and commonly used TH90 revealed that prominent selectivity of cycHC[8] toward binding of five-membered S-heterocycles is driven by inclusion complex formation. Design of advanced material with increased surface area would enhance the extraction performance even further. Crucially, the solid cycHC[8] sorbent material can be reused, which makes it a candidate for applications in selective SPE systems or the removal of pollutants and other target compounds from water, based on molecular recognition properties.

## Data Availability

The datasets presented in this study can be found in Supplementary Material.
