# Supplementary material for: Cyclohexanohemicucurbit[8]uril Inclusion Complexes With Heterocycles and Selective Extraction of Sulfur Compounds From Water
Source: Front Chem. 2021 Dec 3;9:786746. doi: 10.3389/fchem.2021.786746 (PMC8678634; doi:10.3389/fchem.2021.786746)
Supplement: Supplementary file 1 [file DataSheet1.PDF]

Supplementary Material

Table of Content

General information S2
1. Binding study of S- and O-containing heterocycles with cycHC[8] S4
1.1. Formation of the inclusion complexes in the solid state S4
1.2. Complexation in solution S14
1.3. Association constant measurements S16
1.3.1. NMR titration S16
1.3.2. Isothermal calorimetric titration S23
2. Characterization of the sorbents S32
2.1. Microscopy S32
2.2. Surface area analysis S36
3. Extraction of neutral guests from water S38
3.1. Quantitative analysis of S- and O-containing heterocycles by HPLC-UV method S39
3.2. Quantitative analysis of N-containing heterocycles by UV spectrophotometry S43
3.3. Extraction results S47
3.4. Selectivity during extraction S49
4. Termogravimentric analysis of cycHCs before and after extraction of 1,3-dithiolane .. S51
5. ^{13}C solid-state NMR studies of 1,3-dithiolane and α-lipoic acid interaction with cycHCs S52
6. Regeneration of the sorbent S57
References S59

General information

Unless specified, cycHC[8] and cycHC[6] acronyms stand for (*R,R*)-cycHC[8] and (*R,R*)-cycHC[6], resepectively.

Single crystal X-ray diffraction data was collected at 123K on Rigaku Compact HomeLab diffractometer, equipped with a Saturn 944 HG CCD detector and Oxford Cryostream cooling system using monochromatic Cu-*Kα* radiation (1.54178Å) from a MicroMax^{TM}-003 sealed tube microfocus X-ray source. The strategy of data collections was calculated and implemented through the program package *HKL-3000* (Minor et al., 2006). Data was collected using *ω*-scans. CrysAlisPro (CrysAlisPro. Version 1.171.38.43, 2014) was used for data reduction and empirical absorption correction using spherical harmonics implemented in *SCALE3 ABSPACK* scaling algorithm (Clark and Reid, 1995). The structures were solved using *SHELXT* (Sheldrick, 2015b) and refined by full-matrix least-squares method against F^2 with *SHELXL-2018* (Sheldrick, 2015a) through *OLEX2* (Dolomanov et al., 2009) *p*rogram package. All non-hydrogen atoms were refined with anisotropic atomic displacement parameters. Hydrogen atoms attached to carbon atoms were treated as riding atoms, using isotropic displacement parameters U_{iso}(H) = 1.2U_{iso}(C) for CH and CH_2; U_{iso}(H) = 1.5U_{iso}(C or O) for CH_3 and OH. The absolute configuration of (*R,R*)-cycHC[8] was known based on the commercial chiral starting material (*R,R*)-1,2-diaminocyclohexane used in the synthesis (Prigorchenko et al., 2015). The figures were drawn using the programs *Mercury CSD* 3.10 (Macrae et al., 2006) and *POV-Ray* 3.7 (Persistence of Vision Raytracer. Version 3.7 (2004). Persistence of Vision Pty. Ltd.). The crystallographic data is deposited with the Cambridge Crystallographic Data Centre (CCDC **2069875–2069879**) and can be obtained free of charge via www.ccdc.cam.ac.uk/data_request/cif. The guest volumes were calculated based on the respective crystal structures, using the triangulated sphere model included in the Olex2 program package (Dolomanov et al., 2009), in which all atoms are approximated to isotropic spheres defined by the default CSD van der Waals (vdW) radii. Van der Waals radii used by the CSD, from refs (Bondi, 1964; Rowland and Taylor, 1996) are H 1.09 Å, C 1.7 Å, O 1.52 Å and S 1.8 Å. The reported guest volumes are given as an average of all symmetry independent guest molecules resolved in the asymmetric units of respective crystal structures.

^{1}H NMR (400 MHz) spectra in solution were recorded on Bruker Avance III spectrometer, using a Bruker BBO probe equipped with a z-gradient coil. Chemical shifts were referenced to residual protio solvent peak (δ ^{1}H 3.31 ppm measured in CD_3OD-*d*$_4$) as internal standard. All chemical shifts are reported in ppm units. ^{13}C solid state CPMAS NMR spectra were acquired on Bruker AVANCE-II spectrometer at 14.1 T magnetic field (^{13}C resonance frequency 150.91 MHz) using home-built MAS probe for 25 x 4 mm Si_3N_4 rotors. In all experiments the sample spinning was 12.5 kHz, sample temperature 310 K, cross polarization was performed by 2 ms ^{1}H ramped pulse, the proton decoupling was achieved by 90 kHz frequency modulated rf field. The chemical shift was referenced to TMS scale using the methyl site in glycine at 43.63 ppm.

Microscopic imaging of cycHC[*n*] samples before and after milling was performed by placing cycHC[*n*] powder on microscope slide (Kaltek, ±76 x 26 mm), and covering with coverslip (Kaltek, 18 x 18 mm) and a drop of immersion oil (Zeiss immersol 518N). Eight bright-field images for each sample type were acquired across the slide using Olympus BX61 microscope with following settings: Olympus 60X/1.42 Oil Microscope Objective PlanApo N, Mirror Cube

Free, Lamp Intensity (Transmission) 12,00 V, Exposure Time 2.222 ms, Binning 1x1, and ISO Sensitivity 200. All acquired images were saved in TIF format for further analysis.

CellProfiler (version 4.0.3) (Carpenter et al., 2006; McQuin et al., 2018) was used to construct and run an analysis pipeline for each cycHC[*n*] sample type. The pipelines were adjusted slightly to each sample type, however, the key modules and parameters remained the same and are listed here. If one is interested in each individual pipeline, we refer to the following repository on GitHub where all four pipelines are publicly available (https://github.com/taltechmicrofluidics/CP-Macrocyclic-host-compound-analysis).

The simultaneous thermogravimetric and differential thermal analysis coupled with mass spectrometric detection of evolved gas (TG–DTA/EGA–MS) was performed in an apparatus consisting of a SetSys-Evo 1600 (Setaram, France) thermal analyser and an OmniStar (Pfeiffer, Germany) quadrupole mass spectrometer (gastight high-sensitivity ion source at the voltage of 150 V). Coupling between the two equipments was provided through a heated capillary tube kept at 180 °C. The ion currents of the selected mass/charge (*m/z*) values were monitored in multiple ion detection (MID) mode (Quadera version 4.20 software) with the collection time of 100 µs for each channel. The measurements were carried out in flowing argon atmosphere in the temperature range of 30 – 250 °C using the heating rate of 10 °C/min, the gas flow rate of 60 mL/min and open Pt crucibles.

1. Binding study of S- and O-containing heterocycles with cycHC[8]

1.1. Formation of the inclusion complexes in the solid state

Table S1. Summary of crystallographic data for **guest@(*R*,*R*)-cycHC[8] · *n*CH_3OH** complexes

CCDC code	2069875	2069876	2069877	2069878	2069879
**Complex**	**tetrahydrofuran @(*R*,*R*)-cycHC[8]**	**2,5-dihydrofuran @(*R*,*R*)-cycHC[8]**	**1,4-dioxane @(*R*,*R*)-cycHC[8]**	**1,4-thioxane @(*R*,*R*)-cycHC[8]**	**1,3-dithiolane @(*R*,*R*)-cycHC[8]**
Sum. formula (asym. unit)	$C_{141}H_{234}N_{32}O_{25.25}$	$C_{141}H_{231.5}N_{32}O_{25.25}$	$C_{138.5}H_{224}N_{32}O_{24}$	$C_{269}H_{410}N_{64}O_{35.25}S_{3.25}$	$C_{272.75}H_{422.5}N_{64}O_{34.5}S_{9.5}$
Moiety formula (asym. unit)	$2(C_{64}H_{96}N_{16}O_8)$, $1.25(C_4H_8O)$, $8(CH_4O)$	$2(C_{64}H_{96}N_{16}O_8)$, $1.25(C_4H_6O)$, $8(CH_4O)$	$2(C_{64}H_{96}N_{16}O_8)$, $1.25(C_4H_8O_2)$, $5.5(CH_4O)$	$4(C_{64}H_{96}N_{16}O_8)$, $3.25(C_4H_8OS)$	$4(C_{64}H_{96}N_{16}O_8)$, $4.75(C_3H_6S_2)$, $2.5(CH_4O)$
Formula weight	2781.59	2779.07	2721.48	5208.78	5454.79
Temperature/K	123.0	123.0	123.0	123.0	123.0
Crystal system	monoclinic	monoclinic	monoclinic	monoclinic	monoclinic
Space group	$P2_1$	$P2_1$	$P2_1$	$P2_1$	$P2_1$
a/Å	16.5175(2)	16.47548(13)	16.36640(7)	22.8851(3)	22.91115(11)
b/Å	26.9534(3)	27.02477(19)	27.11642(14)	26.5891(5)	26.67867(12)
c/Å	17.9663(3)	17.94552(14)	17.92826(9)	27.1109(4)	27.04845(12)
α/°	90	90	90	90	90
β/°	96.8142(15)	96.7559(7)	96.3596(4)	89.2584(13)	90.6405(4)
γ/°	90	90	90	90	90
V/Å^3	7942.2(2)	7934.69(10)	7907.56(7)	16495.5(4)	16532.03(13)
Z	2	2	2	2	2
D_{calc}/g cm^{-3}	1.163	1.163	1.143	1.049	1.096

μ/mm^{-1}	0.656	0.656	0.643	0.756	1.132
F(000)	3012.0	3007.0	2942.0	5612.0	5870.0
Crystal size/mm^3	0.21 × 0.08 × 0.08	0.32 × 0.1 × 0.08	0.20 × 0.16 × 0.15	0.55 × 0.25 × 0.12	0.35 × 0.19 × 0.17
Radiation, λ/Å	1.54184 (CuK$_\alpha$)	1.54184 (CuK$_\alpha$)	1.54184 (CuK$_\alpha$)	1.54184 (CuK$_\alpha$)	1.54184 (CuK$_\alpha$)
2Θ range/°	4.954 to 134.874	4.958 to 134.678	4.96 to 134.7	3.862 to 134.94	3.856 to 134.802
Index ranges	$-19 \le h \le 19$, $-26 \le k \le 32$, $-21 \le l \le 19$	$-18 \le h \le 19$, $-31 \le k \le 32$, $-21 \le l \le 20$	$-19 \le h \le 19$, $-32 \le k \le 32$, $-21 \le l \le 19$	$-27 \le h \le 26$, $-27 \le k \le 31$, $-31 \le l \le 32$	$-26 \le h \le 27$, $-30 \le k \le 31$, $-31 \le l \le 31$
Reflections collected	61162	67322	58258	94485	141110
Independent reflections	23288 [R_{int} = 0.0547, R_{sigma} = 0.0557]	27570 [R_{int} = 0.0392, R_{sigma} = 0.0433]	27088 [R_{int} = 0.0286, R_{sigma} = 0.0299]	51497 [R_{int} = 0.0498, R_{sigma} = 0.0639]	54420 [R_{int} = 0.0305, R_{sigma} = 0.0339]
Data/restraints/ parameters	23288/2126/1948	27570/2128/1948	27088/1941/1868	51497/8086/4288	54420/8414/4076
Goodness-of-fit on F^2	1.026	1.041	1.052	0.990	1.049
Final R indexes [$I \ge 2\sigma(I)$]	R_1 = 0.0562, wR_2 = 0.1456	R_1 = 0.0528, wR_2 = 0.1423	R_1 = 0.0436, wR_2 = 0.1168	R_1 = 0.0673, wR_2 = 0.1775	R_1 = 0.0603, wR_2 = 0.1720
Final R indexes [all data]	R_1 = 0.0650, wR_2 = 0.1536	R_1 = 0.0598, wR_2 = 0.1504	R_1 = 0.0495, wR_2 = 0.1265	R_1 = 0.0914, wR_2 = 0.1997	R_1 = 0.0671, wR_2 = 0.1830
Largest diff. peak/hole/ eÅ^{-3}	0.59/−0.23	0.41/−0.26	0.71/−0.25	0.43/−0.46	1.02/−0.53

Crystallographic details for the tetrahydrofuran@(*R*,*R*)-cycHC[8] complex

Colourless block-shaped single crystals were obtained from a methanol solution of (*R*,*R*)- cycHC[8] in the presence of 60 molar equivalents of tetrahydrofuran (THF). The asymmetric unit of the crystal structure contains two inclusion complexes of THF@(*R*,*R*)- cycHC[8], surrounded by solvent (methanol) molecules (**Figure S1**). The encapsulated guest molecules are disordered in both complexes. One of the encapsulated THF was modelled with two disorder components, with total site occupancy reduced to *s.o.f.* 0.75. The second THF was modelled also with two disorder components, however the total site occupancy appears to be lower, and was fixed to *s.o.f.* 0.5. The relative occupancy of the disorder components was allowed to refine freely. The remaining electron density within the cycHC[8] cavities has no clear features, preventing us to fully resolve the guest disorder. Minor unresolved disorder components may include partial substitutional disorder by methanol molecules. Geometry of the resolved THF molecules was restrained using the SAME restraints in *SHELXL*. The anisotropic displacement parameters of the disorder components were restrained using SIMU restraints in *SHELXL*. Anti-bumping restraints were introduced using BUMP, to restrain the positions of minor disorder components (low *s.o.f.*) and avoid too close contacts of non-bonded atoms. The position of 8 methanol molecules could be resolved in the asymmetric unit with full occupancy. Of these, one methanol molecule was found to be disordered between two orientations, which were modelled using two disorder components with freely refined relative occupancies (*s.o.f*). The remaining solvent molecules (methanol) were too severely disordered to be adequately modelled and were therefore accounted for using the SQUEEZE algorithm of *PLATON* [9]. The electron density in the detected voids (probe radius 1.2 Å, 125 electrons in 540 Å^3 void, 7% of the unit cell volume) can include unresolved methanol and THF molecules.

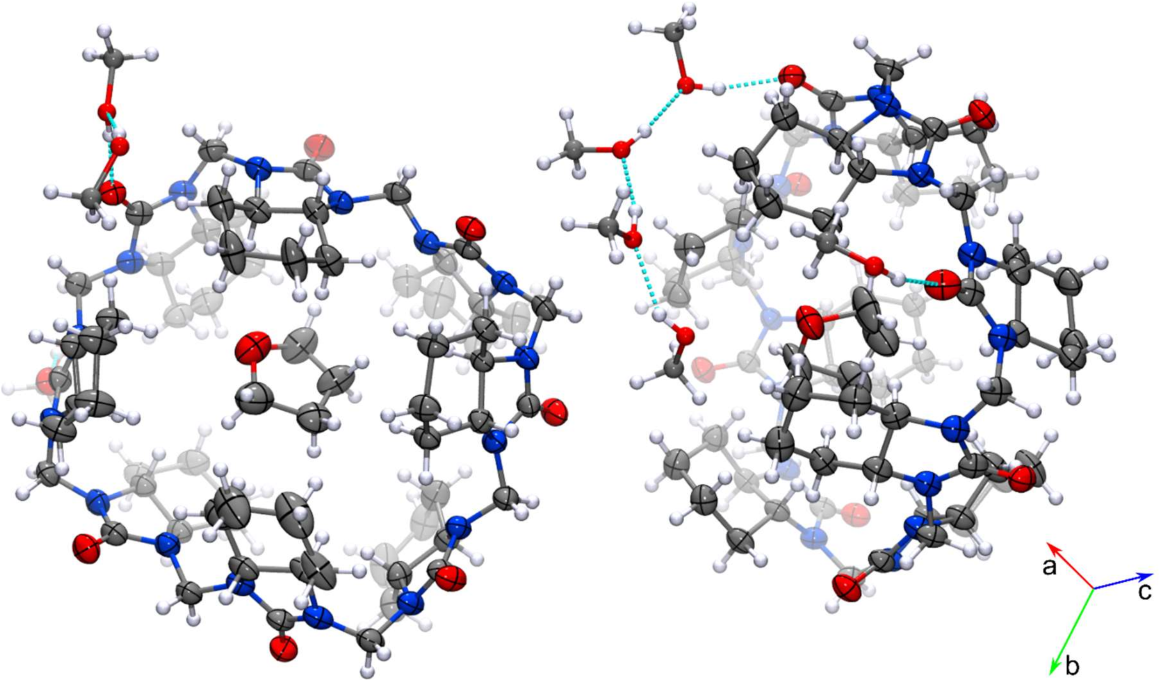

Figure S1. Asymmetric unit of THF@(*R*,*R*)-cycHC[8] (**CCDC 2069875**). Only the major disorder components of THF encapsulated within cycHC[8] are shown. Detailed view of all disorder components is shown on **Figure S6 A**. All atoms except methanol are shown with anisotropic displacement ellipsoids, at 50% probability level. Methanol molecules are drawn with a ball and stick model and hydrogen bonds are indicated with light blue dashed lines.

Crystallographic details for the 2,5-dihydrofuran@(*R*,*R*)-cycHC[8] complex

Colourless block-shaped single crystals were obtained from a methanol solution of (*R*,*R*)- cycHC[8] in the presence of 60 molar equivalents of 2,5-dihydrofuran. The asymmetric unit of the crystal structure contains two inclusion complexes of 2,5-dihydrofuran@(*R*,*R*)- cycHC[8], surrounded by solvent (methanol) molecules (**Figure S2**). The encapsulated 2,5-dihydrofuran molecules are disordered in the crystal structure. Both sites of 2,5-dihydrofuran were modelled with two disorder components, with total site occupancy reduced – to *s.o.f.* 0.75 for one complex and 0.5 in other. The relative occupancy of the disorder components was allowed to refine freely. The remaining electron density within the cycHC[8] cavities has no clear features, preventing full resolution the guest disorder. Minor unresolved disorder may include partial substitutional disorder by methanol molecules. Geometry of the resolved 2,5-dihydrofuran molecules was restrained using the SAME restraints in *SHELXL*. The anisotropic displacement parameters of the disorder components were restrained using SIMU restraints in *SHELXL*. Anti-bumping restraints were introduced using BUMP, to restrain the positions of minor disorder components (low *s.o.f.*) and avoid too close contacts of non-bonded atoms. The position of 8 methanol molecules could be resolved in the asymmetric unit with full occupancy. Of these, one methanol molecule was found to be disordered between two orientations, which were modelled using two disorder components with freely refined relative occupancies (*s.o.f*). The remaining solvent molecules (methanol) were too severely disordered to be adequately modelled and were therefore accounted for using the SQUEEZE algorithm of *PLATON* [9]. The electron density in the detected voids (probe radius 1.2 Å, 119 electrons in 526 Å^3 void, 7% of the unit cell volume) can include unresolved methanol and 2,5-dihydrofuran molecules.

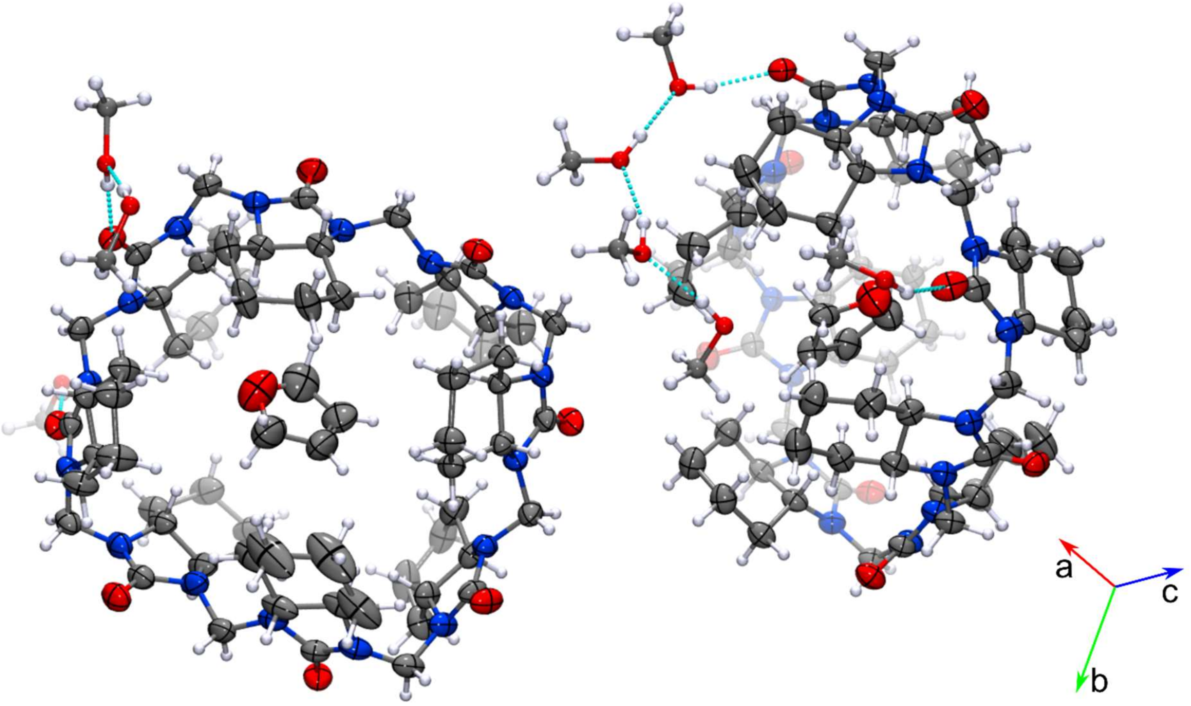

Figure S2. Asymmetric unit of 2,5-dihydrofuran@(*R*,*R*)-cycHC[8] (**CCDC 2069876**). Only major disorder components of the encapsulated 2,5-dihydrofuran are shown. Detailed view of all disorder components is shown on **Figure S6 B**. All atoms except methanol are shown with anisotropic displacement ellipsoids, at 50% probability level. Methanol molecules are drawn with a ball and stick model and hydrogen bonds are indicated with light blue dashed lines.

Crystallographic details for the 1,4-dioxane@(*R*,*R*)-cycHC[8] complex

Colourless plate-shaped single crystals were obtained from a methanol solution of (*R*,*R*)- cycHC[8] in the presence of 60 molar equivalents of 1,4-dioxane. The asymmetric unit of the crystal structure contains two inclusion complexes of 1,4-dioxane@(*R*,*R*)-cycHC[8], surrounded by solvent (methanol) molecules (**Figure S3**). One of the encapsulated 1,4-dioxane sites was modelled with two disorder components, with total site occupancy reduced to 0.75. The relative occupancy of the disorder components was allowed to refine freely. The minor disorder component(s) in the second 1,4-dioxane site could not be modelled adequately, therefore only the major disorder component was modelled with reduced fixed occupancy (*s.o.f* 0.5). Based on the featureless remaining electron density around the encapsulated 1,4-dioxanes, it is also possible that these sites are partially occupied by disordered methanol molecules. Geometry of the 1,4-dioxanes was restrained using the SAME restraints in *SHELXL*. The anisotropic displacement parameters of the disorder components were restrained using SIMU restraints in *SHELXL*. Anti-bumping restraints were introduced using BUMP, to restrain the positions of minor disorder components (low *s.o.f.*) and avoid too close contacts of non-bonded atoms. Four methanol molecules could be resolved in the asymmetric unit with full occupancy, and further two with reduced occupancies (*s.o.f.* 0.75). The remaining solvent molecules (methanol) are too severely disordered to be adequately modelled and were therefore accounted for using the SQUEEZE algorithm of *PLATON*. The electron density in the detected voids (probe radius 1.2 Å, 202 electrons in 755 Å^3 void, 9.5% of the unit cell volume) can contain unresolved methanol and 1,4-dioxane molecules.

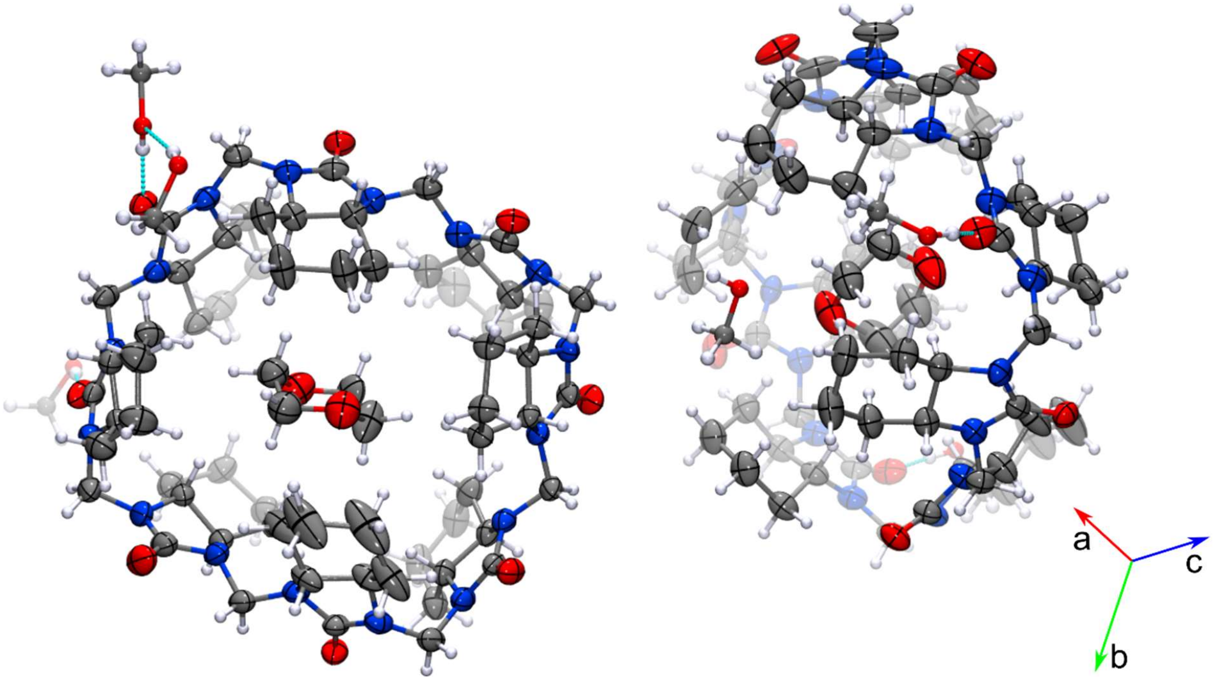

Figure S3. Asymmetric unit of 1,4-dioxane@(*R*,*R*)-cycHC[8] (**CCDC 2069877**). Only the major disorder components of the encapsulated 1,4-dioxane molecules are shown within cycHC[8]. Detailed view of all disorder components is shown on **Figure S6 C**. All atoms except methanol are shown with anisotropic displacement ellipsoids, at 50% probability level. Methanol molecules are drawn with a ball and stick model and hydrogen bonds are indicated with light blue dashed lines.

Crystallographic details for the 1,4-thioxane@(*R*,*R*)-cycHC[8] complex

Colourless plate-shaped single crystals were obtained from a methanol solution of (*R*,*R*)- cycHC[8] in the presence of 60 molar equivalents of 1,4-thioxane. The asymmetric unit of the crystal structure contains four inclusion complexes of 1,4-thioxane@(*R*,*R*)-cycHC[8], crystallized alongside solvent (methanol) molecules (**Figure S4**). All encapsulated 1,4-thioxane molecules are disordered within the cycHC[8] macrocycles. Two 1,4-thioxane sites could be modelled with two disorder components each, with full site occupancy and freely refined relative occupancy (*s.o.f*) of the disorder components. The minor disorder components in each of the two remaining 1,4-thioxane sites could not be modelled adequately, therefore only the major disorder components were modelled in these two sites with reduced fixed occupancy (*s.o.f* 0.75 and *s.o.f* 0.5). Based on the featureless remaining electron density around these two 1,4-thioxanes, it is possible that these sites are partially occupied by disordered methanol molecules. One cycHC[8] molecule was found to be disordered over two sites, and was thus modelled using two disorder components with freely refined relative occupancy (*s.o.f*). Geometry of the disordered parts were restrained using the SAME restraints in *SHELXL*, which restrain 1,2 and 1,3 bond distances to be equal to chemically identical molecules in the ordered parts of the structure. The anisotropic displacement parameters of the disorder components were restrained using SIMU restraints in *SHELXL*. Anti-bumping restraints were introduced using BUMP, to restrain the positions of minor disorder components (low *s.o.f.*) and avoid too close contacts of non-bonded atoms. The solvent molecules (methanol) are extensively disordered in this crystal structure and could not be adequately modelled. The contribution of the solvent molecules and any potentially co-crystallized non-encapsulated 1,4-thioxane molecules, was therefore accounted for using the SQUEEZE algorithm of *PLATON*. The electron density in the detected voids (probe radius 1.2 Å, 988 electrons in 3536 Å^3 void, 21% of the unit cell volume) can contain unresolved methanol and 1,4-thioxane molecules.

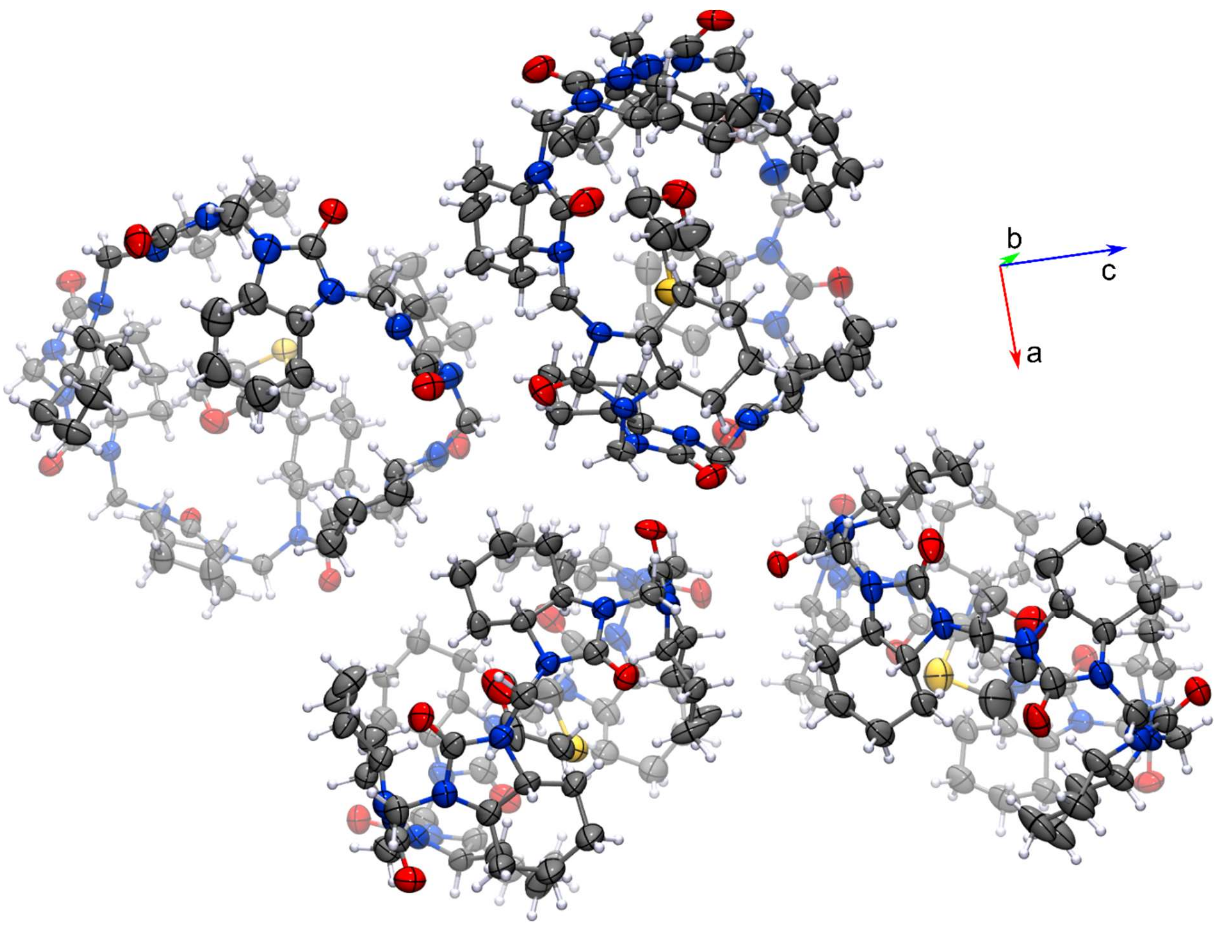

Figure S4. Asymmetric unit of 1,4-thioxane@(*R*,*R*)-cycHC[8] (**CCDC 2069878**). Only the major disorder components of the encapsulated 1,4-thioxane molecules are shown within each cycHC[8]. Detailed view of all disorder components is shown on **Figure S6 D**. All atoms are shown with anisotropic displacement ellipsoids, at 50% probability level. The minor disorder component of the single disordered cycHC[8] is not shown for the sake of clarity of the figure.

Crystallographic details for the 1,3-dithiolane@(*R*,*R*)-cycHC[8] complex

Colourless rod-shaped single crystals were obtained from a methanol solution of (*R*,*R*)- cycHC[8] in the presence of 60 molar equivalents of 1,3-dithiolane. The asymmetric unit of the crystal structure contains four inclusion complexes of 1,3-dithiolane@(*R*,*R*)-cycHC[8], crystallized alongside solvent (methanol) and one 1,3-dithiolane molecule (**Figure S5**). All but one of the encapsulated 1,3-dithiolane molecules are disordered within the cycHC[8] macrocycles and were each modelled using two disorder components. The relative site occupancy (*s.o.f*) of the disorder components was allowed to refine freely. The single resolved co-crystallized 1,3-dithiolane molecule (not encapsulated) appears also to be disordered, however the minor disorder component (*s.o.f* < 0.25) could not be modelled adequately, therefore only the major disorder component was modelled in this site with reduced occupancy (*s.o.f* 0.75). Half of one cycHC[8] molecule was also found disordered over two sites. The freely refined *s.o.f* of the host disorder components matches the relative *s.o.f* of the respective encapsulated disordered guest, indicating that the whole host-guest complex (one of the four in the asymmetric unit) is likely disordered between two orientations. Geometry of the disordered parts were restrained using the SAME restraints in *SHELXL*, where 1,2 and 1,3 bond distances were restrained to be equal to chemically identical molecules in the ordered parts of the

structure. The anisotropic displacement parameters of the disorder components were restrained using SIMU restraints in *SHELXL*. Anti-bumping restraints were introduced using BUMP, to restrain the positions of minor disorder components (low *s.o.f.*) and avoid too close contacts of non-bonded atoms. The solvent molecules (methanol) are extensively disordered in this crystal structure, so that the position of only three methanol molecules could be adequately resolved in the asymmetric unit. The contribution of the remaining solvent molecules, and any further co-crystallized 1,3-dithiolane molecules, was accounted for using the SQUEEZE algorithm of *PLATON*. The electron density in the voids (probe radius 1.2 Å, 848 electrons in 2938 Å^3 void, 18% of the unit cell volume) can contain unresolved methanol and 1,3-dithiolane molecules.

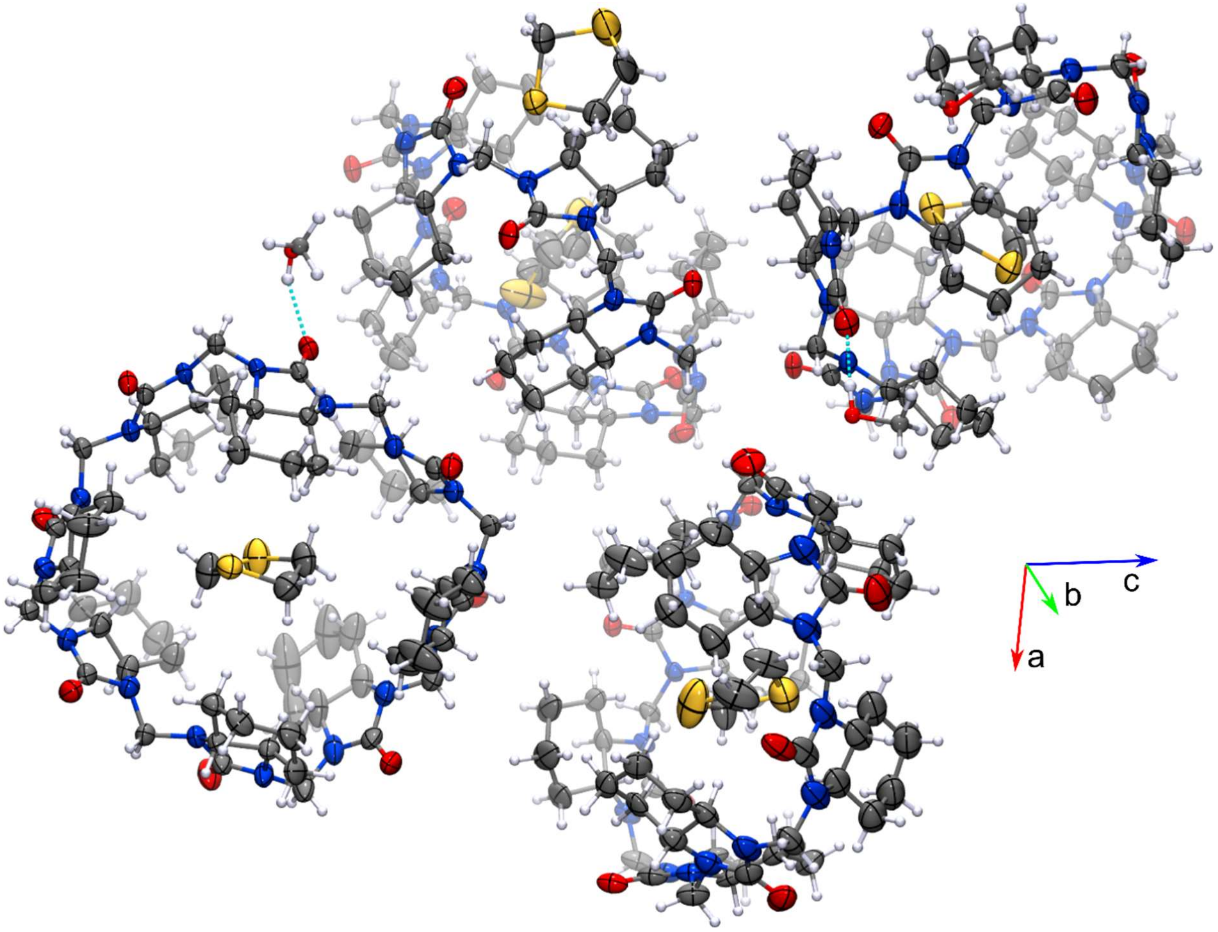

Figure S5. Asymmetric unit of 1,3-dithiolane@(*R*,*R*)-cycHC[8] (**CCDC 2069879**). Only the major disorder components of the encapsulated 1,3-dithiolane molecules are shown within each cycHC[8]. Detailed view of all disorder components is shown on **Figure S7**. All atoms except methanol are shown with anisotropic displacement ellipsoids, at 50% probability level. The resolved methanol molecules are drawn with a ball and stick model and hydrogen bonds are indicated with light blue dashed lines. The minor disorder component of the disordered cycHC[8] is not shown for the sake of clarity of the figure.

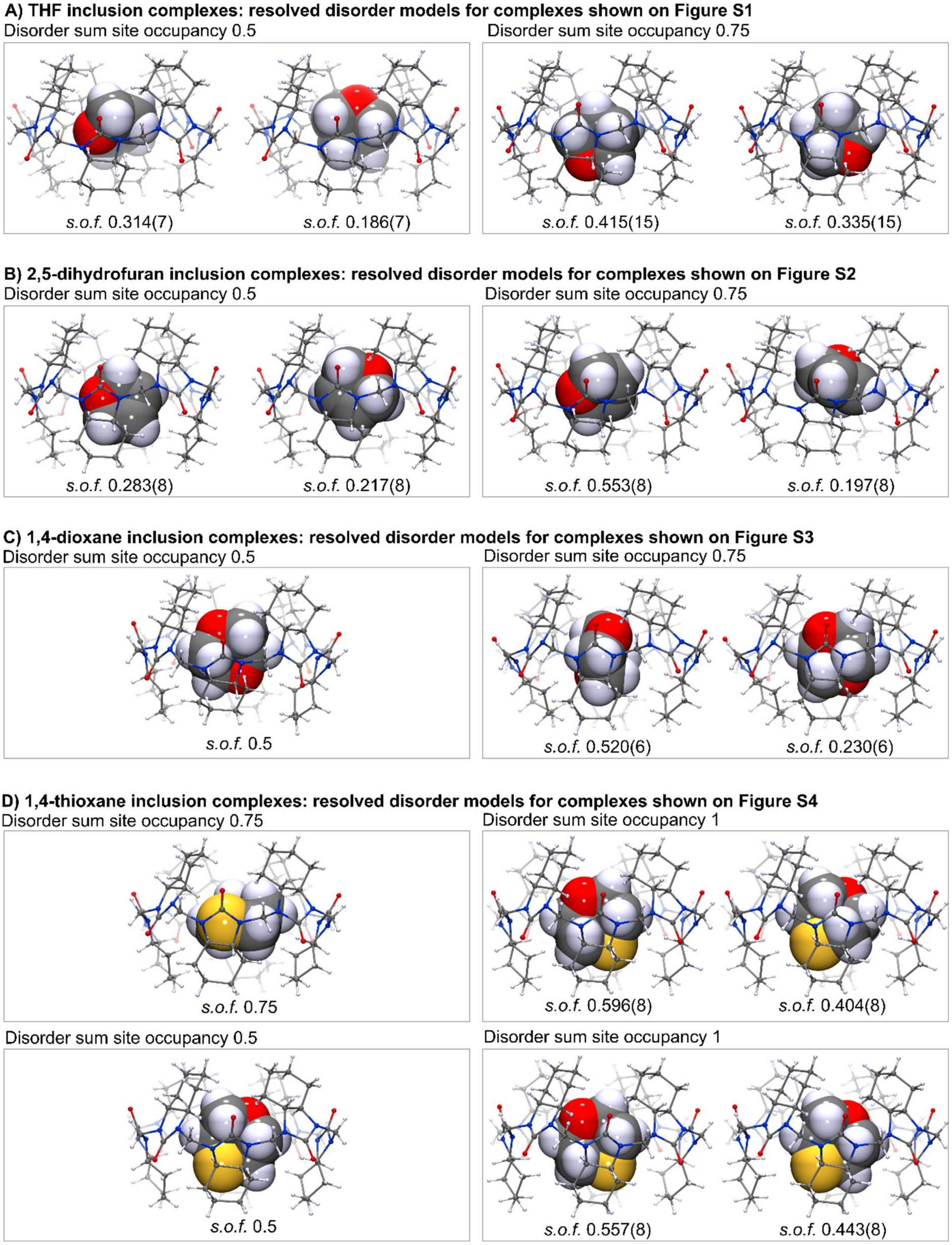

Figure S6. Inclusion complexes in the crystal structures of THF (A), 2,5-dihydrofuran (B), 1,4-dioxane (C) and 1,4-thioxane (D) showing the orientation of all resolved encapsulated guests in the asymmetric unit, with their respective site occupancy factors (s.o.f.).

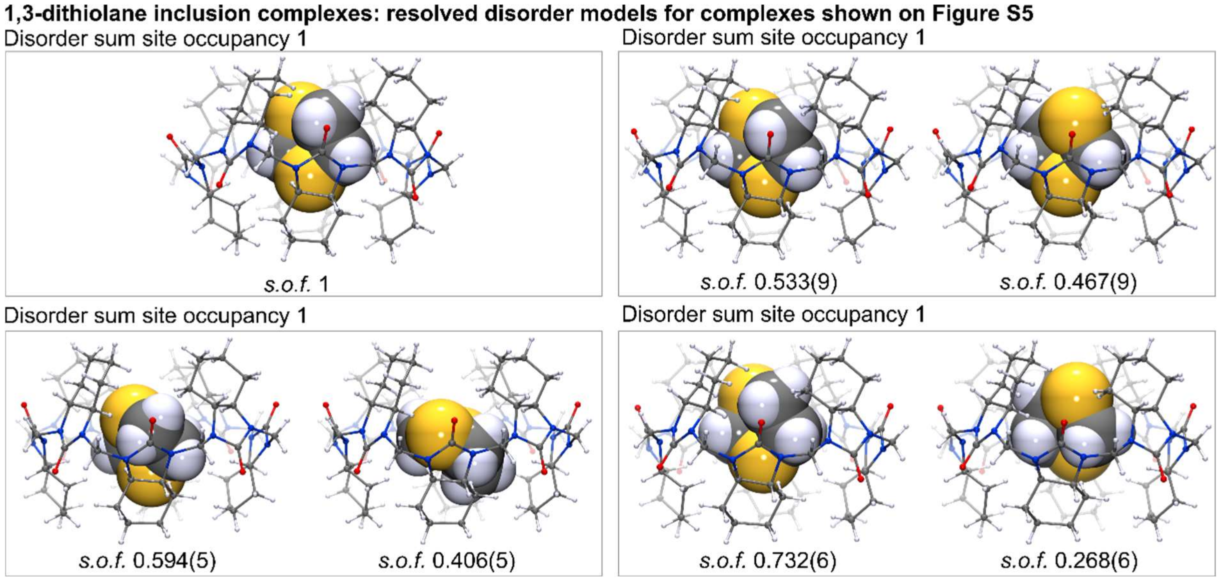

Figure S7. Inclusion complexes in the crystal structure of 1,3-dithiolane showing the orientation of the resolved encapsulated guest in the asymmetric unit, with the respective site occupancy factors (s.o.f.).

Table S2. Respective sizes for the host and guests based on crystal structures and the packing coefficients (PC) of heterocycle@cycHC[8] complexes

**Host**	V_{cavity}, **Å^3 [a]**	**Guest**	V_{guest}, **Å^3 [b]**	**Complex / PC[c]**
(*R*,*R*)-cycHC[8]	123	2,5-dihydrofuran	62.2 ± 0.2	A / 0.51
		tetrahydrofuran	66.0 ± 0.5	B / 0.54
		1,4-dioxane	74.6 ± 0.7	C / 0.61
		1,3-dithiolane	80.9 ± 0.7	D / 0.66
		1,4-thioxane	84.7 ± 0.6	E / 0.69

[a] The volume of (*R*,*R*)-cycHC[8] was determined in ref.(Kaabel and Aav, 2018) [b] The guest molecular volume was determined using the triangulated sphere model included in the Olex2 program package (Dolomanov et al., 2009), in which all atoms are approximated as isotropic spheres defined by the default Cambridge Structural Database (CSD) van der Waals (vdW) radii, and is given as an average of all guest molecules resolved in the asymmetric units of the respective crystal structures. [c] PC is the ratio between V_{guest} to V_{cavity}(host), reflecting the space filled by the encapsulated guest in the host cavity (Mecozzi and Rebek, 1998).

1.2. Complexation in solution

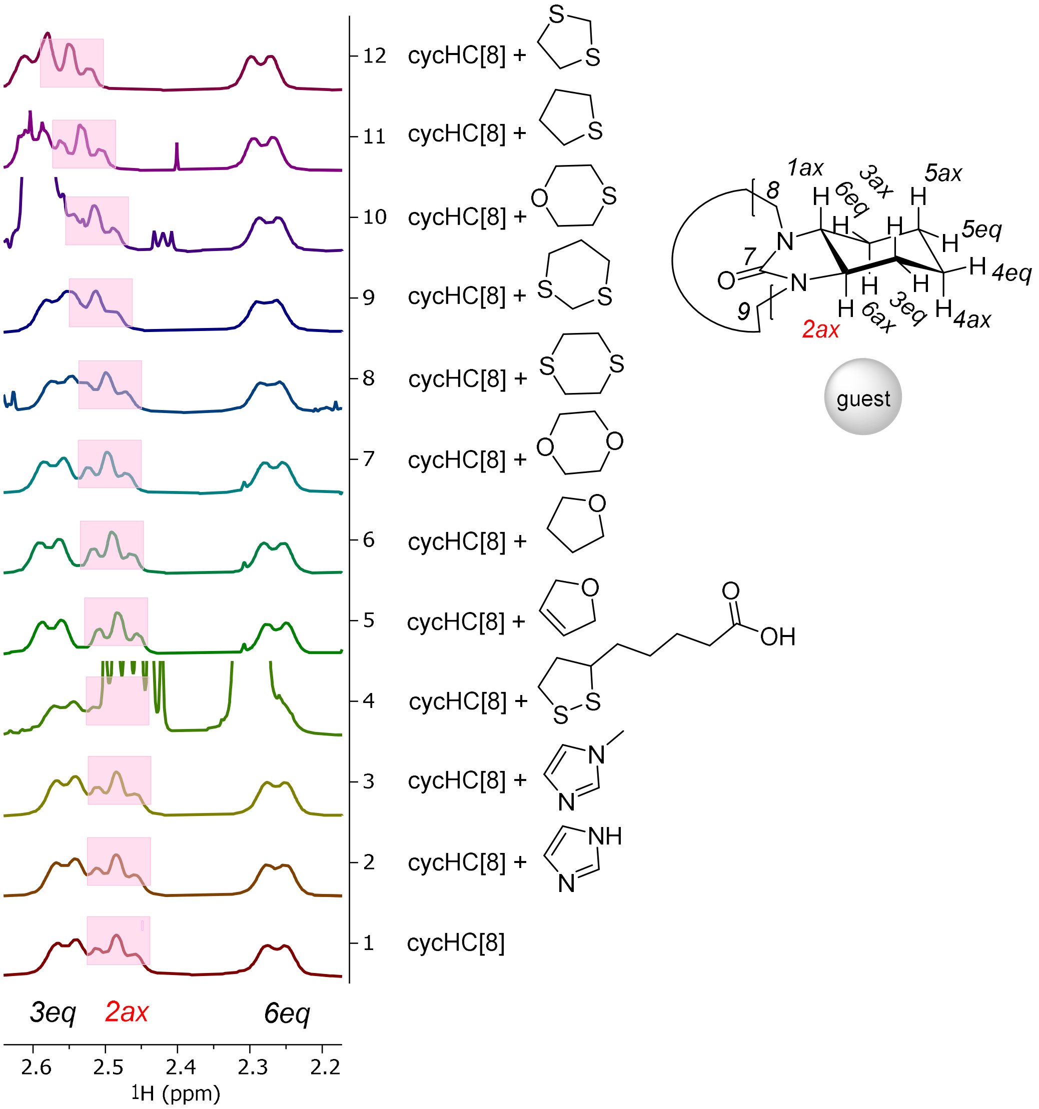

Figure S8A. Fragment of spectra from screening guests binding by ^{1}H NMR for cycHC[8] in CD_3OD (3 mM) upon addition of 60 eq. of respective guests.

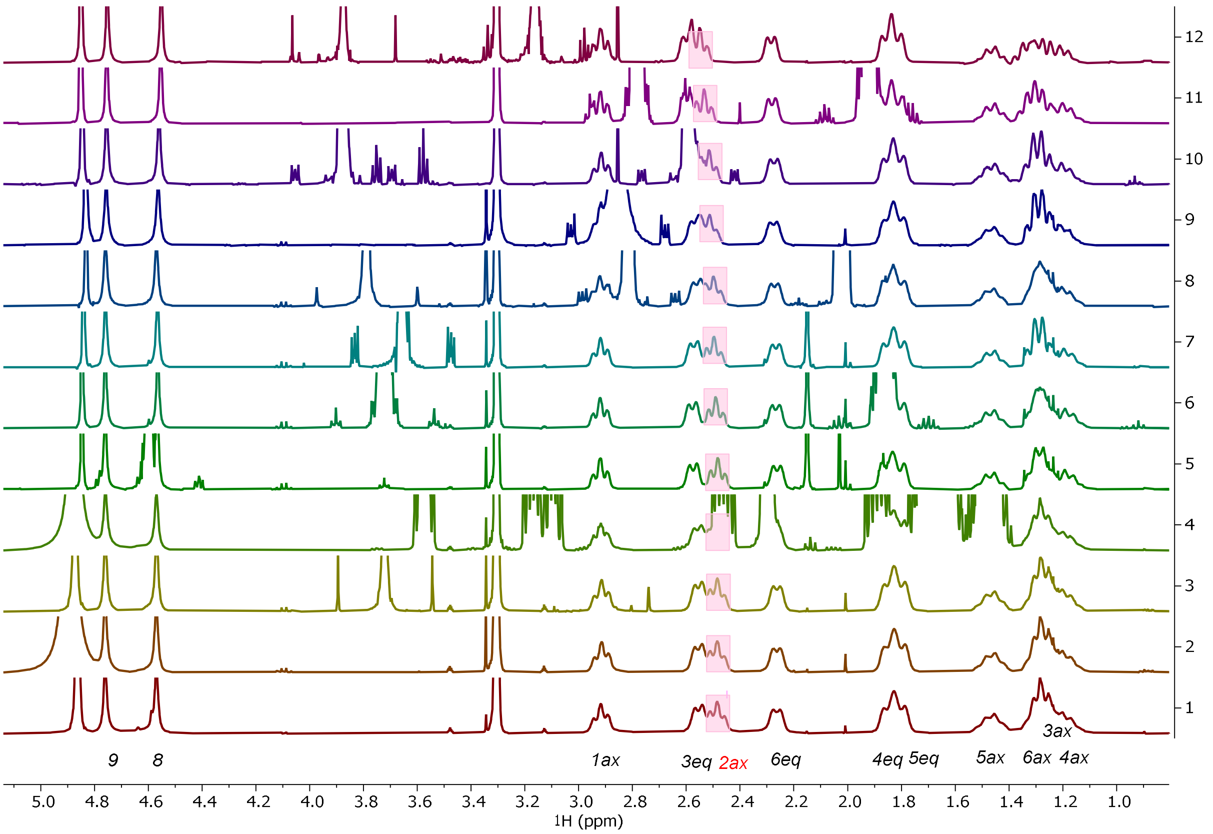

Figure S8B. Full spectra of screening guests binding by ^{1}H NMR for cycHC[8] in CD_3OD (3 mM) upon addition of 60 eq. of guests listed in Figure S8A.

Table S3. Screening of guest binding in CD_3OD using the 1H NMR chemical shift change (Δδ) of H2*ax* signal of 3mM cycHC[8] upon addition of 60 eq. of heterocycles

No	Guest	Δδ H_{2ax}, ppm	No	Guest	Δδ H_{2ax}, ppm
1	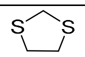	0.064	7	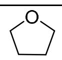	0.006
2	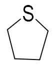	0.048	8	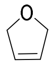	–
3	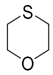	0.030	9	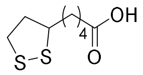	NA[a]
4	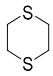	0.028	10	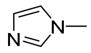	–
5	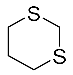	0.013	11	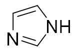	–
6	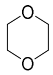	0.012			

[a] Guest signals overlapped with cycHC[8] H_{2ax} ^{1}H NMR signal.

1.3. Association constant measurements

1.3.1. NMR titration

Association constants for 1:1 inclusion complex of 1,3-dithiolane, 1,4-thioxane and 1,4-dioxane with cycHC[8] were determined from ^{1}H NMR titration (**Figures S9–S16**). Small portions of guest solution (360 mM 1,3-dithiolane, 2427 mM 1,4-thioxane and 2420–2537 mM 1,4-dioxane) were gradually added to the solution of cycHC[8] macrocycle (3 mM), causing its slow dilution. Shift of internal proton *2ax* was read out of all spectra. Exact values can be found in **Tables S4–S7**. Collected data was fitted with open online tool Bindfit at http://supramolecular.org/ (Thordarson, 2011; Brynn Hibbert and Thordarson, 2016).

Table S4. List of samples and corresponding concentrations (M) of cycHC[8] and 1,3- dithiolane in ^{1}H NMR titration, including values of chemical shift (ppm) from experiment and fitting with 1:1 binding model

Sample	$c_{cycHC[8]}$, M	$c_{1,3\text{-dithiolane}}$, M	Equivalent $[G]_0/[H]_0$	EXP cycHC[8]-*2ax*, ppm	FIT cycHC[8]-*2ax*, ppm
1	0.00300	0.00000	0.00	2.4892	2.4892
2	0.00300	0.00060	0.20	2.4901	2.4897
3	0.00299	0.00119	0.40	2.4902	2.4902
4	0.00299	0.00179	0.60	2.4908	2.4907
5	0.00298	0.00238	0.80	2.4914	2.4912
6	0.00298	0.00297	1.00	2.4920	2.4917
7	0.00297	0.00356	1.20	2.4930	2.4922
8	0.00297	0.00414	1.40	2.4931	2.4927
9	0.00296	0.00473	1.60	2.4936	2.4932
10	0.00296	0.00531	1.80	2.4940	2.4936
11	0.00295	0.00589	2.00	2.4946	2.4941
12	0.00293	0.00876	2.99	2.4967	2.4964
13	0.00290	0.01159	3.99	2.4983	2.4985
14	0.00286	0.01711	5.99	2.5020	2.5024
15	0.00277	0.02764	9.98	2.5086	2.5091
16	0.00257	0.05133	19.96	2.5211	2.5214
17	0.00240	0.07186	29.94	2.5300	2.5297

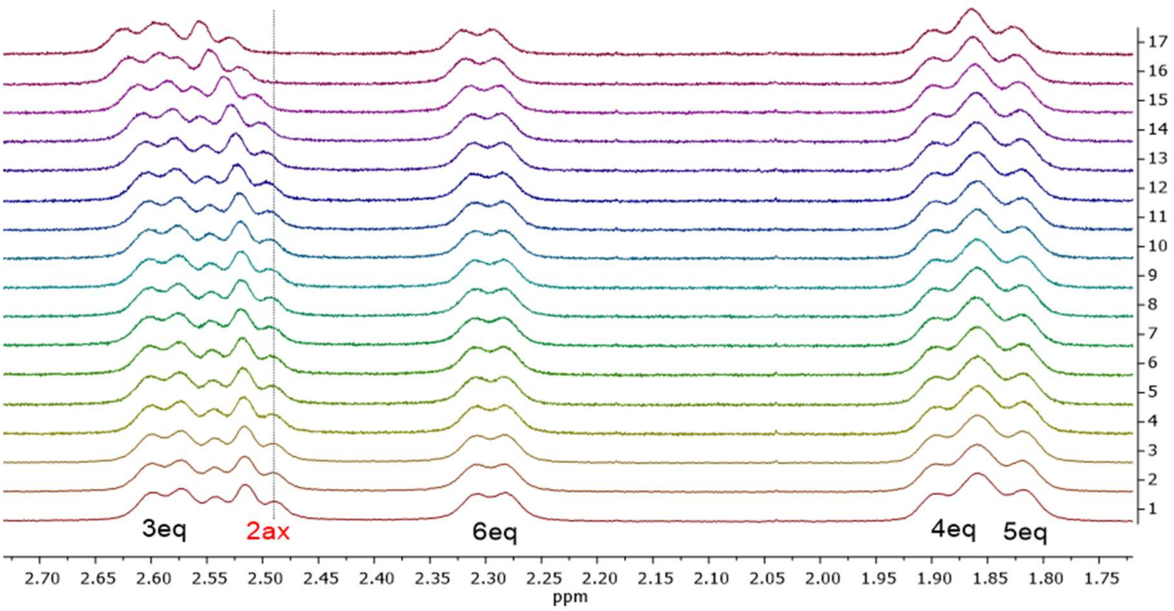

Figure S9. ^{1}H NMR titration spectra of cycHC[8] with additions of 1,3-dithiolane. Sample numbers correspond to the **Table S4** above.

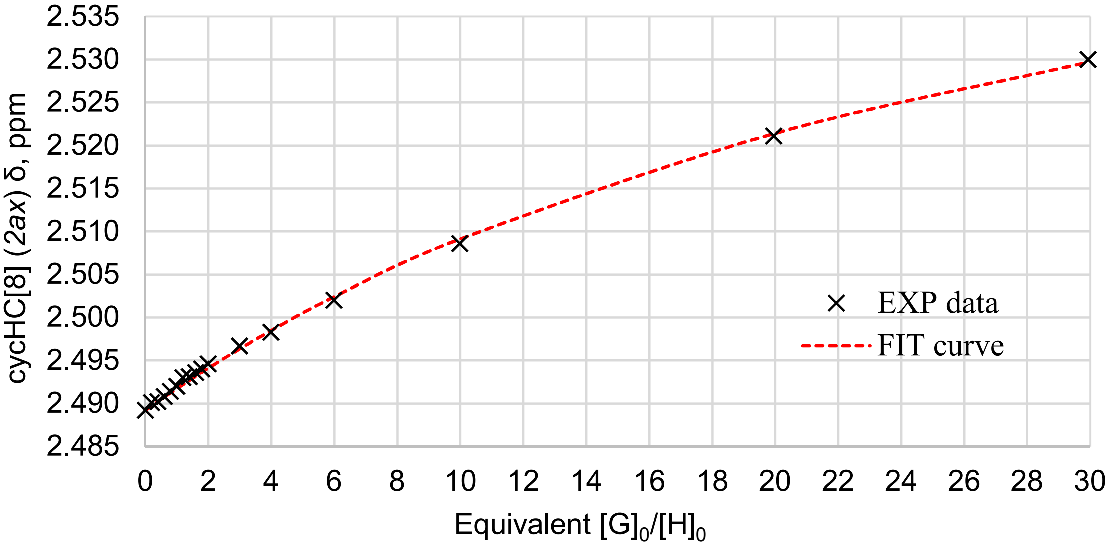

Figure S10. Binding isotherm and experimentaly obtained points for ^{1}H NMR titration of cycHC[8] with additions of 1,3-dithiolane. Fitting with 1:1 binding model provided $K_a = 7.9 \pm 0.2$ M^{-1}.

Table S5. List of samples and corresponding concentrations (M) of cycHC[8] and 1,4-thioxane in ^{1}H NMR titration, including values of chemical shift (ppm) from experiment and fitting with 1:1 binding model

Sample	$c_{cycHC[8]}$, M	$c_{1,4\text{-thioxane}}$, M	Equivalent $[G]_0/[H]_0$	EXP cycHC[8]-*2ax*, ppm	FIT cycHC[8]-*2ax*, ppm
1	0.00297	0.00000	0.00	2.4624	2.4624
2	0.00297	0.00562	1.89	2.4649	2.4637
3	0.00297	0.01422	4.79	2.4663	2.4656
4	0.00296	0.02912	9.83	2.4688	2.4688
5	0.00296	0.04384	14.82	2.4721	2.4718
6	0.00295	0.05879	19.90	2.4746	2.4747
7	0.00295	0.08853	30.06	2.4803	2.4799
8	0.00294	0.11833	40.29	2.4845	2.4846
9	0.00292	0.17756	60.80	2.4932	2.4926
10	0.00290	0.23736	81.76	2.4988	2.4993
11	0.00289	0.29741	103.04	2.5043	2.5050
12	0.00284	0.45214	159.06	2.5163	2.5163
13	0.00280	0.59831	213.59	2.5242	2.5238

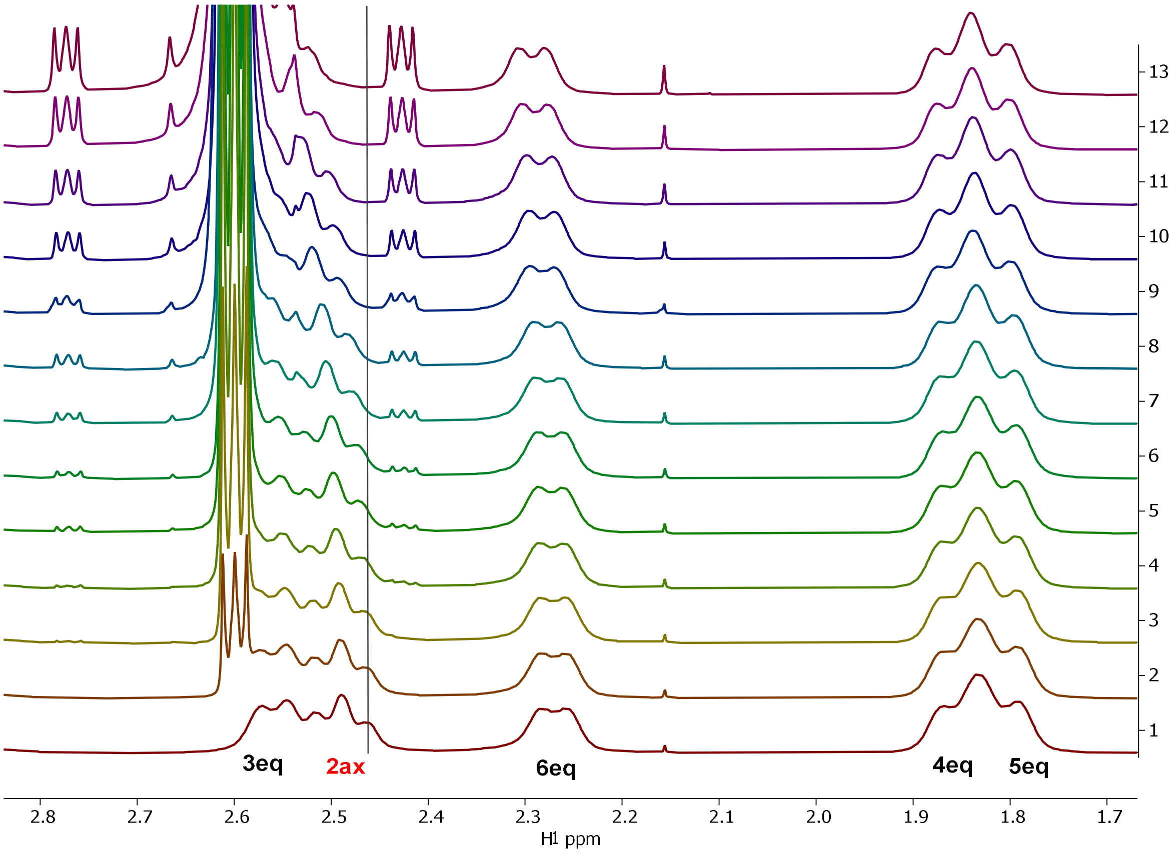

Figure S11. ^{1}H NMR titration spectra of cycHC[8] with additions of 1,4-thioxane. Sample numbers correspond to the **Table S5** above.

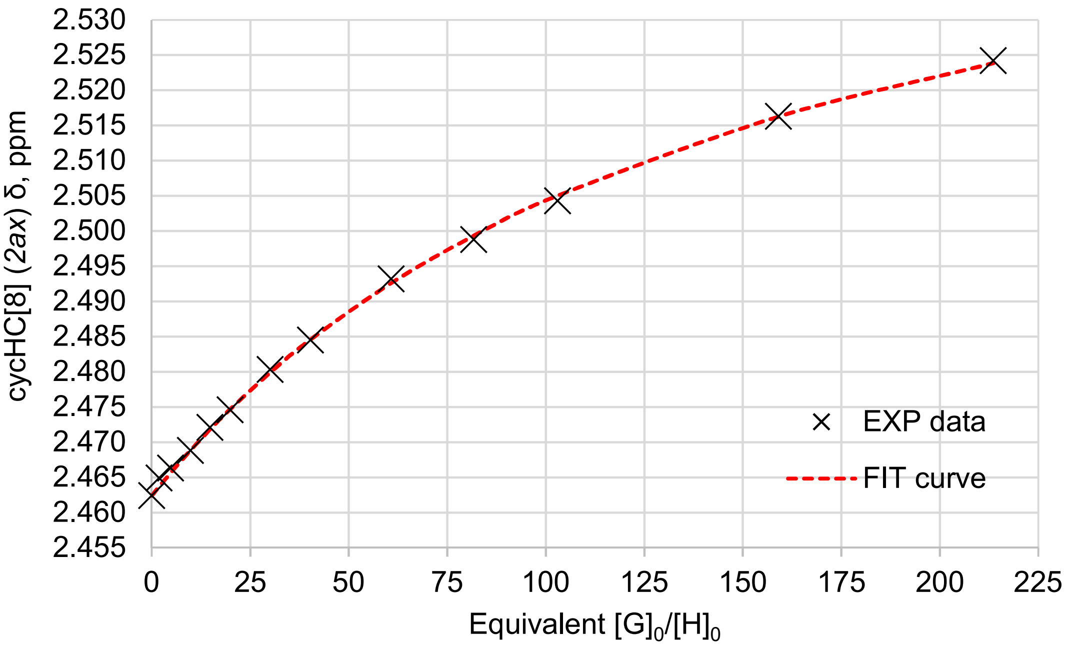

Figure S12. Binding isotherm and experimentaly obtained points for ^{1}H NMR titration of cycHC[8] with additions of 1,4-thioxane. Fitting with 1:1 binding model provided $K_a = 2.18 \pm 0.04$ M^{-1}.

Table S6. List of samples and corresponding concentrations (M) of cycHC[8] and 1,4- dioxane in ^{1}H NMR titration, including values of chemical shift (ppm) from experiment and fitting with 1:1 binding model

Sample	$c_{cycHC[8]}$, M	$c_{1,4\text{-dioxane}}$, M	Equivalent $[G]_0/[H]_0$	EXP cycHC[8]-*2ax*, ppm	FIT cycHC[8]-*2ax*, ppm
1	0.00300	0.00000	0.00	2.4633	2.4633
2	0.00300	0.00588	1.96	2.4636	2.4639
3	0.00300	0.01486	4.96	2.4647	2.4647
4	0.00299	0.02999	10.03	2.4663	2.4661
5	0.00299	0.04494	15.05	2.4681	2.4674
6	0.00298	0.06015	20.17	2.4691	2.4687
7	0.00297	0.09000	30.27	2.4718	2.4710
8	0.00296	0.11997	40.47	2.4731	2.4731
9	0.00295	0.18011	61.13	2.4767	2.4768
10	0.00293	0.24105	82.30	2.4789	2.4800
11	0.00291	0.30243	103.90	2.4829	2.4828
12	0.00286	0.46532	162.53	2.4887	2.4886
13	0.00282	0.60608	214.80	2.4924	2.4924
14	0.00273	0.90539	331.17	2.4978	2.4979
15	0.00265	1.20386	454.91	2.5019	2.5016
16	0.00256	1.50042	586.23	2.5042	2.5042

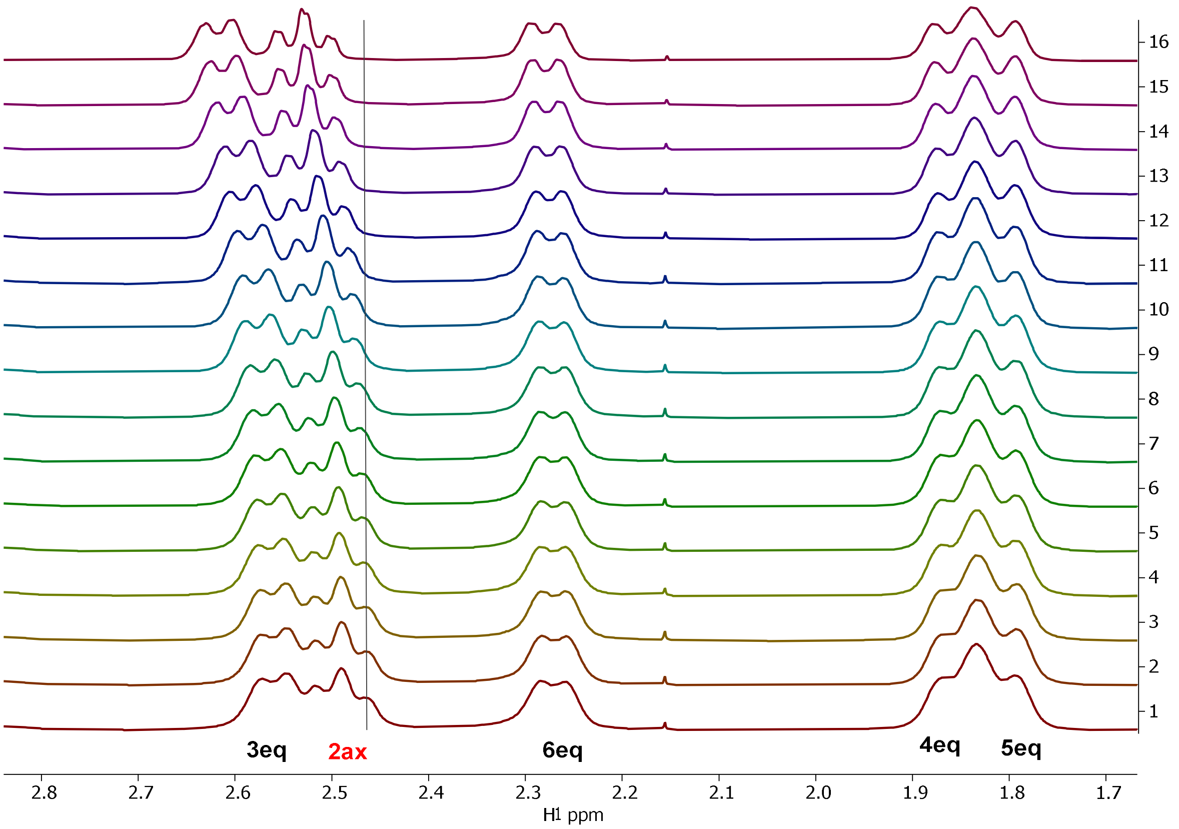

Figure S13. ^{1}H NMR titration spectra of cycHC[8] with additions of 1,4-dioxane. Sample numbers correspond to the **Table S6** above.

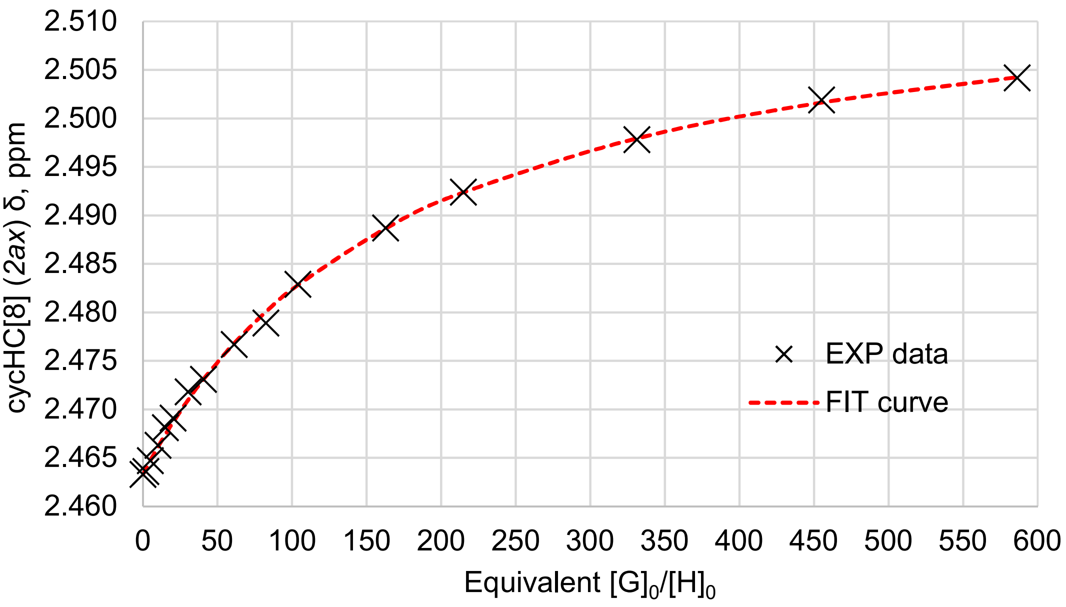

Figure S14. Binding isotherm and experimentaly obtained points for ^{1}H NMR titration of cycHC[8] with additions of 1,4-dioxane. Fitting with 1:1 binding model provided $K_a = 1.75 \pm 0.05$ M^{-1}.

Table S7. List of samples and corresponding concentrations (M) of cycHC[8] and 1,4- dioxane in ^{1}H NMR titration, including values of chemical shift (ppm) from experiment and fitting with 1:1 binding model

Sample	$c_{cycHC[8]}$, M	$c_{1,4\text{-dioxane}}$, M	Equivalent $[G]_0/[H]_0$	EXP cycHC[8]-*2ax*, ppm	FIT cycHC[8]-*2ax*, ppm
1	0.00295	0.00000	0.00	2.4891	2.4891
2	0.00295	0.00604	2.05	2.4899	2.4897
3	0.00294	0.01504	5.11	2.4909	2.4906
4	0.00294	0.02989	10.16	2.4923	2.4920
5	0.00294	0.04455	15.17	2.4938	2.4934
6	0.00293	0.05904	20.13	2.4947	2.4946
7	0.00293	0.08862	30.28	2.4969	2.4970
8	0.00292	0.11802	40.42	2.4993	2.4992
9	0.00291	0.17672	60.80	2.5031	2.5030
10	0.00289	0.23744	82.10	2.5061	2.5064
11	0.00288	0.29496	102.46	2.5092	2.5092
12	0.00284	0.44262	155.60	2.5148	2.5148
13	0.00281	0.58860	209.42	2.5192	2.5190
14	0.00274	0.88012	320.88	2.5245	2.5248
15	0.00267	1.17698	440.19	2.5282	2.5287
16	0.00261	1.47008	564.19	2.5319	2.5314

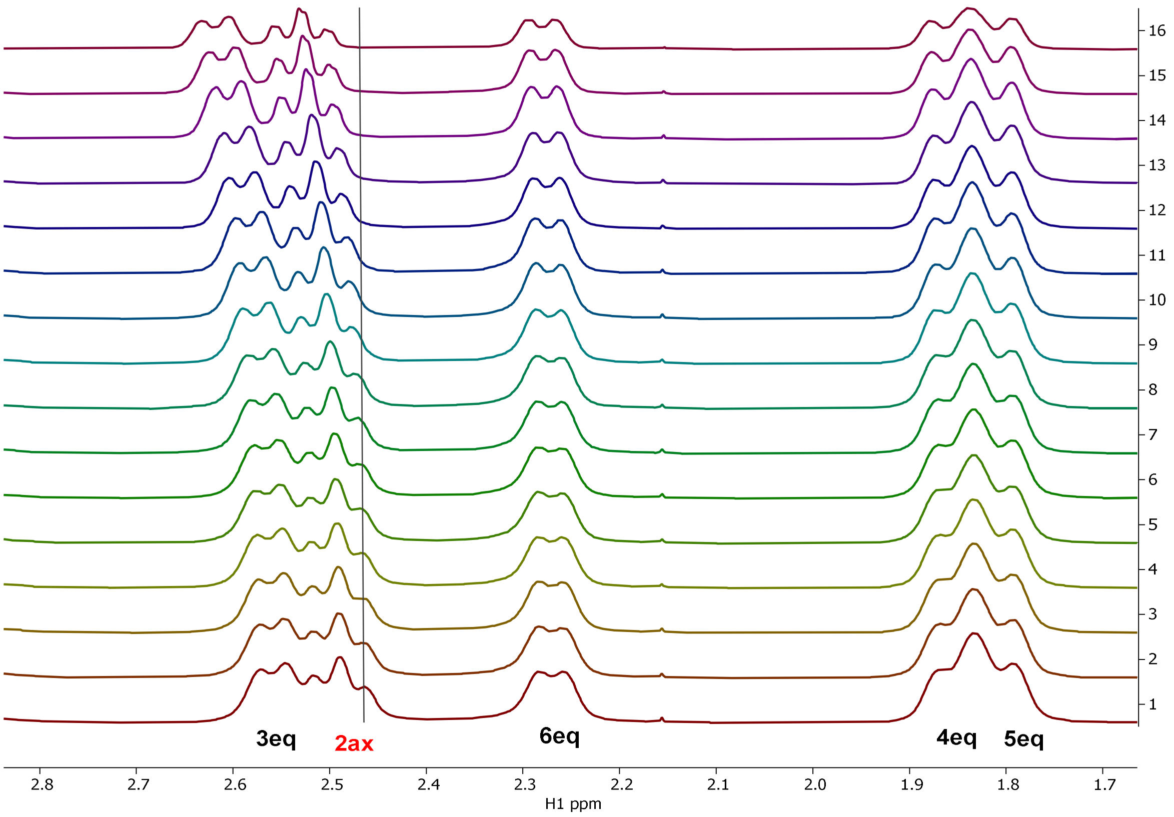

Figure S15. ^{1}H NMR titration spectra of cycHC[8] with additions of 1,4-dioxane. Sample numbers correspond to the **Table S7** above.

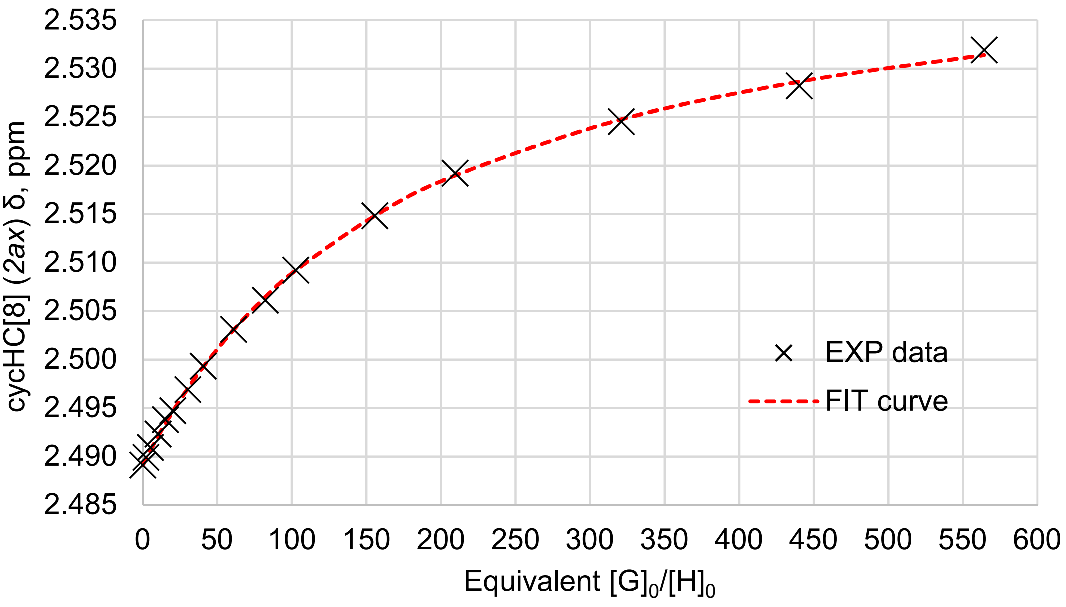

Figure S16. Binding isotherm and experimentaly obtained points for ^{1}H NMR titration of cycHC[8] with additions of 1,4-dioxane. Fitting with 1:1 binding model provided $K_a = 1.78 \pm 0.03$ M^{-1}.

1.3.2. Isothermal calorimetric titration

The titration was carried out by addition of 0.8 µL or 1.2 µL portions of guest compound solution to cycHC[8] solution in the cell with 70 s, 90 s or 120 s intervals. Some experiments started with longer spacing, which was adjusted during the measurement to shorten the total time of titration while leaving enough time for heat stabilization between additions. The heat of dilution was obtained by introduction of guest solution to the solvent (CH_3OH or CH_3OH : H_2O mixtures). The first smaller addition (0.4 µL) used to compensate diffusion of guest compound from the injector during system equilibration was discarded previous to fitting procedure. Prior to data analysis, the heat of dilution was subtracted from the corresponding total heat of the interaction. To allow a determination of low association constants (up to 10^2 M^{-1}) a high concentrations of guest (up to 1 M) were employed which lead to heat of dilutions significantly larger than heat produced by formation of complex. The obtained data was processed by MicroCal PEAQ-ITC analysis software (Malvern) and fitted using one set of sites binding model. Due to the combination of macrocycle's limited solubility in polar solvents and its weak binding of heterocyclic guest it is not possible to obtain data showing typical S-shaped titration curve. However, the evaluation (Schmidtchen, 2006) of weak binding data is possible thanks to the known 1:1 binding stoichiometry (Turnbull and Daranas, 2003), which was applied as an invariable parametre (N, number of sites = 1) during the evaluation of all ITC measurements.

The strongest binded guest 1,3-dithiolane was titrated at various CH_3OH : H_2O solvent mixtures to extrapolate the strength of binding in pure water. Unfortunately, it appeared to be impossible. Decreasing miscibility of 1,3-dithiolane in increasing content of water and low solubility of macrocycle in polar solvents were main limited factors for precise determination of association constants and enthalphy. Parallel titrations often provided significantly different results and determination of precise values would require excessive amount of measurements. Moreover, extrapolation of association constant to pure water would also require experimental values from solvent mixtures containing excess of water but macrocycle has only submilimolar solubility in CH_3OH : H_2O mixtures containing over 50% of water.

1,3-dithiolane binding to cycHC[8] in CH_3OH

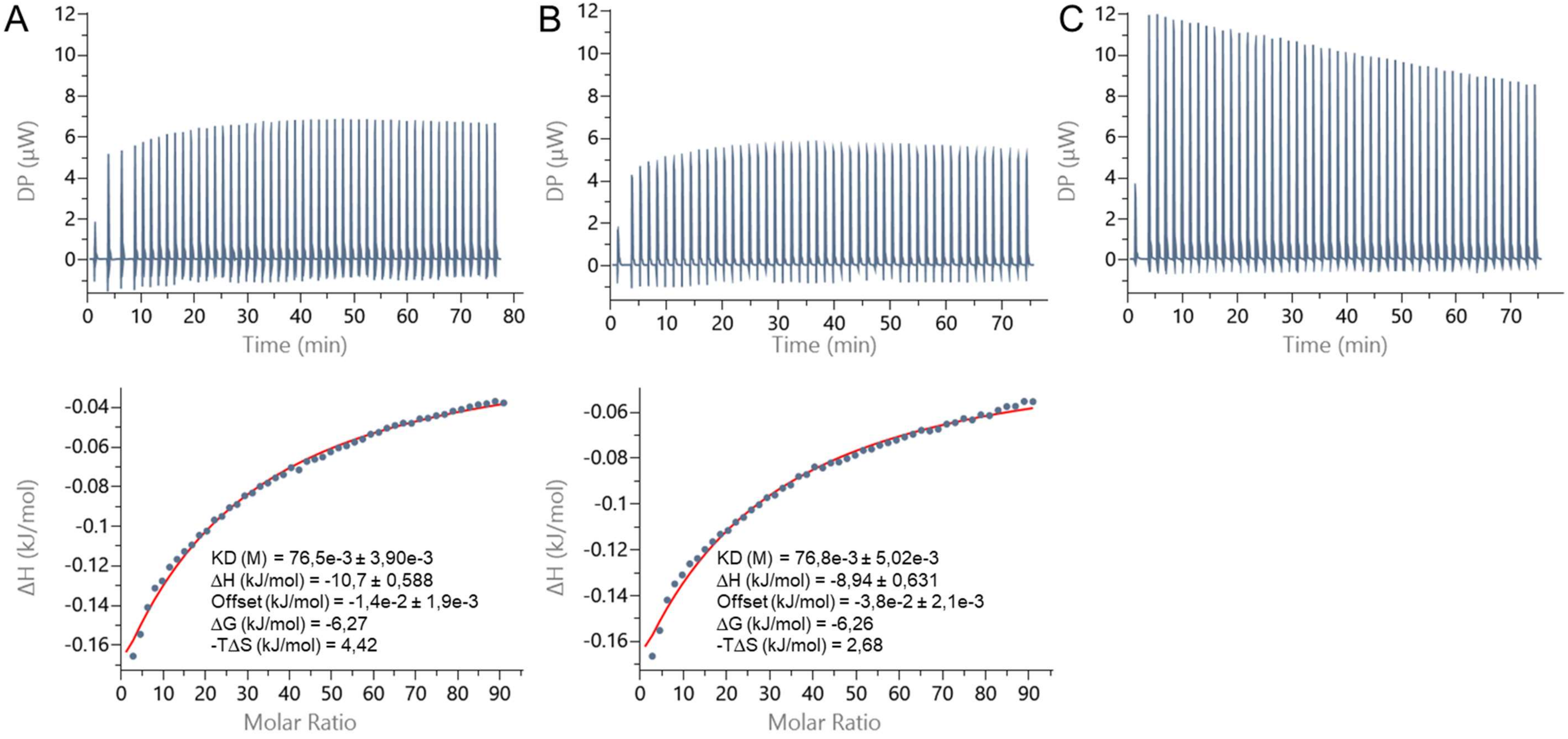

Figure S17. ITC of 1,3-dithiolane binding to cycHC[8] in CH_3OH at 293.35 K (A, B) and control experiment (C). *Top*: Data obtained from the sequential injections of 0.8 μL of 1,3-dithiolane (0.49 M) to cycHC[8] (1.1 mM) (A,B) or to the pure solvent (C) with 90 s spacing. *Bottom*: Plot of the total heat released as a function of total guest concentration for the titration shown in the upper panel. The red solid line represents least-squares fit of the data. Note: subtracted dilution heat (C) is large due to a high concentration of guest solution.

1,3-dithiolane binding to cycHC[8] in $CH_3OH:H_2O$ 80:20 mixture

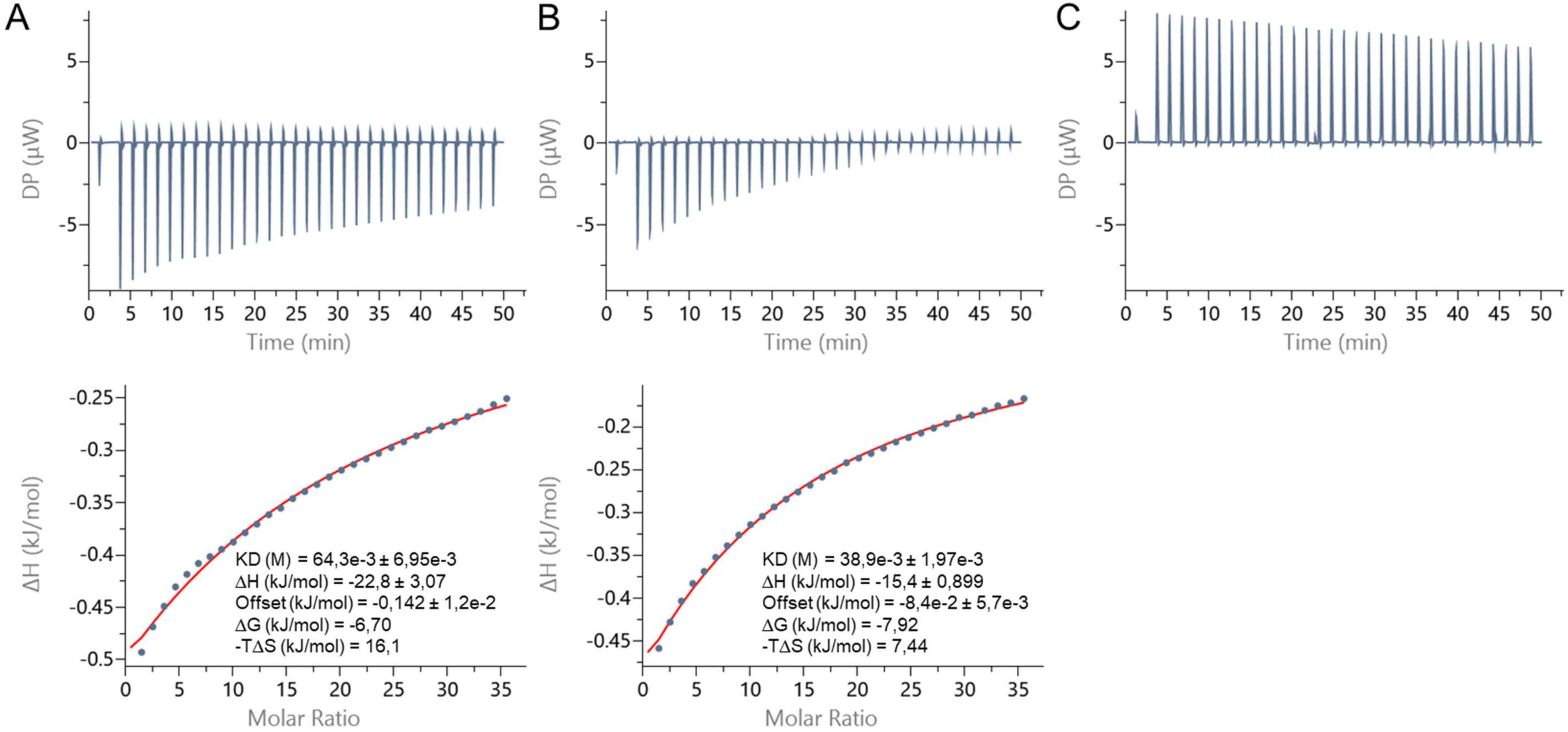

Figure S18. ITC of 1,3-dithiolane binding to cycHC[8] in CH_3OH : H_2O 80:20 mixture at 293.35 K (A, B) and control experiment (C). *Top*: Data obtained from the sequential injections of 1.2 μL of 1,3-dithiolane (0.18 M) to cycHC[8] (1.00 mM) (A,B) or to the pure solvent mixture (C) with 90 s spacing. *Bottom*: Plot of the total heat released as a function of total guest concentration for the titration shown in the upper panel. The red solid line represents least-squares fit of the data. Note: subtracted dilution heat (C) is large due to a high concentration of guest solution. Additionally, the difference of fitted results from parallel measurements can be attributed to lower achievable excess of guest (maximum molar ratio) caused by its lower miscibility in the solvent mixture containing water.

1,3-dithiolane binding to cycHC[8] in CH_3OH:H_2O 65:35 mixture

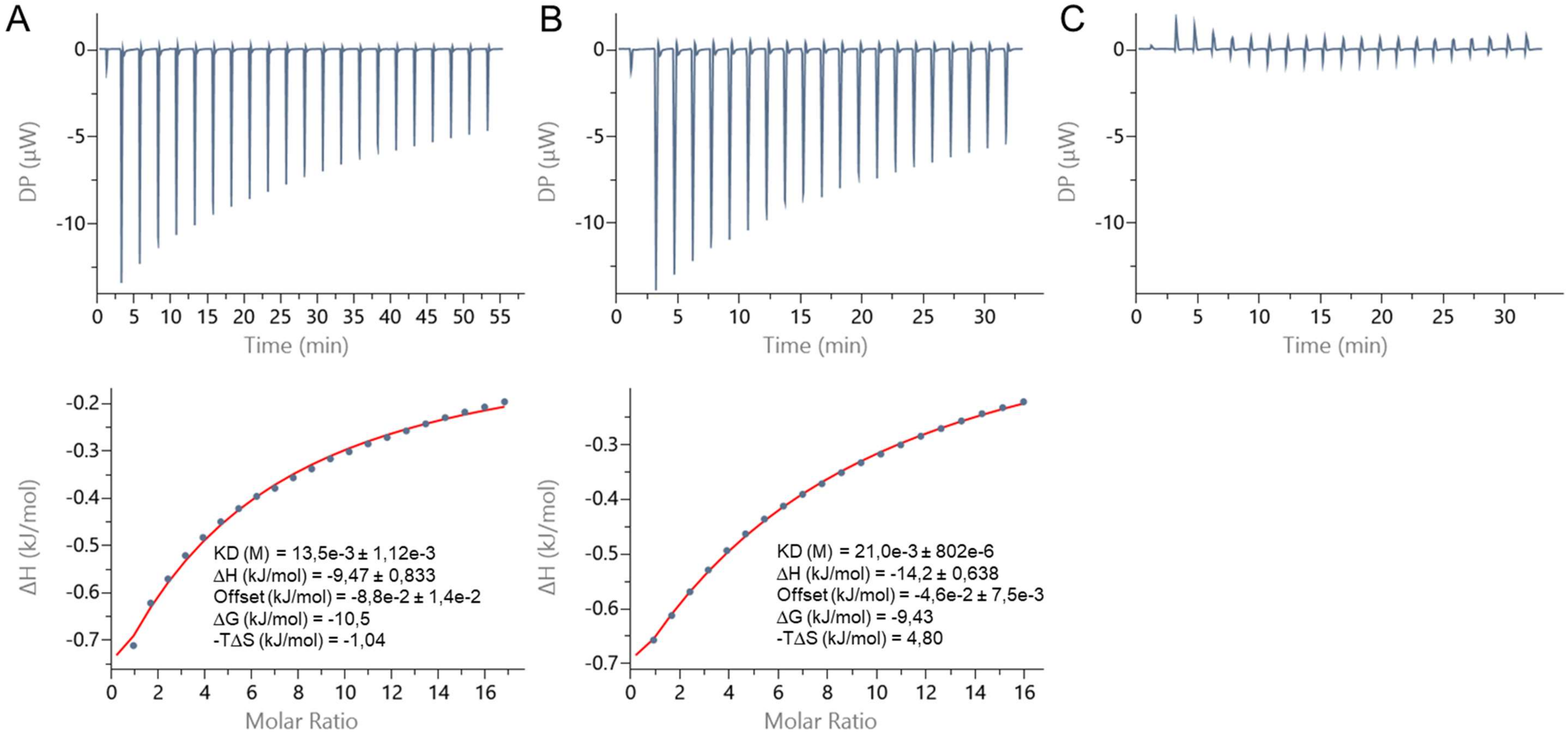

Figure S19. ITC of 1,3-dithiolane binding to cycHC[8] in CH_3OH : H_2O 65:35 mixture at 293.35 K (A, B) and control experiment (C). *Top*: Data obtained from the sequential injections of 1.8 µL of 1,3-dithiolane (0.0838 M) to cycHC[8] (1.00 mM) (A,B) or to the pure solvent mixture (C) with 150 s or 90 s spacing. *Bottom*: Plot of the total heat released as a function of total guest concentration for the titration shown in the upper panel. The red solid line represents least-squares fit of the data. Note: subtracted dilution heat (C) is negligible in comparison to heat produced by complexation event.

1,3-dithiolane binding to cycHC[8] in CH_3OH:H_2O 50:50 mixture

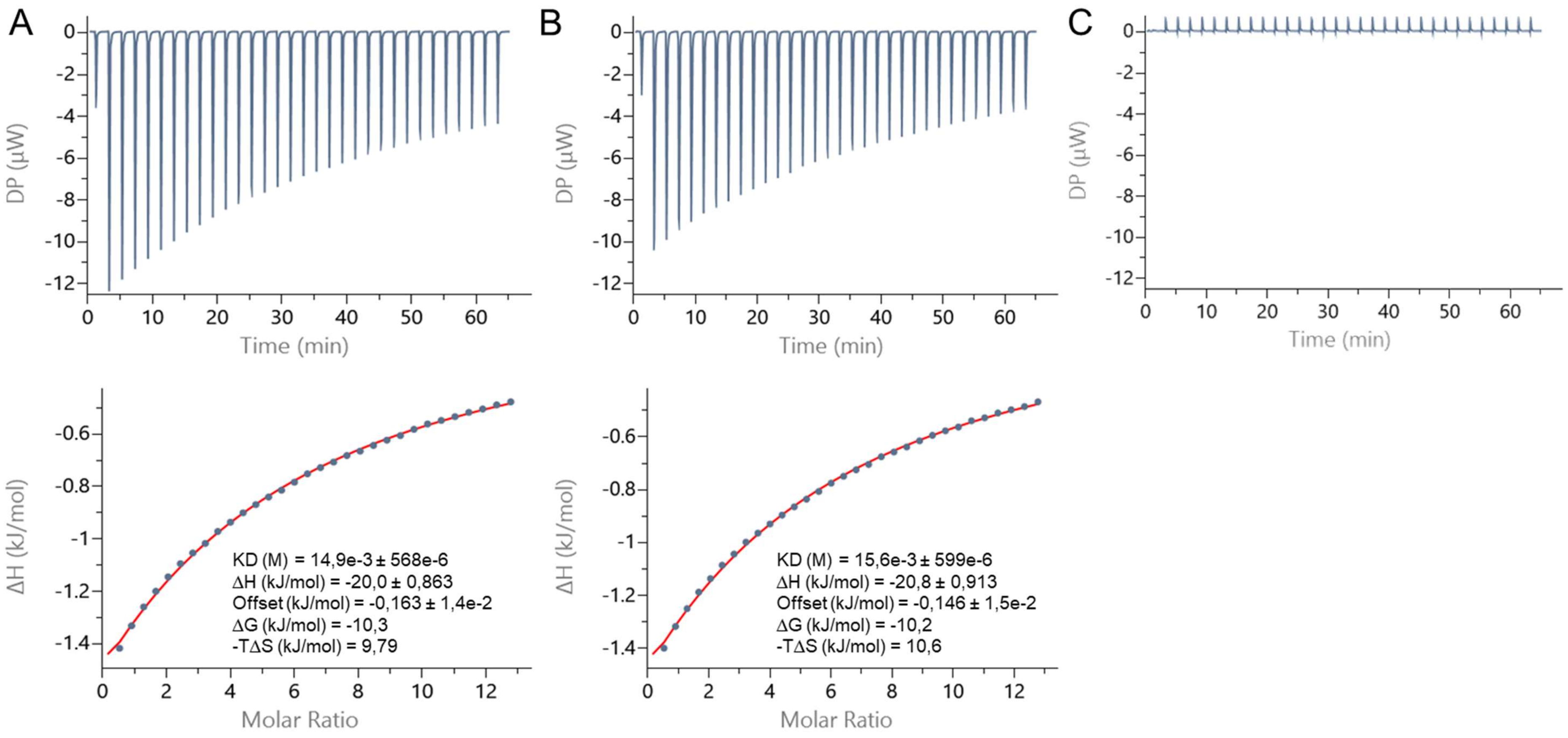

Figure S20. ITC of 1,3-dithiolane binding to cycHC[8] in CH_3OH : H_2O 50:50 mixture at 293.35 K (A, B) and control experiment (C). *Top*: Data obtained from the sequential injections of 1.2 μL of 1,3-dithiolane (0.0667 M) to cycHC[8] (1.03 mM) (A,B) or to the pure solvent mixture (C) with 120 s spacing. *Bottom*: Plot of the total heat released as a function of total guest concentration for the titration shown in the upper panel. The red solid line represents least-squares fit of the data. Note: subtracted dilution heat (C) is negligible in comparison to heat produced by complexation event.

1,4-thioxane binding to cycHC[8] in CH_3OH

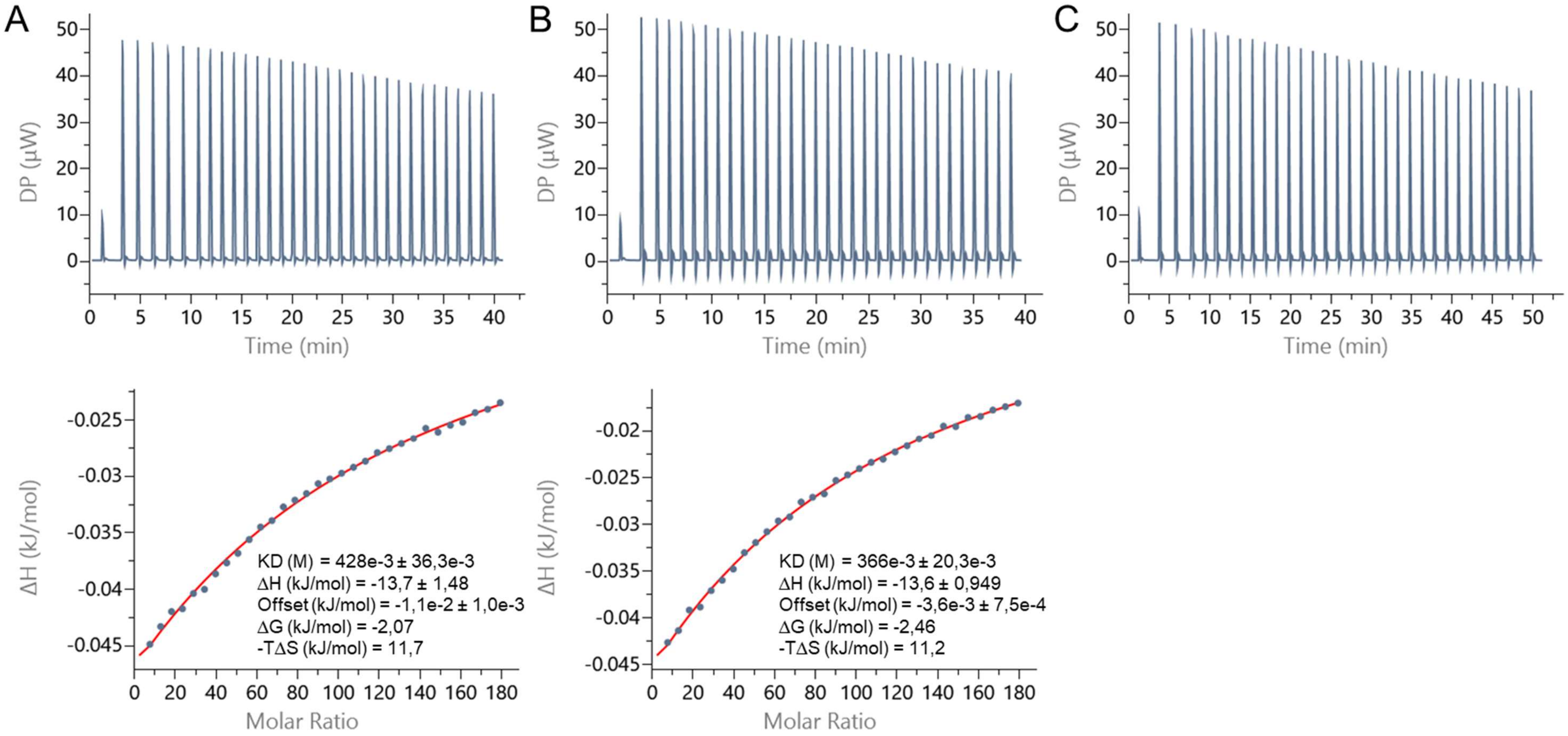

Figure S21. ITC of 1,4-thioxane binding to cycHC[8] in CH_3OH at 293.35 K (A, B) and control experiment (C). *Top:* Data obtained from the sequential injections of 1.2 μL of 1,4-thioxane (1 M) to cycHC[8] (1.1 mM) (A,B) or to the pure solvent (C) with 70 s or 90 s spacing. *Bottom*: Plot of the total heat released as a function of total ligand concentration for the titration shown in the upper panel. The red solid line represents least-squares fit of the data. Note: subtracted dilution heat (C) is large due to a high concentration of guest solution.

1,4-thioxane binding to cycHC[8] in $CH_3OH:H_2O$ 50:50 mixture

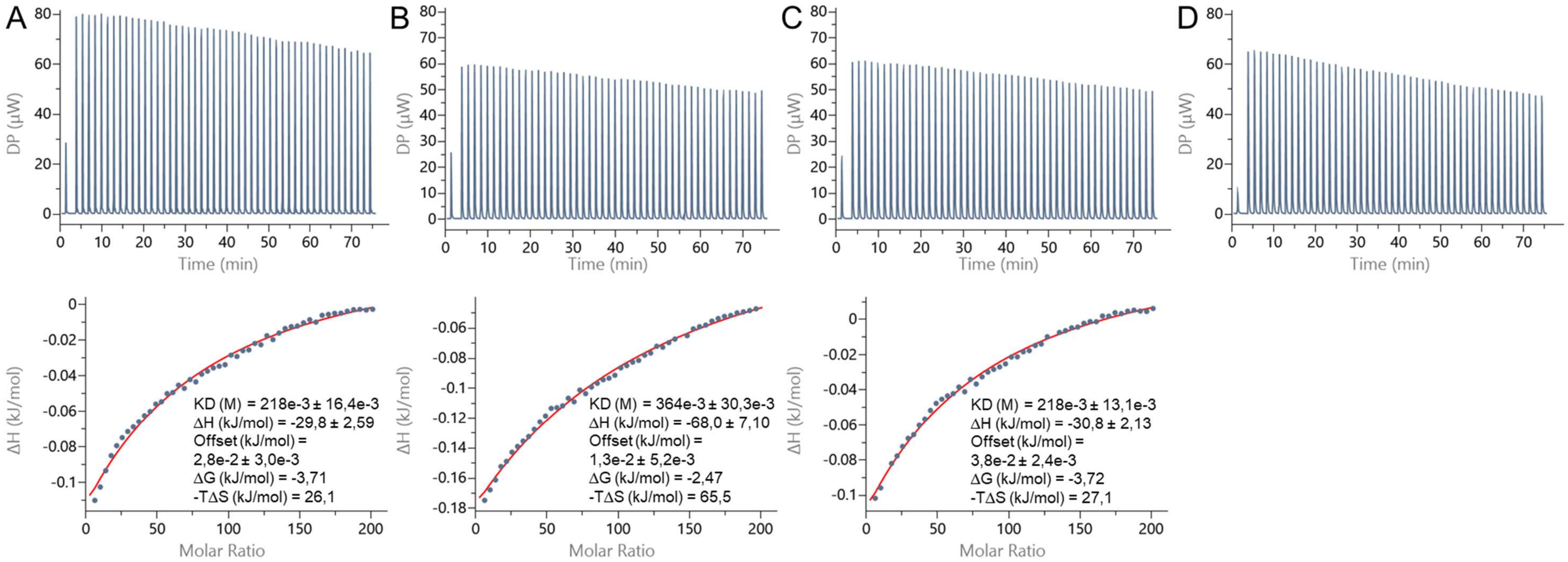

Figure S22. ITC of 1,4-thioxane binding to cycHC[8] in $CH_3OH : H_2O$ 50:50 mixture at 293.35 K (A, B, C) and control experiment (D). *Top:* Data obtained from the sequential injections of 0.8 µL of 1,4-thioxane (0.996 M) to cycHC[8] (1.01 mM) (A, B, C) or to the pure solvent (D) with 90 s spacing. *Bottom*: Plot of the total heat released as a function of total ligand concentration for the titration shown in the upper panel. The red solid line represents least-squares fit of the data. Note: subtracted dilution heat (D) is large due to a high concentration of guest solution.

1,4-dioxane binding to cycHC[8] in CH_3OH

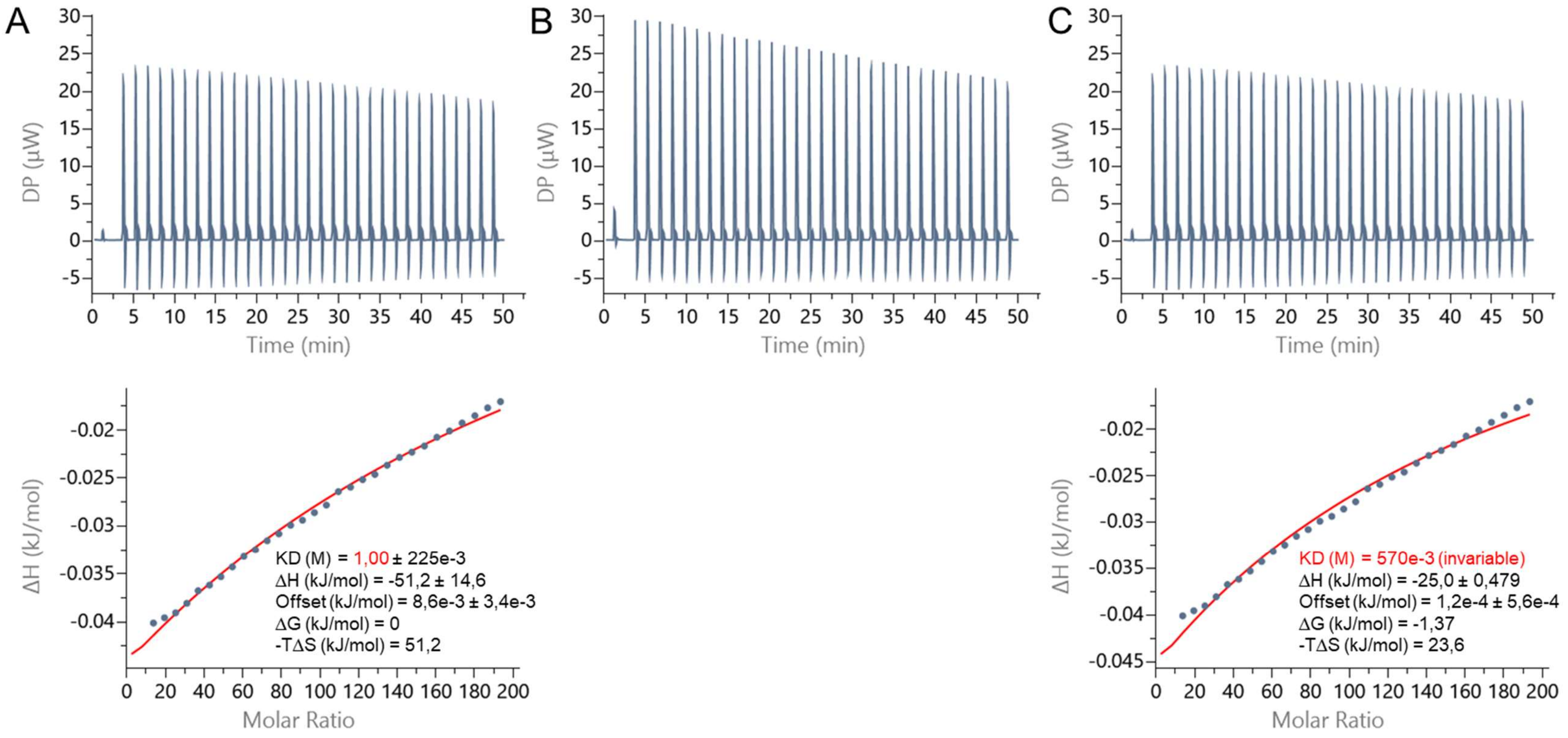

Figure S23. ITC of 1,4-dioxane binding to cycHC[8] in CH_3OH at 293.35 K (A), control experiment (B) and evaluation of same data using association constant obtained from NMR (C). *Top:* Data obtained from the sequential injections of 1.2 µL of 1,4-dioxane (1 M) to cycHC[8] (1.02 mM) (A) or to the pure solvent (B) with 90 s spacing. *Bottom*: Plot of the total heat released as a function of total ligand concentration for the titration shown in the upper panel. The red solid line represents least-squares fit of the data. Note 1: subtracted dilution heat (B) is large due to a high concentration of guest solution. Note 2: ITC data A and C are same titration, only the fitting procedure differs. In first evaluation (A) were simultaneously fitted dissociation constant (K_D), enthalpy (ΔH) and offset as in all experiments shown above. Fitting provided K_D = 1 M, which is highest K_D value allowed in software to be fitted, therefore this value is not precise and indicates that data cannot be fitted. In alternative evaluation (C) we set invariable K_D = 0.57 M corresponding to K_a = 1.75 M^{-1} obtained from NMR titration and we fitted only enthalpy (ΔH) and offset.

Table S8. Summary of ITC results for binding of heterocycles to cycHC[8], only average values. For experimental error see results of particular titrations

**Guest**	**Solvent**	K_a **(M^{-1})**	*ΔH* **(kJ/mol)**	*-TΔS* **(kJ/mol)**
1,3-dithiolane	CH_3OH	13.1	-9.8	3.6
	CH_3OH:H_2O 80:20	20.6	-19.1	11.8
	CH_3OH:H_2O 65:35	60.8	-11.9	1.9
	CH_3OH:H_2O 50:50	65.6	-20.4	10.2
1,4-thioxane	CH_3OH	2.5	-13.7	11.5
	CH_3OH:H_2O 50:50	4.0	-42.9	39.6
1,4-dioxane	CH_3OH	n.d.	n.d.	n.d.

2. Characterization of the sorbents

2.1. Microscopy

Eight bright-field images were acquired for cycHC[6] and cycHC[8] before and after milling. For each sample type, the folder containing the eight TIF format images was imported into the "Images" module. The "EnhanceOrSuppressFeatures" module was implemented to enhance present particles with feature type *Dark holes*. In short, morphological reconstruction was used to identify dark holes within brighter areas and the image was then inverted. Next, the inverted images from module "EnhanceOrSuppressFeatures" were used in the "IdentifyPrimaryObject" module to find objects of interest (cycHC[*n*] particles) in the images. Optimal parameters to find the particles was based on preliminary manual examination in *Test Mode*. Main parameters for all sample types included Threshold strategy set to *Adaptive* with the Thresholding method of *Robust Background*. The Averaging method was set to *Mean* and Variance method to *Standard deviation*. Distinguish clumped objects and drawing lines between clumped objects were both based on the *Shape* of objects. Then the "MeasureObjectSizeShape" module was used to measure different size related features of each found object from the "IdentifyPrimaryObjects" module. A detailed explanation of all the available size measurements can be found in the CP online manual (https://cellprofiler-manual.s3.amazonaws.com/CellProfiler-4.0.4/index.html). Finally, the data was exported as a .csv file to allow further analysis through Microsoft Excel using the "ExportToSpreadsheet" module.

Total number of detected particles for cycHC[8] before milling was 16523 and 26812 after milling. For cycHC[6] before milling, 27335 particles were detected in total and after milling 28837. To asses the ability of CellProfiler's detection, one representative image for cycHC[8] and cycHC[6] respectively (**Figure S24**) was chosen and the number of detected particles by CellProfiler in each image was compared to counting the particles manually by the authors. In the representative image for cycHC[8], CellProfiler detected 2976 particles while manual counting resulted in a slightly higher number of 3032 counted particles. For cycHC[6] the results were similar as CellProfiler detected 3761 particles, while 3838 particles were found by counting. Although, CellProfiler was not able to find all present particles, the method of detection was deemed sufficient for comparing the general size difference of particles between the sample types. Two size measurements, "Area" and average "Feret Diameter", were chosen to compare the size distribution among the samples. Explanation of both size measurements can be found in the CellProfiler online manual by following the above-mentioned link. Microsoft Excel was used to convert measured Area and Minimum and Maxium Feret Diameter of each detected particle by CellProfiler in pixels to square-micrometers (μm^2) and micrometers (µm) respectively. For the Feret diameter, the average of the minimum and maximum values was also taken.

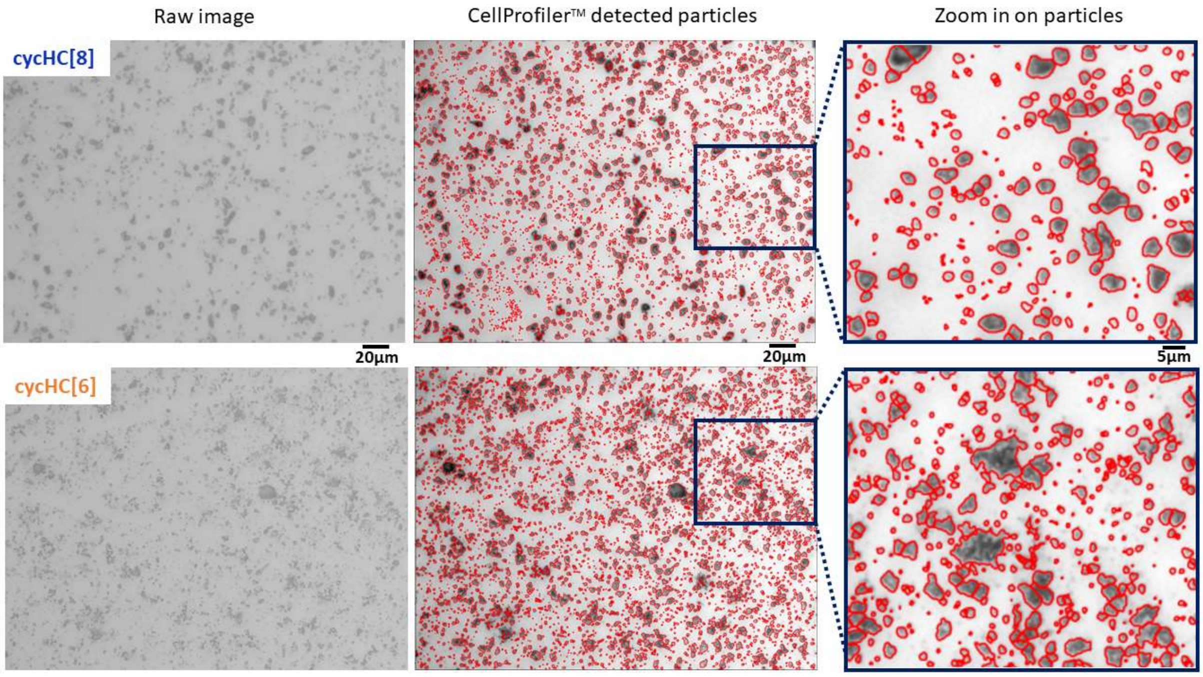

Figure S24. Representative image of cycHC[8] after milling (*top)* and cycHC[6] after milling (*bottom*) respectively. *Left:* Raw image. *Middle:* Image with detected particles (enclosed by red outline) by CellProfiler. *Right:* Zoom for better visualization of detected particles.

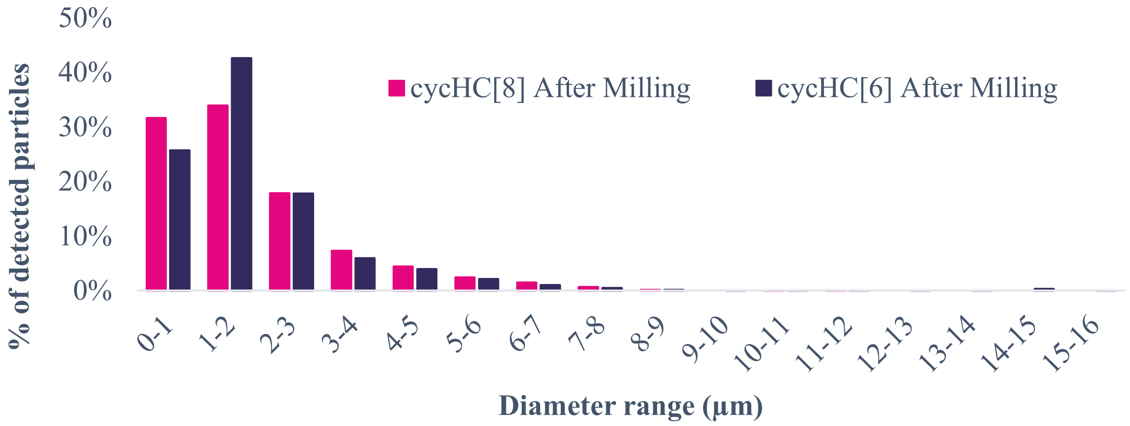

Figure S25. Comparison of percent detected particles in each diameter range (µm) between milled samples. CycHC[8] after milling had slightly higher percent of particles in the 0-1 µm range than cycHC[6] after milling, 32% versus 26% respectively. The roles reversed in the 1- 2 µm range where cycHC[8] after milling had 34% versus cycHC[6] after milling with 43%. In all other ranges the samples had same or next to no differences in their percentages.

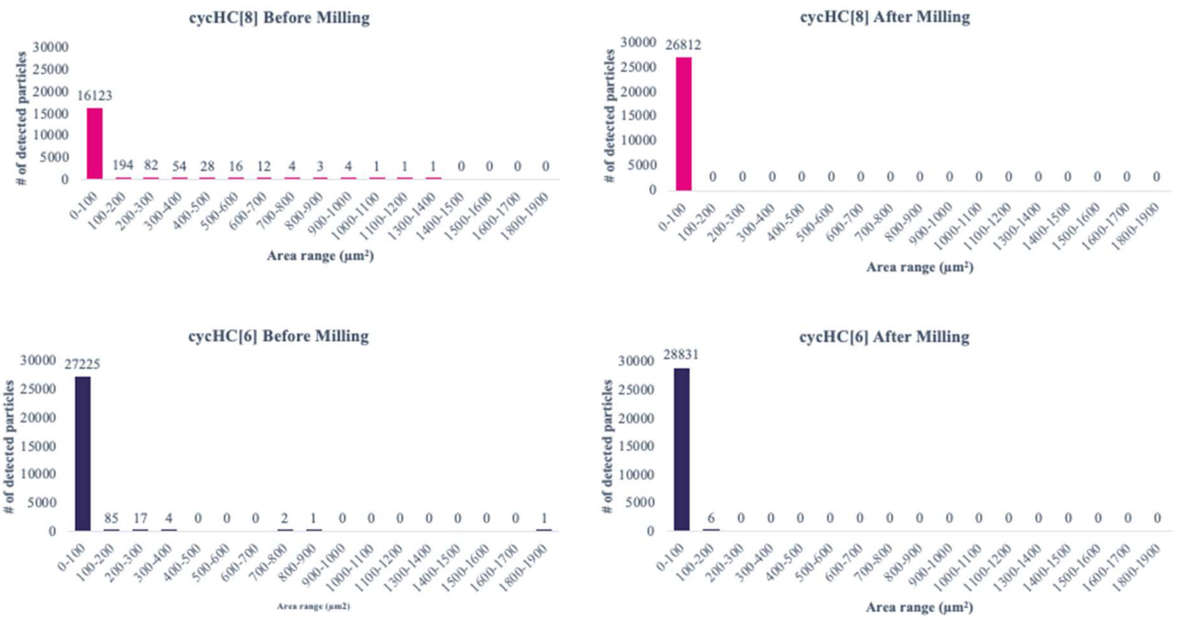

Figure S26. Size distribution depicted as area (µm^2) of cycHC[8] particles before versus after milling (*top*) and cycHC[6] particles before versus after milling (*bottom*). In cycHC[8] before milling there were more than 300 particles with greater area size than 100 µm^2, while there were zero in cycHC[8] after milling. Similarly, there were more than 100 particles with greater size than 100 µm^2 in cycHC[6] before milling and only six after milling. This indicates particle size was reduced in both samples after milling.

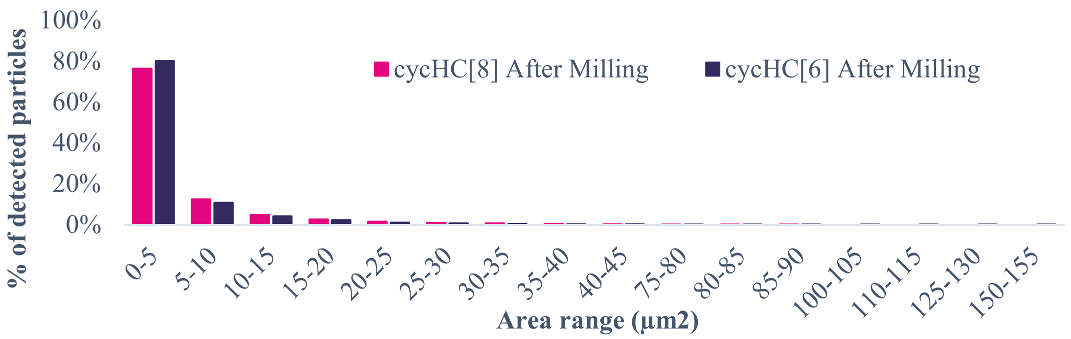

Figure S27. Comparison of percent detected particles in each area range (µm^2) between milled samples. In almost all area ranges, both samples exhibited same or almost no difference in their percentages. The greatest difference was seen in the lowest range of 0- 5 µm^2, where cycHC[8] after milling had 76%, while cycHC[6] after milling had 80%.

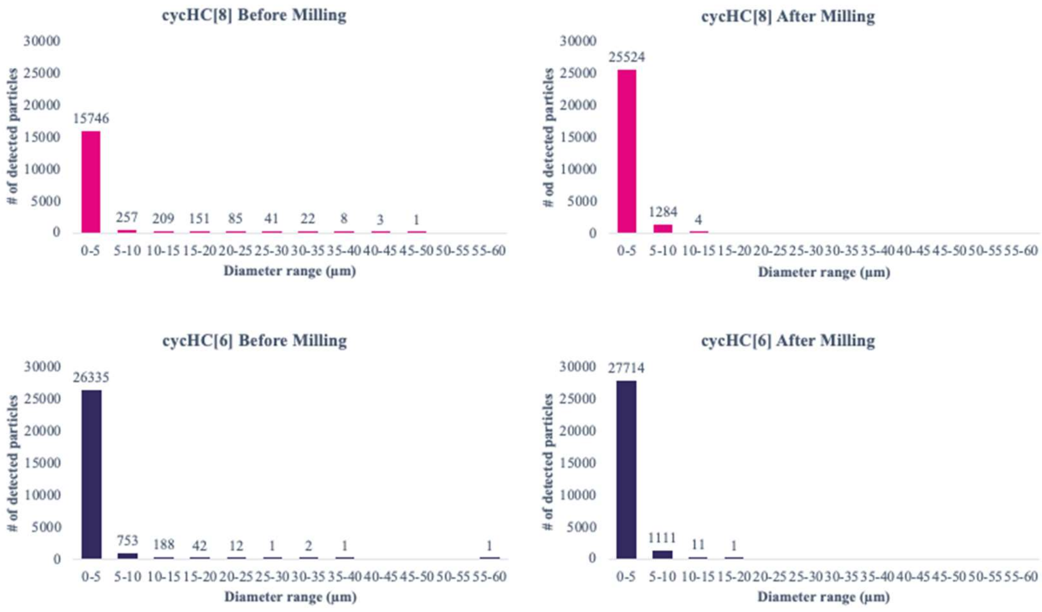

Figure S28. Size distribution depicted as average Feret Diameter (µm) of cycHC[8] particles before versus after milling (*top*) and cycHC[6] particles before versus after milling (*bottom*). In cycHC[8] before milling there were on average around 200 particles in the ranges from 5- 10 µm up to 15-20 µm and there were particles present with diameter up to 45-50 µm. For cycHC[8] after milling there were still many particles (1284) in the 5-10 µm range, but none over 15 µm. This tendency can also be seen with cycHC[6], where before milling there were particles up to 55-60 µm (although only one in this range), while in cycHC[6] before milling there were no particles over 20 µm and even in this range there was only one. This supports the trend seen in the Area measurements, indicating that particle size was indeed reduced in both samples after milling.

2.2. Surface area analysis

Table S9. Summary of surface area analysis for cycHC[8] and cycHC[6]

Surface area	cycHC[8]	cycHC[6]
BET	9.02 m^2/g<br>C: 42.83<br>V_m: 2.054 cc/g	6.03 m^2/g<br>C: 98.6<br>Vm: 1.373 cc/g
Langmuir	12.97 m^2/g<br>C: 18.73<br>V_m: 2.954 cc/g	8.17 m^2/g<br>C: 32.71<br>Vm: 1.862 cc/g

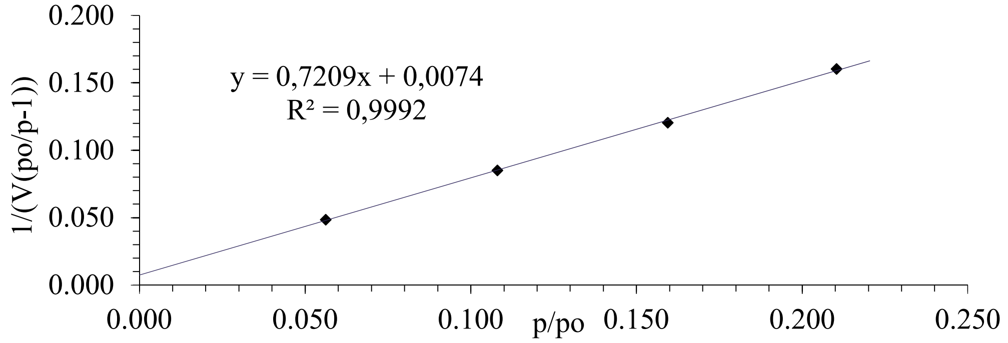

Figure S29. BET plot of N_2 volume absorbed (cc/g) against relative pressure for cycHC[6].

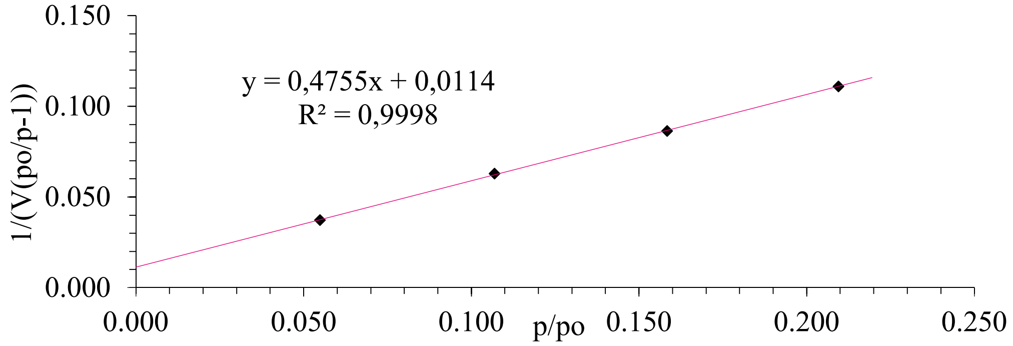

Figure S30. BET plot of N_2 volume absorbed (cc/g) against relative pressure for cycHC[8].

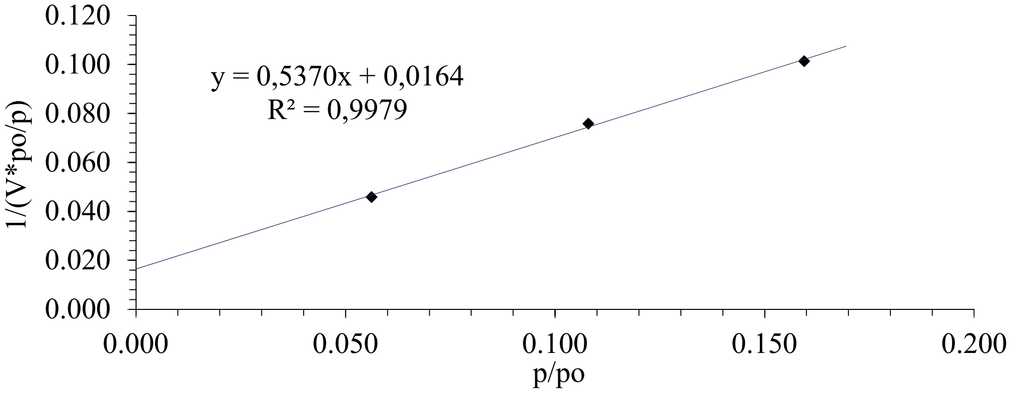

Figure S31. Langmuir plot of N_2 volume absorbed (cc/g) against relative pressure for cycHC[6].

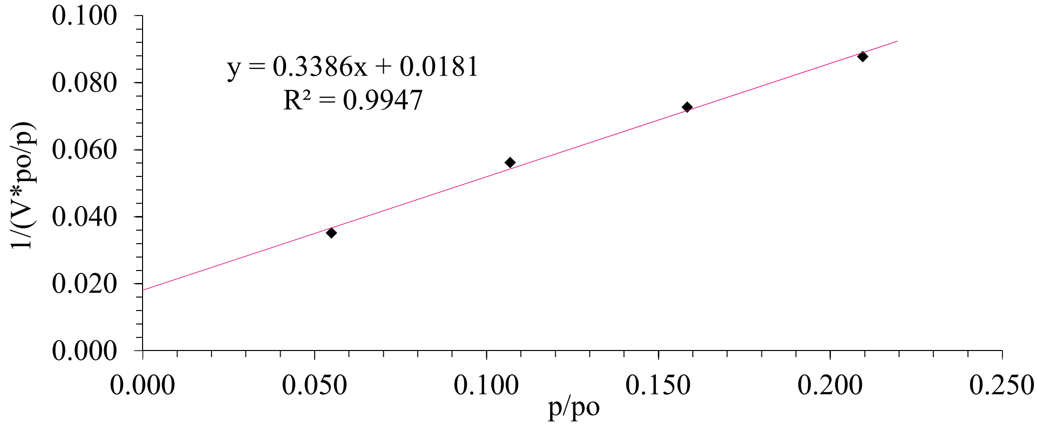

Figure S32. Langmuir plot of N_2 volume absorbed (cc/g) against relative pressure for cycHC[8].

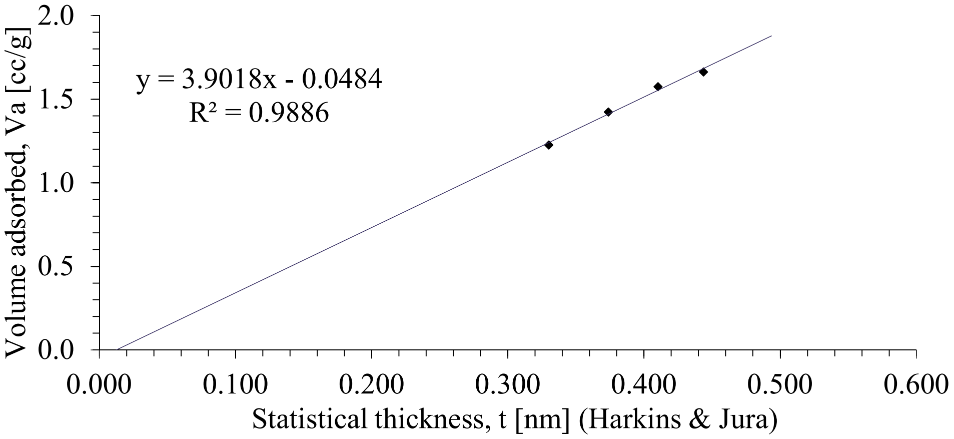

Figure S33. Micropore analysis t-plot of N_2 volume adsorbed against statistical thickness for cycHC[6].

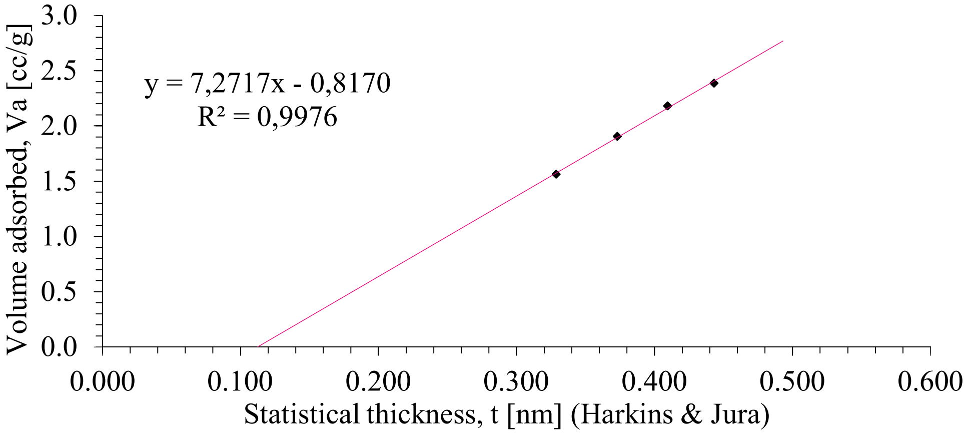

Figure S34. Micropore analysis t-plot of N_2 volume adsorbed against statistical thickness for cycHC[8].

3. Extraction of neutral guests from water

General extraction procedure:

Solid sorbent (macrocyclic host or powdered silicarbon TH90) was dispersed in guest solution and stirred for 30–60 min. The suspension was further separated by either centrifuging or filtration and clear filtrate was analyzed for guest content by HPLC or UV (more details are provided below).

Removal percentage (*%R*) of the guest upon extraction was calculated using formula (1) for HPLC or (2) for UV measurements:

$$\%R = \frac{S_0 - S_i}{S_0} \cdot 100 \quad (1)$$

where S_0 – guest peak area in control solution in the absence of host, mAU·s; S_i – guest peak area in experimental solution (mixed with solid host and centrifuged/filtered), mAU·s;

$$\%R = \frac{A_0 - A_i}{A_0} \cdot 100 \quad (2)$$

where A_0 – guest absorption in control solution in the absence of host, AU, A_i – guest absorption in experimental solution (mixed with solid host and filtered), AU.

Sorption efficiency (*SE*, μg/cm^2), expressed as mass of the sorbed guest per surface area of the respective sorbent, was estimated according to formula (3):

$$SE = \frac{c_0 \cdot V \cdot M \cdot \%R \cdot 1000}{100 \cdot m_i \cdot s_i} \quad (3)$$

where c_0 – initial concentration of the guest in the solution, mM; V – volume of the guest solution used in the extraction, l; M – guest molar weight, g/mol; $\%R$ – percentage of the guest removed during extraction, %; m_i – weight of the sorbent used in the experiment, g; s_i – sorbent surface area, cm^2/g.

Analytical procedures were developed ahead of the extraction experiments, and their performance was checked to provide reliable quantitative data. Method linearity and LoD were studied on a series of guest compound aqueous solutions of known concentrations, prepared by consequent dilution of stock solution in two parallels for each analyte (from two independent stock solutions). Concentration range was chosen according to the experimental planning and the lowest concentrations corresponding to LoD of the proposed method. Method linearity was evalutated based on linear regression and LINEST analysis. LoD was determined as S/N ≈ 3. To study the possible effect of analyte sorption on membrane material during filtration, test solution of an analyte was analyzed before and after filtration, and the difference between obtained peak areas / UV absorption was expressed as percentage.

3.1. Quantitative analysis of S- and O-containing heterocycles by HPLC-UV method

General conditions for HPLC analysis of 1,4-dioxane, 1,4-dithiolane and 1,3-dithiolane

HPLC analysis was performed on Macherey-Nagel Nucleoshell RP18 column (150 mm × 3.0 mm, 2.7 µm). Mobile phase comprised of H_2O : CH_3CN 55:45 (v/v) in isocratic mode with the flow rate of 0.2 mL/min. The column temperature was set at 30 °C, injection volume at 5 µl and detection wavelength at 200 nm. The conditions were developed based on a procedure described in literature (Lees et al., 2017).

Analytical method characteristics

Method performance was studied on a series of analyte aqueous solutions:

1,4-dioxane. 9.1 mM, 1.8 mM, 0.37 mM, 0.07 mM, 0.02 mM.

1,4-thioxane. 3.9 mM, 0.04 mM, 0.004 mM, 0.001 mM, 0.0007 mM.

1,3-dithiolane. 3.4 mM, 1.0 mM, 0.1 mM, 0.01 mM, 0.001 mM, 0.0003 mM.

Table S10. Analytical method characteristics

Analyte	Linear regression	R^2	LoD, mM
1,4-dioxane	y=(69.6±0.1)x-(0.5±0.2)	1.0000	0.0240
1,4-thioxane	y=(3412±7)x+(10±11)	1.0000	0.0007
1,3-dithiolane	y=(5230±37)x+(11±53)	0.9995	0.0003

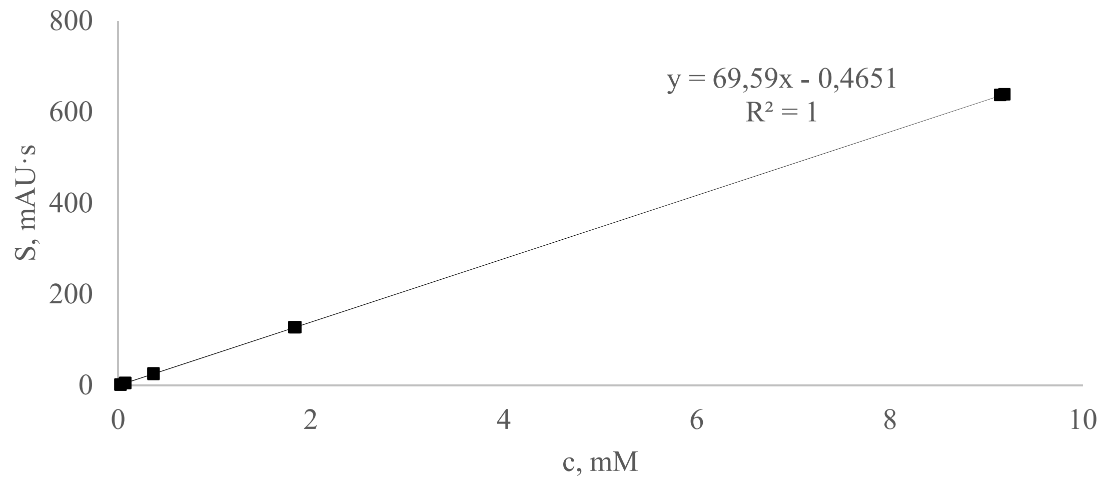

Figure S35. Linear dependency between peak area and 1,4-dioxane concentration in the solution.

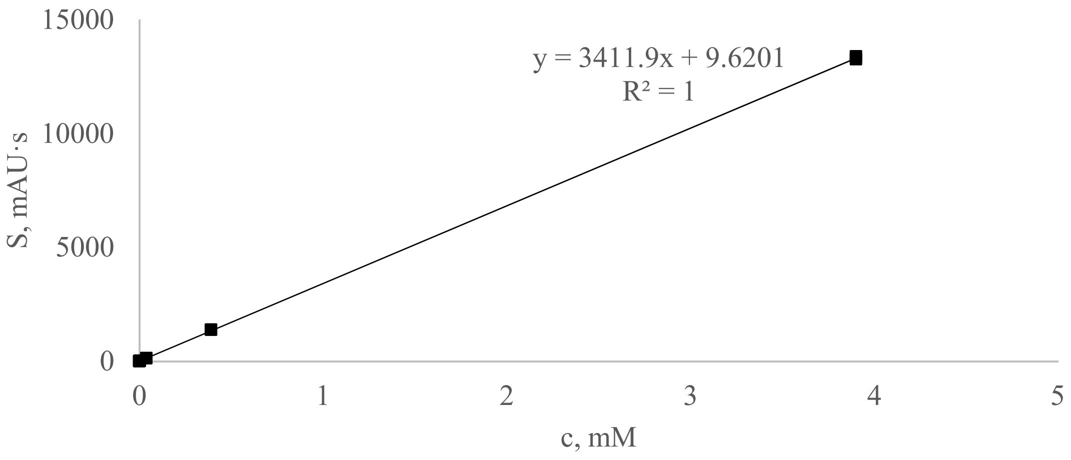

Figure S36. Linear dependency between peak area and 1,4-thioxane concentration in the solution.

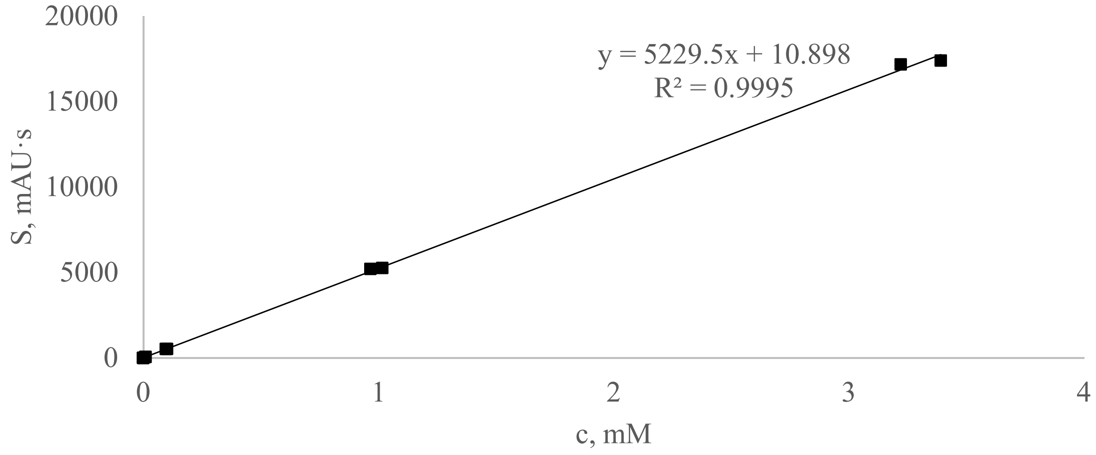

Figure S37. Linear dependency between peak area and 1,3-dithiolane concentration in the solution.

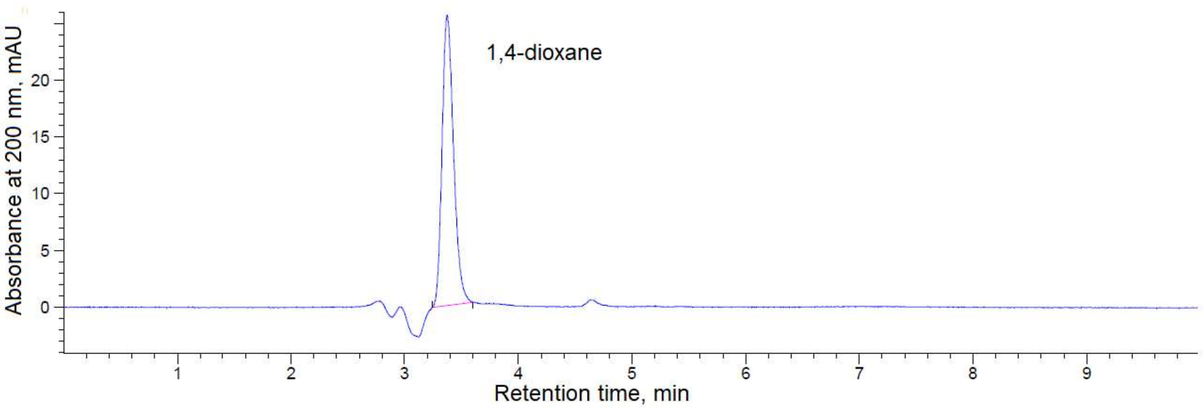

Figure S38. Typical chromatogram of 1,4-dioxane solution.

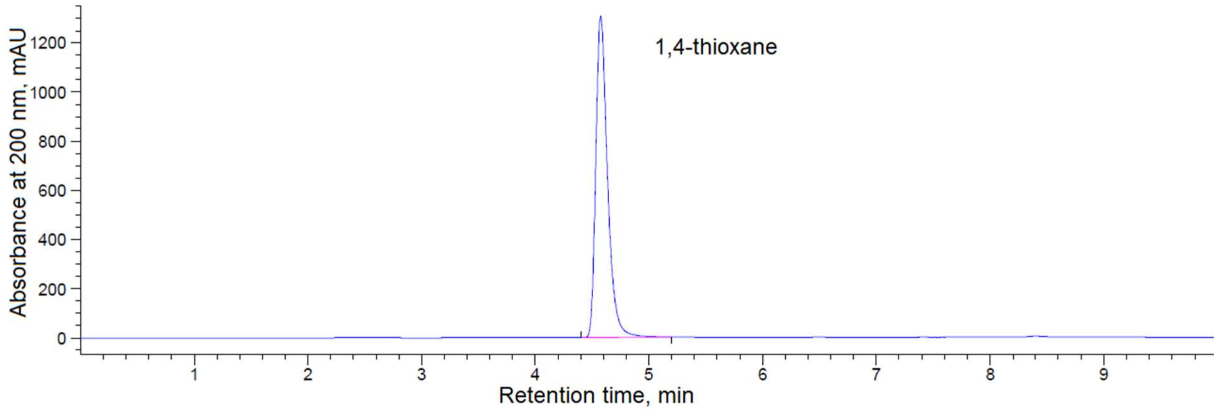

Figure S39. Typical chromatogram of 1,4-thioxane solution.

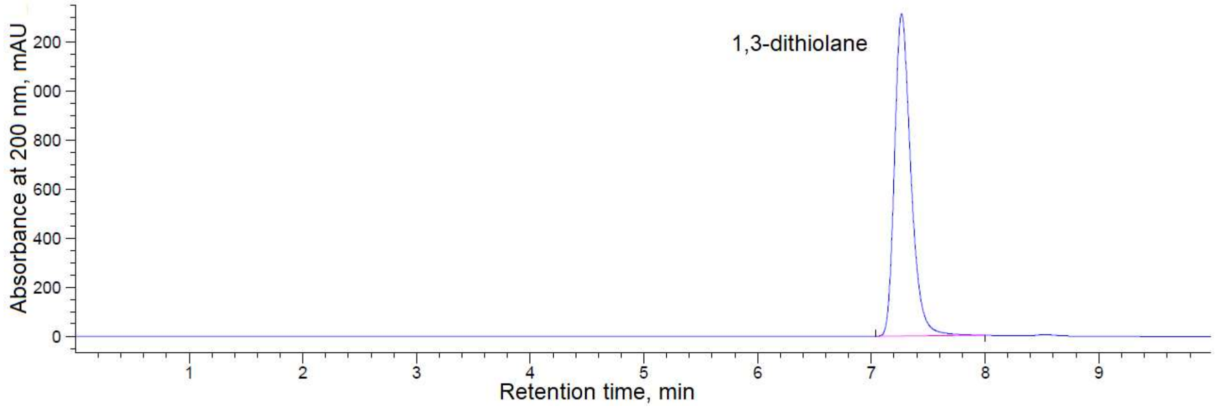

Figure S40. Typical chromatogram of 1,3-dithiolane solution.

***General extraction procedure and sample preparation*:**

Stock solution of guest compound. ~2.7 mM of guest compound in H_2O for experiments with cycHC[6]. ~2.2 mM of guest compound in H2O for experiments with cycHC[8].

Blank suspension (x 1) of host compound. ~100 mg cycHC[n] or silicarbon and 8 ml H_2O.

Control solution (x 1). 8 ml of stock solution.

Experimental suspension (x 3). ~100 mg cycHC[*n*] or silicarbon TH90 and 8 ml of stock solution.

Control solution and blank and experimental suspensions were each placed into a 15 ml centrifuge tube and mixed on Vortex-Genie 2 mixer for 30 min, followed by centrifuging at 9000 rpm for 30 min. From each, 500 µl of clear supernatant (experimental solution) was collected and analyzed by HPLC method described above.

General conditions for HPLC analysis of α-Lipoic Acid

HPLC analysis was performed on Phenomenex Kinetex XB-C18 column (150 mm × 4.6 mm, 2.6 µm). A – H_2O : HCOOH (100:0.1 v/v) and B – CH_3CN : HCOOH (100:0.1 v/v) were used as eluents, A : B 50 : 50 (v/v) in isocratic mode with the flow rate of 0.5 mL/min. The column temperature was set at 30 °C, injection volume at 5 µl and detection wavelength at 210 nm.

Analytical method characteristics

Method performance was checked on a series of α-lipoic acid aqueous solutions: 412 µM, 82.4 µM, 41.2 µM, 20.6 µM, 10.3 µM.

Table S11. Analytical method characteristics

Parameter	Result
Linearity: equation of linear regression, R^2	y=(1.068±0.007)x+(1.2±0.6), R^2=0.9999
LoD, µM	10
Effect of filtration: ΔS, %	0.5

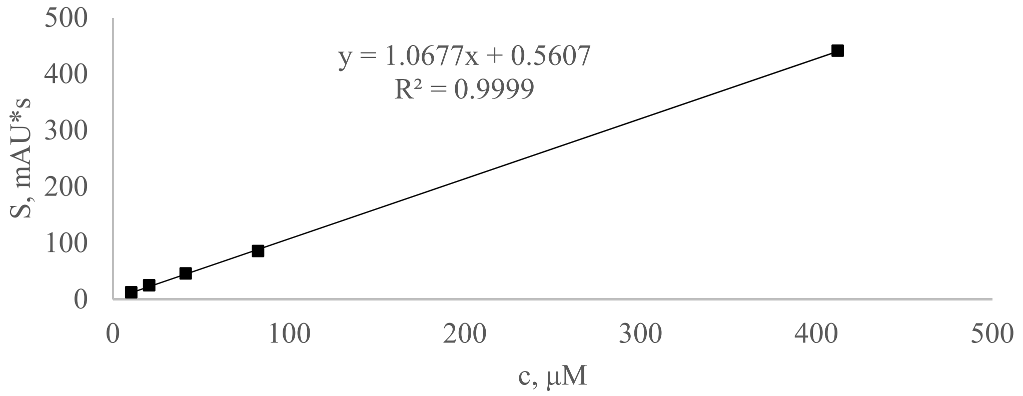

Figure S41. Linear dependency between peak area and α-lipoic acid concentration in the solution.

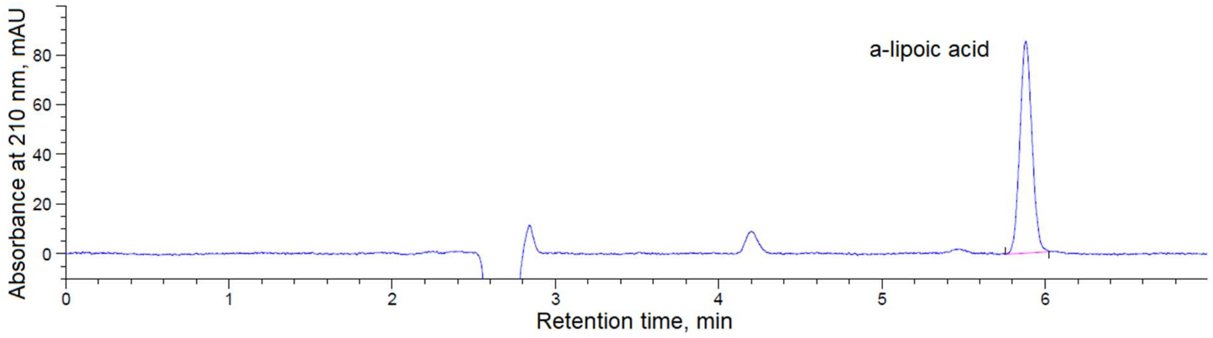

Figure S42. Typical chromatogram of α-lipoic acid solution.

***General extraction procedure*:**

Stock solution of guest compound. ~0.4 mM of α-lipoic acid in H_2O.

Blank suspension (x 1) of host compound. ~16 mg cycHC[6], or ~20 mg of cycHC[8], or ~20 mg silicarbon TH90 and 8 ml H_2O.

Control solution (x 2). 8 ml of stock solution.

Experimental suspension (x 2). ~16 mg cycHC[6] (H:G 5:1), or ~20 mg cycHC[8] (H:G 5:1), or ~20 mg silicarbon TH90, or ~100 mg cycHC[8] (H:G 20:1) and 8 ml of stock solution.

Control solution and blank and experimental suspensions were each placed into a 20 ml vial and mixed on a Stuart magnetic stirrer at 500 rpm for 1 hr. Then the samples were filtered through Phenex-RC 4 mm syringe filter, 0.2 µm, and clear filtrate (experimental solution) was subjected to HPLC analysis.

3.2. Quantitative analysis of N-containing heterocycles by UV spectrophotometry

General conditions for UV spectrophotometric analysis

UV spectra were measured in a 10 mm quartz cuvette. From each collected spectrum the respective solvent baseline was subtracted. The absorption of imidazole and 1-methylimidazole was measured at 205 and 209 nm, respectively.

Analytical method characteristics

Method linearity was investigated based on absorption of a series of imidazole aqueous solutions of known concentrations, prepared by consequent dilution of stock solution:

Imidazole. 206 µM, 103 µM, 51.4 µM, 25.7 µM, 12.9 µM.

1-methylimidazole. 206 µM, 103 µM, 51.4 µM, 25.7 µM, 12.9 µM.

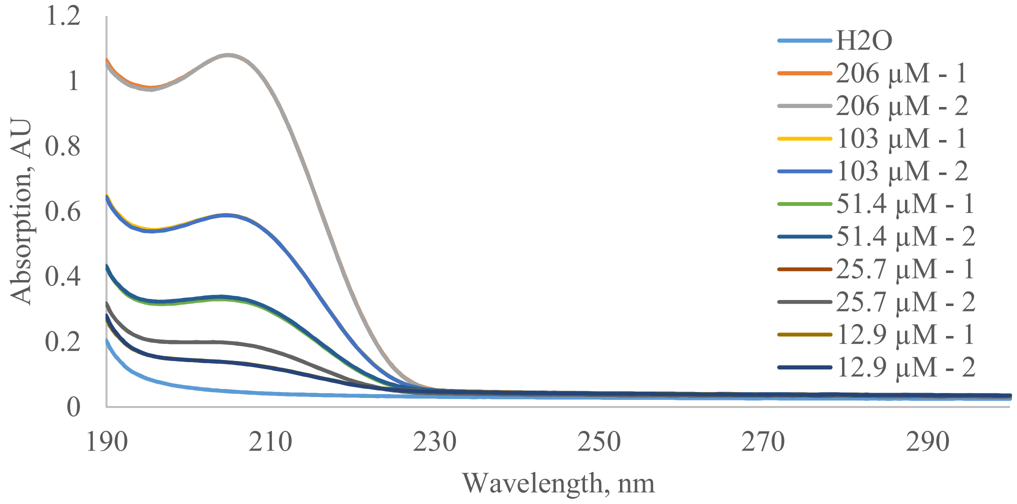

Figure S43. Linearity studies for imidazole determination.

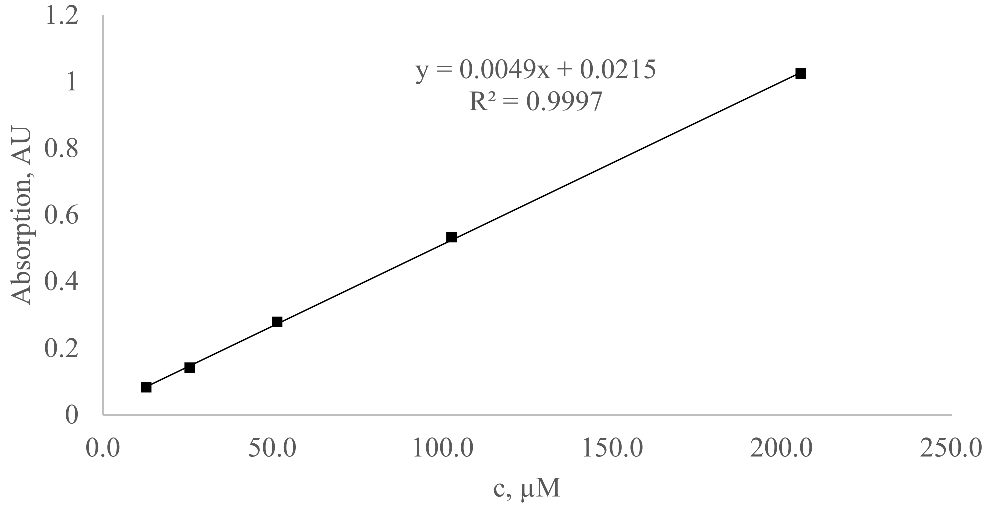

Figure S44. Linear dependency between absorption and imidazole concentration in solution.

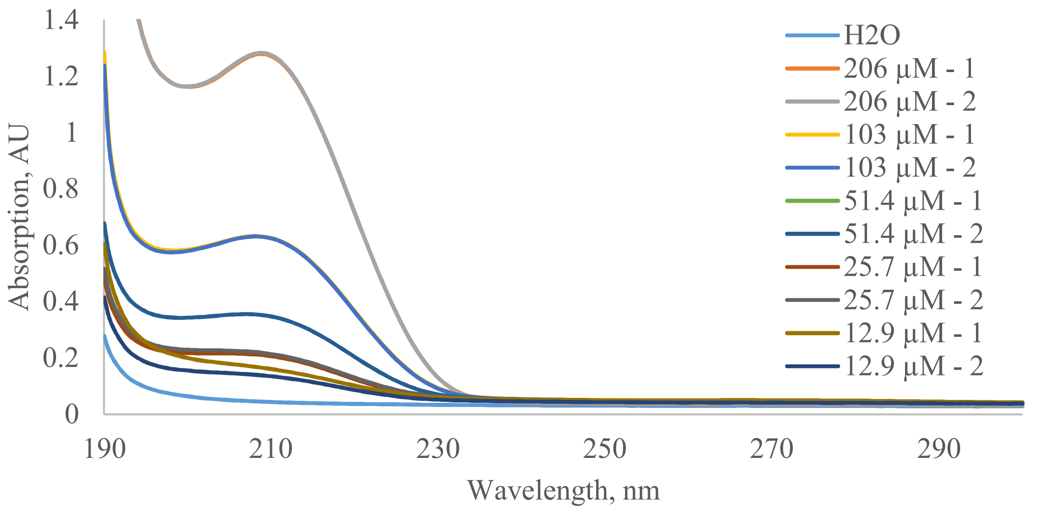

Figure S45. Linearity studies for 1-methylimidazole determination.

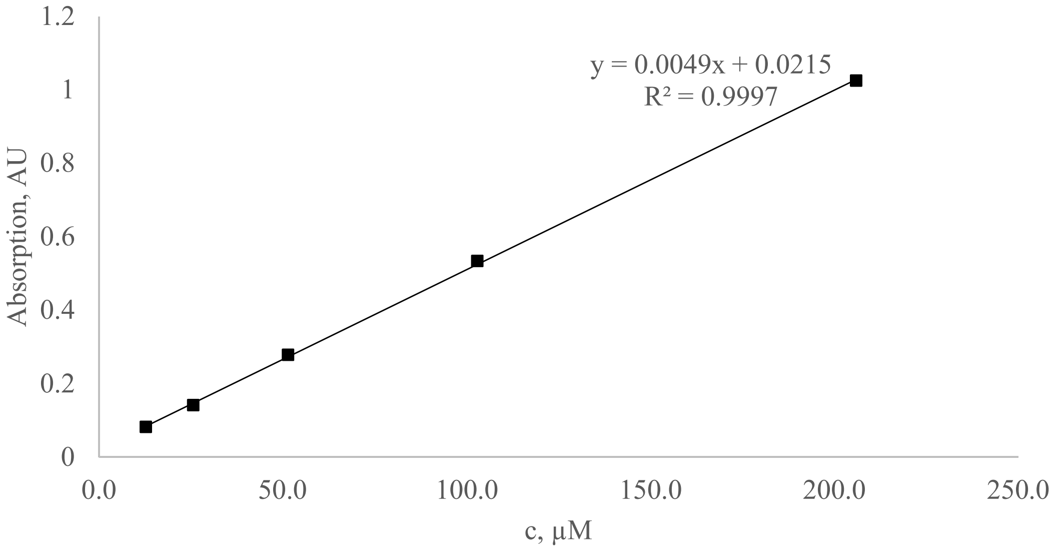

Figure S46. Linear dependency between absorption and 1-methylimidazole concentration in solution.

To study the possible effect of analyte sorption on filter (Chromafil ® Xtra H-PTFE 45/25, 0.45 µm), 103 µM analyte solution was measured before and after filtration, and the difference between obtained absorption values (ΔA) was expressed as percentage.

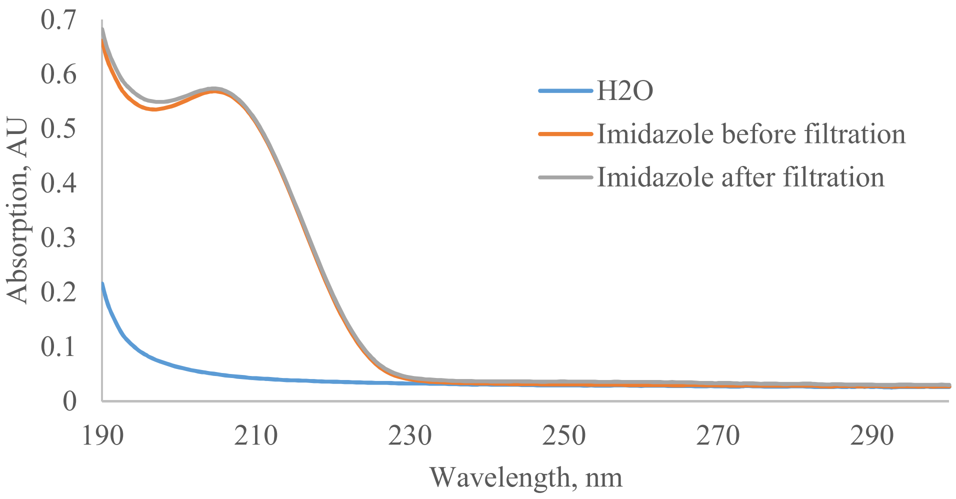

Figure S47. Filtration effect on imidazole concentration in solution.

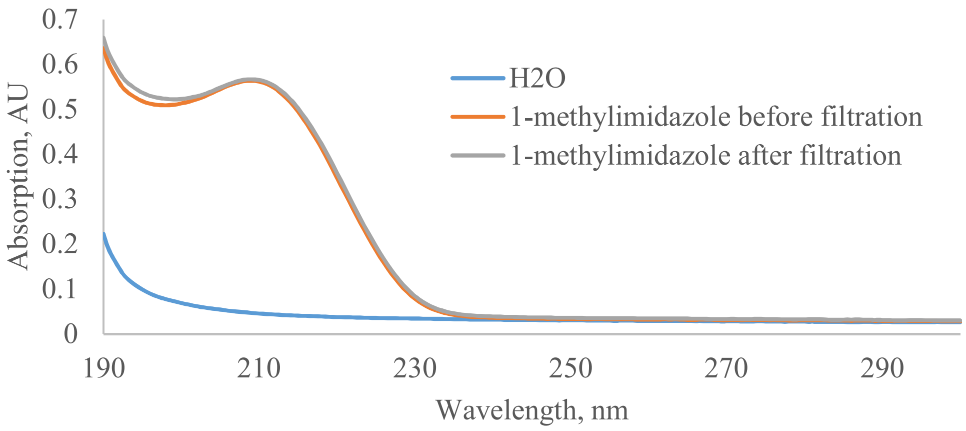

Figure S48. Filtration effect on 1-methylimidazole concentration in solution.

Table S12. Analytical method characteristics

**Analyte**	**Linear regression**	R^2	ΔA, %
Imidazole	y=(0.00490±0.00005)x+(0.0225±0.005)	0.9997	1.0
1-methylimidazole	y=(0.00490±0.00005)x+(0.0225±0.005)	0.9997	0.7

***General extraction procedure*:**

Stock solution of guest compound. 0.4 mM imidazole or 1-methylimidazole in H_2O.

Blank suspension (x 1) of host compound. ~16 mg cycHC[6] or ~20 mg cycHC[8], or ~20 mg silicarbon TH90 and 8 ml of H_2O.

Control solution (x 2). 8 ml of stock solution.

Experimental suspension (x 2). ~16 mg cycHC[6], or ~20 mg cycHC[8], or ~20 mg silicarbon TH90 and 8 ml of stock solution.

Control solution and blank and experimental suspensions were each placed into a 20 ml vial and mixed on a Stuart magnetic stirrer at 500 rpm for 1 hr. The samples were then filtered

through Chromafil ® Xtra H-PTFE 45/25, 0.45 µm syringe filter. 2.5 ml of the filtrate was diluted with H_2O to 10.0 ml, thoroughly mixed and the UV absorption of the obtained solution (experimental solution) was measured. The results revealed that none of the N-containing heterocycles concentrations significantly decreased upon extraction.

Table S13. Absorption values measured after extraction of N-heterocycles by cycHC[*n*]

**Solution**	***A*, AU**	***A*$_{solvent}$, AU**	***A*$_i$, AU**	***A*$_0$, AU**
				**Imidazole (at 205 nm)**
H_2O		0.06		
cycHC[6]	0.12		0.06	
cycHC[8]	0.18		0.12	
Stock	0.62		0.56	
Control-1	0.63		0.57	0.57
Control-2	0.63		0.57	
Exp_cycHC[6]-1	0.62		0.56	*No significant change compared to control soulution.*
Exp_cycHC[6]-2	0.62		0.56	
Exp_cycHC[8]-1	0.73		0.67	*Increase in absorption (due to relatively higher solubility of cycHC[8] in H_2O)*
Exp_cycHC[8]-2	0.73		0.67	
				**1-methylimidazole (at 209 nm)**
H_2O		0.17		
cycHC[6]	0.20		0.04	
cycHC[8]	0.25		0.09	
Stock	0.67		0.51	
Control-1	0.70		0.53	0.52
Control-2	0.68		0.51	
Exp_cycHC[6]-1	0.67		0.51	*No significant change compared to control soulution.*
Exp_cycHC[6]-2	0.67		0.51	
Exp_cycHC[8]-1	0.78		0.62	*Increase in absorption (due to relatively higher solubility of cycHC[8] in H_2O)*
Exp_cycHC[8]-2	0.80		0.64	

Table S14. Absorption values measured after extraction of N-heterocycles by silicarbon TH90

**Solution**	***A*, AU**	***A*$_{solvent}$, AU**	***A*$_i$, AU**	***A*$_0$, AU**	***%R***	<***%R***>[a], **%**
						**Imidazole (at 205 nm)**
H_2O		0.06				
Blank	0.07		0.01			
Control-1	0.57		0.51	0.51		
Control-2	0.57		0.51			
Exp-1	0.29		0.23		56.1	56.3±0.3
Exp-2	0.28		0.22		56.4	
						**1-methylimidazole (at 209 nm)**
H_2O		0.17				
Blank						
Control-1						
Control-2						
Exp-1				0.52	71.9	71.7±0.3
Exp-2					71.5	

[a] The average SE value is provided as mean value ± standard deviation between parallel experiments (n=2)

3.3. Extraction results

Table S15. Extraction of neutral guests from water by 5 molar excess of cycHC[*n*] and equivalent weight of silicarbon TH90

**Guest**	**Host (sorbent)**	**Host (sorbent), mg**	c_{guest}, **mM**	***%R***	***<%R>***[a]	***SE*, μg/cm²**	***log*(*SE*)**
1,4-dioxane **6**	cycHC[6]	100	2.7	0.5<br>1.1<br>0.2	0.6±0.4	19	1.3
	cycHC[8]	100	2.2	6.6<br>4.1<br>4.7	5±1	86	1.9
	TH90	100	2.2	63<br>67<br>70	67±4	10	1.0
1,4-thioxane **3**	cycHC[6]	100	2.7	2.6<br>3.9<br>3.3	3±1	112	2.0
	cycHC[8]	100	2.2	24.8<br>24.7<br>25.2	24.9±0.2	506	2.7
	TH90	100	2.2	97.9<br>97.8	97.8±0.1	18	1.3
1,3-dithiolane **1**	cycHC[6]	100	2.7	15.3<br>17.6<br>15.8	16±1	609	2.8
	cycHC[8]	100	2.2	78.9<br>78.6<br>77.7	78±1	1616	3.2
	TH90	100	2.2	98.7<br>98.5<br>97.8	98.3±0.5	18	1.3
α-lipoic acid (racemic) **9**	cycHC[6]	20	0.4	46.3<br>45.7	46.0±0.4	2518	3.4
	cycHC[8]	20	0.4	74.2<br>72.8	74±1	2708	3.4
	TH90	20	0.4	97.8<br>97.3	97.5±0.4	32	1.5
Imidazole **11**	cycHC[6]	16	0.4	–	–	–	–
	cycHC[8]	20	0.4	–	–	–	–
	TH90	20	0.4	56.1<br>56.4	56.3±0.3	9	0.8

Continuation of Table S14

Guest	Host (sorbent)	Host (sorbent), mg	c_{guest}, mM	*%R*	<*%R*>[a]	*SE*, μg/cm^2	*log*(*SE*)
1-methyl-imidazole **10**	cycHC[6]	16	0.4	–	–	–	–
	cycHC[8]	20	0.4	–	–	–	–
	TH90	20	0.4	71.9<br>71.5	71.7±0.3	9	1.0

[a] The average *%R* value is provided as mean value ± standard deviation between parallel experiments (n≥2)

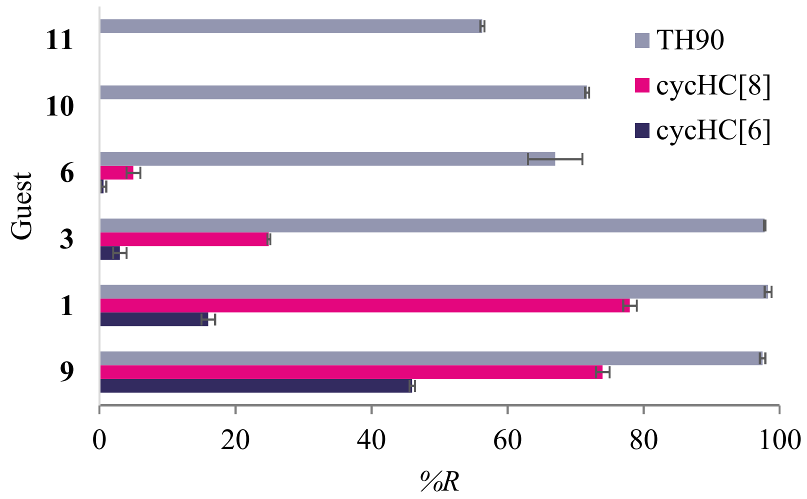

Figure S49. Removal percentage during extraction of various heterocyclic guests from their individual solutions by different sorbents. The error bars represent standard deviation between parallel experiments (n≥2).

Table S16. Removal percentage of enantiomerically pure α-lipoic acid extracted by 20 molar excess of cycHC[8]

Guest/Host	(*R*,*R*)-cycHC[8]	(*S*,*S*)-cycHC[8]
(*R*)-(+)-α-lipoic acid	96	93
(*S*)-(-)-α-lipoic acid	95	91

Table S17. Octanol/water partition coefficients for different guest compounds (PubChem [Internet]. Bethesda (MD): National Library of Medicine (US), National Center for Biotechnology Information; 2004-.)

Guest	*LogP*
1,4-dioxane	-0.27[a]
Imidazole	-0.08[a]
1-methylimidazole	-0.06[a]
1,4-thioxane	0.50[b]
1,3-dithiolane	1.40[b]
α-Lipoic acid	2.10[a]

[a] Experimental value. [b] Computed automatically by XLogP3 3.0 from the given chemical structure.

3.4. Selectivity during extraction

Selectivity of cycHC[8] was studied in comparison to a standard activated carbon adsorbent – silicarbon TH90 special, Aktivkohle.

Prior to selectivity experiment, removal efficiency of silicarbon TH90 toward 1,3-dithiolane was investigated in the range of 1.2 – 18 mg per one extraction round, using the following procedure:

Stock solution of guest mixture. ~ 2 mM 1,3-dithiolane solution in H_2O.

Control solution (x 1). 8 ml of stock solution.

Experimental suspension (x 2). 1.2 – 18 mg silicarbon TH90 and 8 ml of stock solution.

Control solution and blank and experimental suspensions were each placed into a 10 ml capped vial and mixed on a Stuart magnetic stirrer at 500 rpm for 30 min. The samples were then filtered through Chromafil ® Xtra H-PTFE 45/25, 0.45 µm syringe filter, clear filtrate was collected and analyzed by HPLC method described above.

Table S18. Removal percentage provided by silicarbon TH90 depending on the used amount

Guest	Sorbent	$m_{sorbent}$, mg	$\%R_1$	$\%R_2$	<%R >[a]
1,3-dithiolane **1** 2mM	TH90	1.2	20.1	21.0	20.5±0.6
		6.0	46.1	49.1	48±2
		12.0	68.6	70.3	70±1
		15.0	78.7	78.8	78.7±0.1
		16.0	79.4	79.3	79.4±0.1
		17.0	80.6	80.9	80.8±0.2
		18.0	81.5	82.0	81.8±0.3

[a] The average *%R* value is provided as mean value ± standard deviation between parallel experiments (n=2)

The obtained results showed that **15 mg** of silicarbon TH90 exhibits removal percentage similar to that of 100 mg cycHC[8] during extraction of 1,3-dithiolane from its 2 mM solution.

***General procedure for selectivity studies*:**

Stock solution of guest mixture. 1,3-dithiolane (~0.7 mM), 1,4-thioxane (~0.7 mM), 1,4- dioxane (~0.7 mM) solution in H_2O.

Control solution (x 1). 8 ml of stock solution.

Experimental suspension (x 2). ~100 mg cycHC[8] or ~15 mg silicarbon TH90 or ~100 mg silicarbon TH90 and 8 ml of stock solution.

Control solution and blank and experimental suspensions were each placed into a 10 ml capped vial and mixed on a Stuart magnetic stirrer at 500 rpm for 30 min. The samples were then filtered through Chromafil ® Xtra H-PTFE 45/25, 0.45 µm syringe filter, clear filtrate was collected and analyzed by HPLC method described above.

Table S19. Extraction of different S– and O–heterocycles from their mixture by cycHC[8] and silicarbon TH90

Guest	100 mg cycHC[8]		15 mg TH90		100 mg silicarbon TH90	
	%R	**<*%R*>[a]**	***%R***	**<*%R*>[a]**	***%R***	**<*%R*>[a]**
1,3-dithiolane **1**	83.1<br>83.0	83.0±0.1	81.5<br>81.3	81.4±0.1	98.7<br>98.7	98.66±0.01
1,4-thioxane **3**	23.1<br>24.1	23.6±0.7	54.5<br>54.3	54.4±0.1	95.9<br>96.0	95.9±0.1
1,4-dioxane **6**	1.0<br>0.5	0.8±0.3	6.6<br>9.4	8±2	57.8<br>58.1	57.9±0.2

[a] The average *%R* value is provided as mean value ± standard deviation between parallel experiments (n=2)

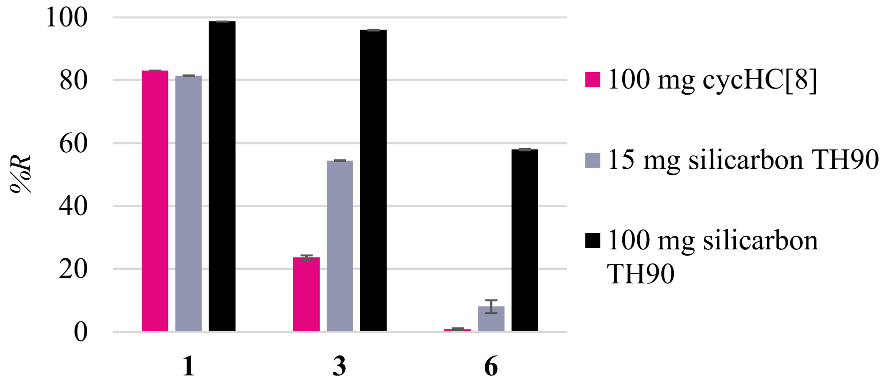

Figure S50. Selectivity of cycHC[8] and silicarbon TH90 toward different S– and O–heterocycles. The error bars represent standard deviation between parallel experiments.

Estimation of selectivity factors

The selectivity factors (*SF*) of cycHC[8] and silicarbon TH90 towards different guests were assessed for pairs 1,3-dithiolane / 1,4-thioxane and 1,3-dithiolane / 1,4-dioxane using formula (4):

$$SF = \frac{\%R_i}{\%R_j} \tag{4}$$

where $\%R_i$ – removal percentage exhibited by the sorbent towards more hydrophobic guest, %; $\%R_j$ – removal percentage efficiency exhibited by the sorbent towards less hydrophobic guest, %.

Table S20. Selectivity factors calculated based on extraction of different guests from their mixture

Sorbent	$SF_{1,3\text{-dithiolane}/1,4\text{-thioxane}}$	$SF_{1,3\text{-dithiolane}/1,4\text{-dioxane}}$
cycHC[8]	3.5	110
TH90	1.5	10

4. Termogravimentric analysis of cycHCs before and after extraction of 1,3-dithiolane

The macrocycles after surface area analysis (2.2) were used to evaluate their thermal stability. No significant mass loss upon heating up to 200 °C was observed. Additionally, decomposition profile of pure 1,3-dithiolane was obtained. For the investigation of host–guest binding, solid macrocyclic cycHC[*n*], which retained guest molecules after the extraction procedure, were analyzed in parallel with pure 1,3-dithiolane. The formation of characteristic mass-fragments of 1,3-dithiolane with *m/z* 47, corresponding to $[CH_2SH]^-$ was followed.

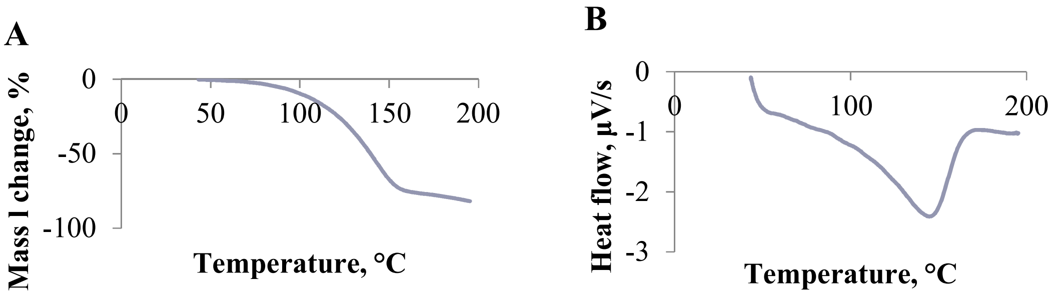

Figure S51. TGA (**A**) and DTA (**B**) of 1,3-dithiolane.

For further analysis, extraction experiment with 1,3-dithiolane, analogous to 3, was performed. After centrifugation stage, the solid material containing cycHC[*n*] complex with 1,3-dithiolane was filtered out and dried under vacuum for 3 hours.

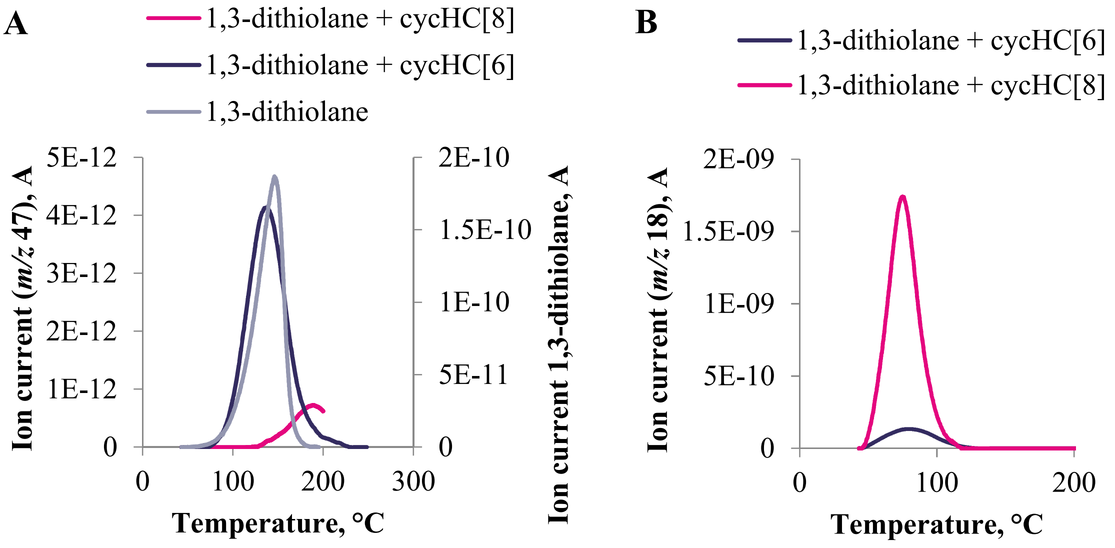

Figure S52. EGA-MS of 1,3-dithiolane (5.8 mg) and its complexes with cycHC[6] (28 mg) and cycHC[8] (27.5 mg): evolution of $[-CH_2SH]^-$ decomposition product of 1,3-dithiolane (*m/z* 47, **A**) and H_2O (*m/z* 18, **B**), latter proves no discrimination of water evaporation between cycHC[6] and cycHC[8].

5. ^{13}C solid-state NMR studies of 1,3-dithiolane and α-lipoic acid interaction with cycHCs

Sample preparation:

cycHC[n]. 50 mg of solid cycHC[*n*] was stirred in H_2O for 1 hour (analogous to control extraction experiment). Then the solid material was filtered out and dried on the filter.

Guest + cycHC[n] mixed. Guest and cycHC[*n*] taken in 1:1 molar ratio were separately grinded in a ceramic mortar with a ceramic pestle, and manually mixed together in a vial.

Guest + cycHC[n] milled. Guest and cycHC[*n*] taken in 1:1 molar ratio with H_2O taken in liquid assited grinding amount (η=0.3 µl/mg) were milled together in a 14 mL ZrO_2-coated grinding jar charged with 3 x 7 mm ZrO_2 milling balls at 30 Hz for 10 min.

Lipoic acid. Pure solid compound (racemic).

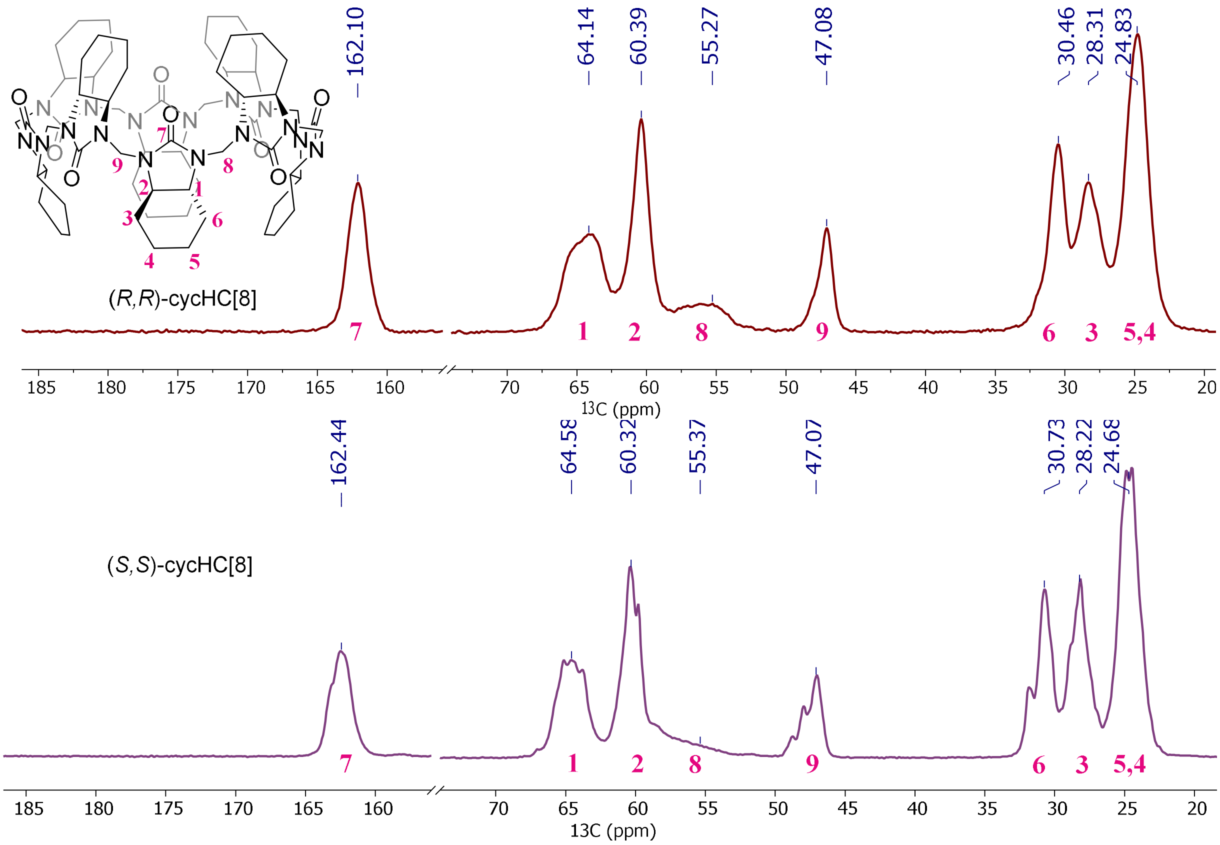

Figure S53. ^{13}C CP MAS NMR spectrum of (*R,R*)-cycHC[8] and (*S,S*)-cycHC[8], differences in ssNMR signals are caused possibly by the non-uniform distribution of crystalline and amorphous particles in two enantiomeric samples.

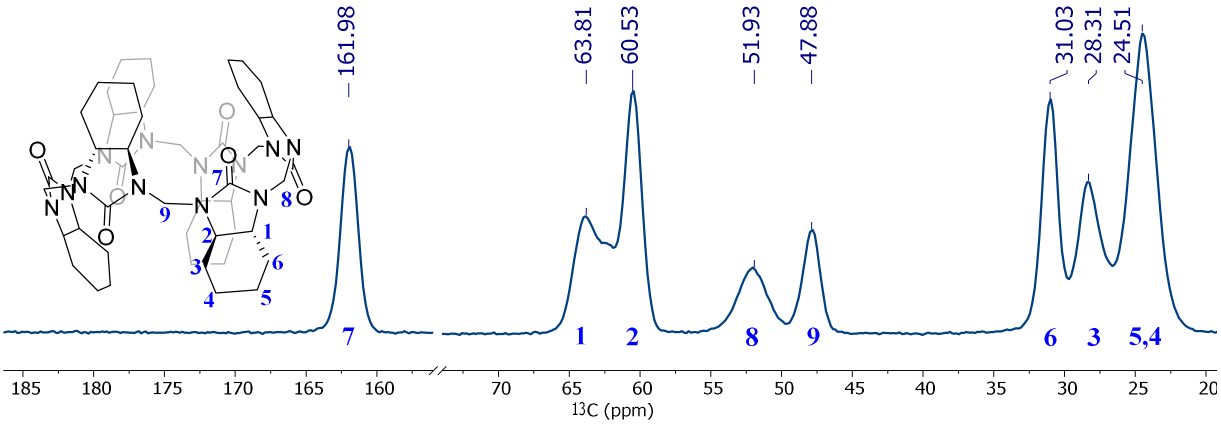

Figure S54. ^{13}C CP MAS NMR spectrum of (*R,R*)-cycHC[6].

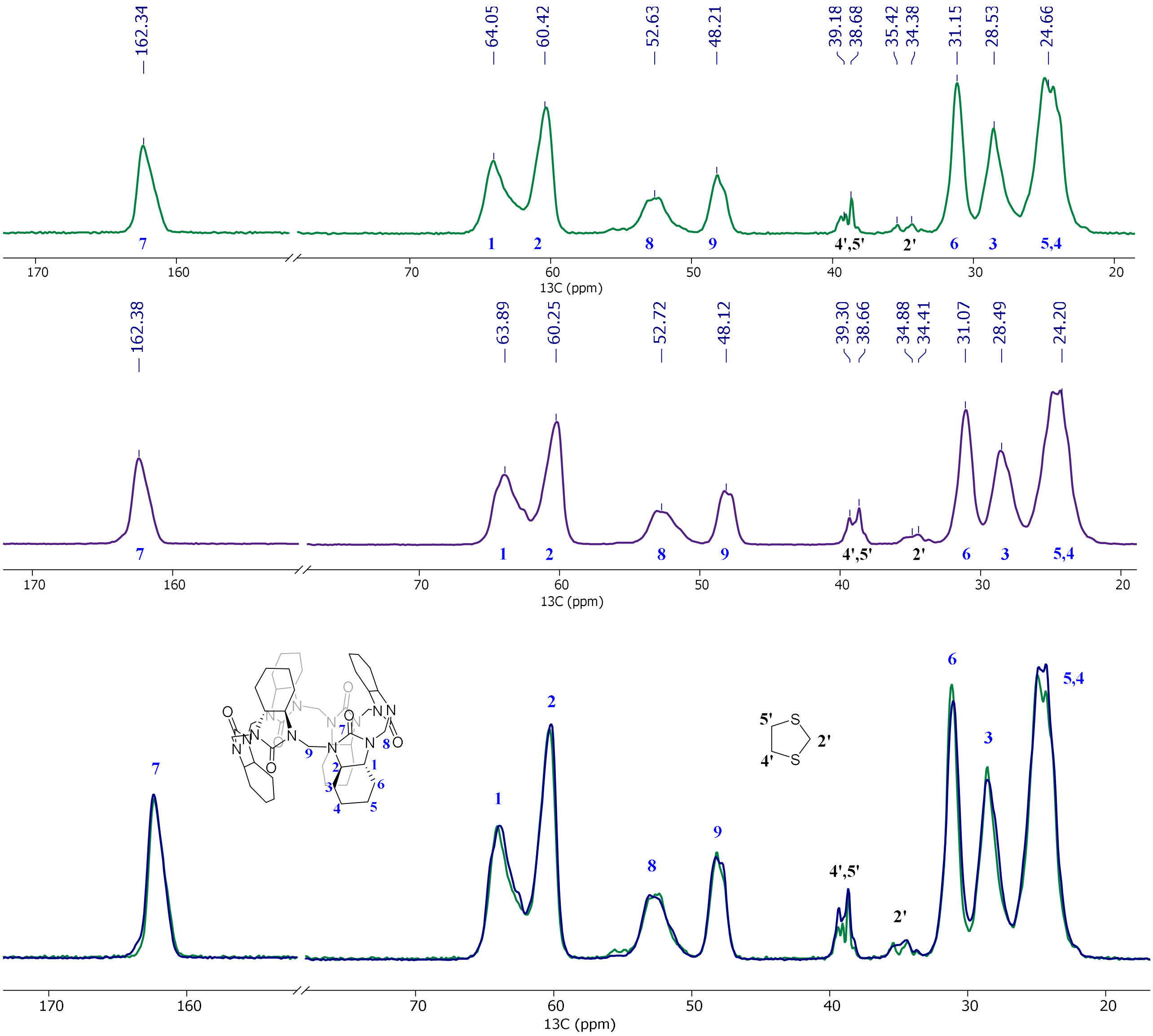

Figure S55. ^{13}C CP MAS NMR individual (upper, middle) and superimposed (lower) spectra of cycHC[6] + 1,3-dithiolane mixtures obtained without applying additional force (green) and by milling (purple). No significant changes, except slight shifts for signals 8 and 3, that correspond to the carbons located on the surface of the macrocycle, which indicates weak interaction happening outside.

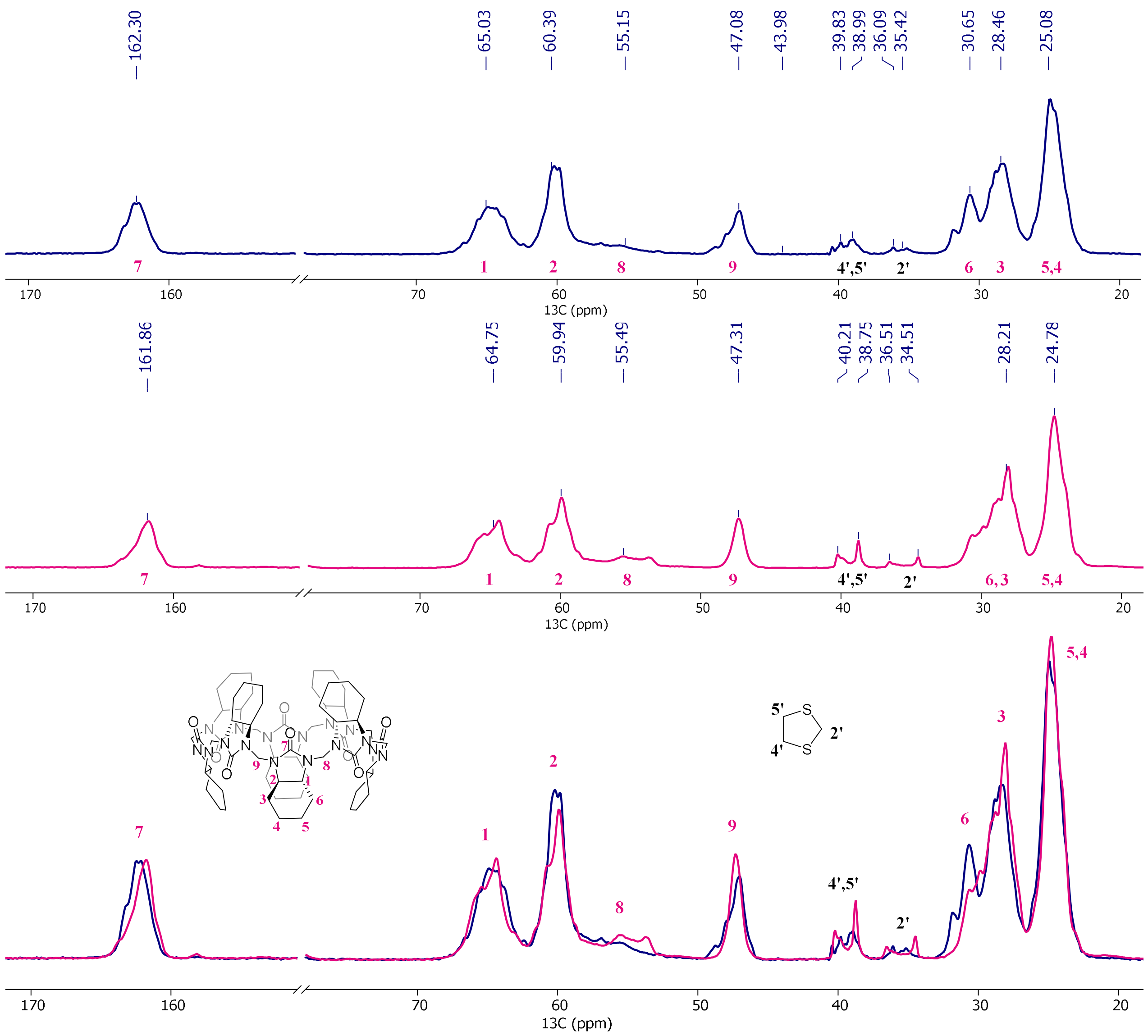

Figure S56. ^{13}C CP MAS NMR individual (upper, middle) and superimposed (lower) spectra of cycHC[8] + 1,3-dithiolane mixtures obtained without applying additional force (dark blue) and by milling (pink)

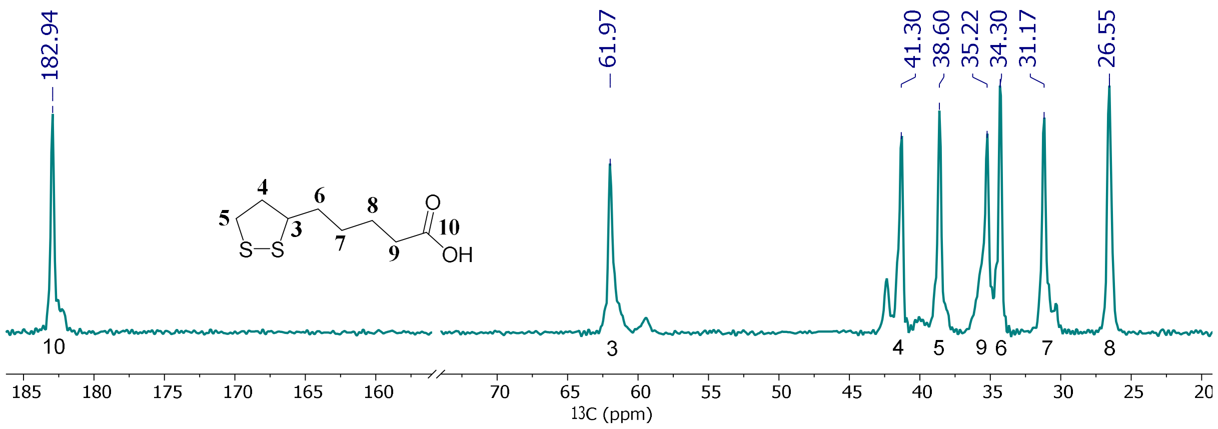

Figure S57. ^{13}C CP MAS NMR spectrum of α-lipoic acid.

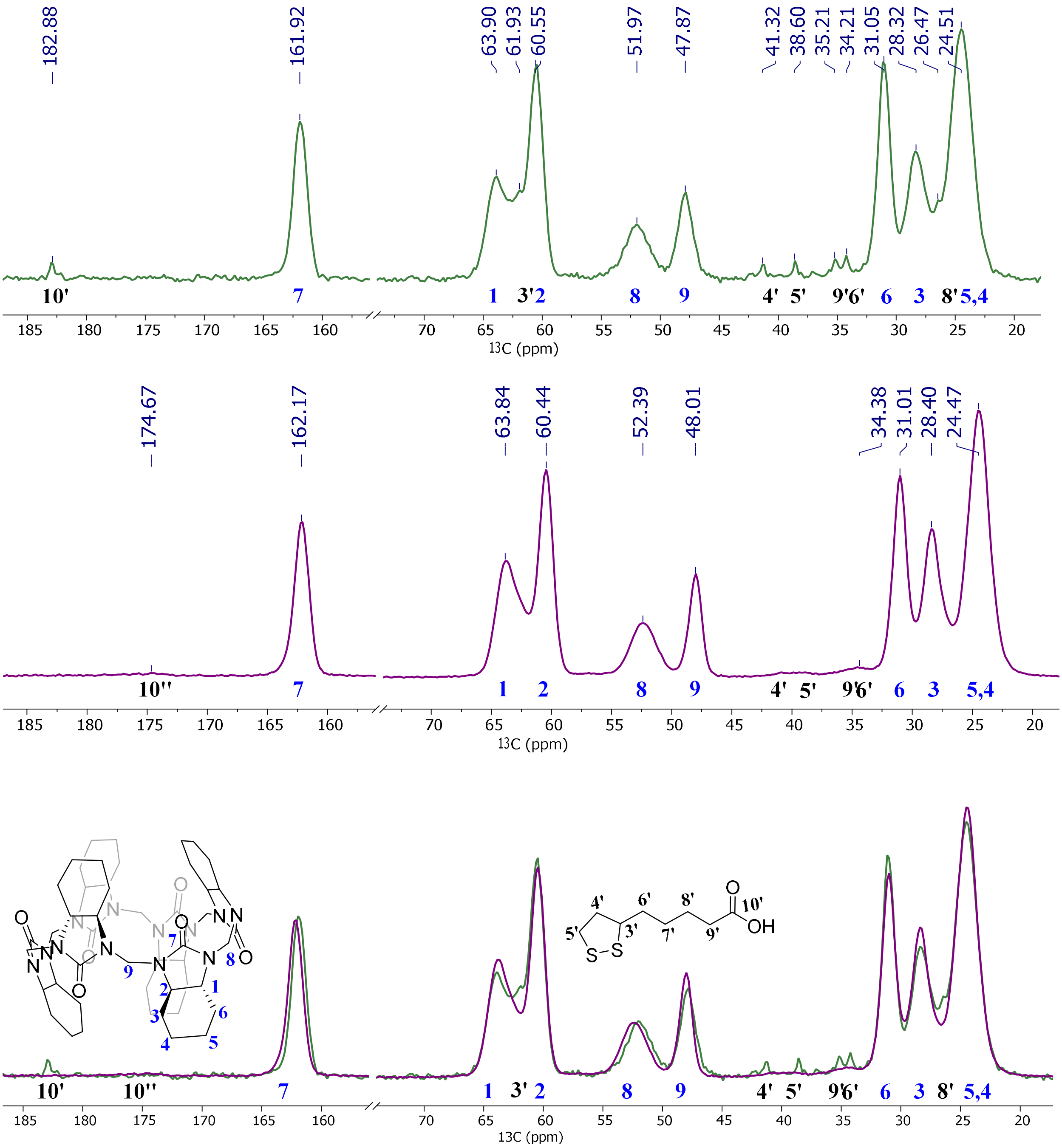

Figure S58. ^{13}C CP MAS NMR individual (upper, middle) and superimposed (lower) spectra of cycHC[6] + α-lipoic acid **9** mixtures obtained without applying additional force (green) and by milling (purple). Carboxyl group signal (10') of **9** is strongly influenced by presence of cycHC and bocomes more shealded (10'').

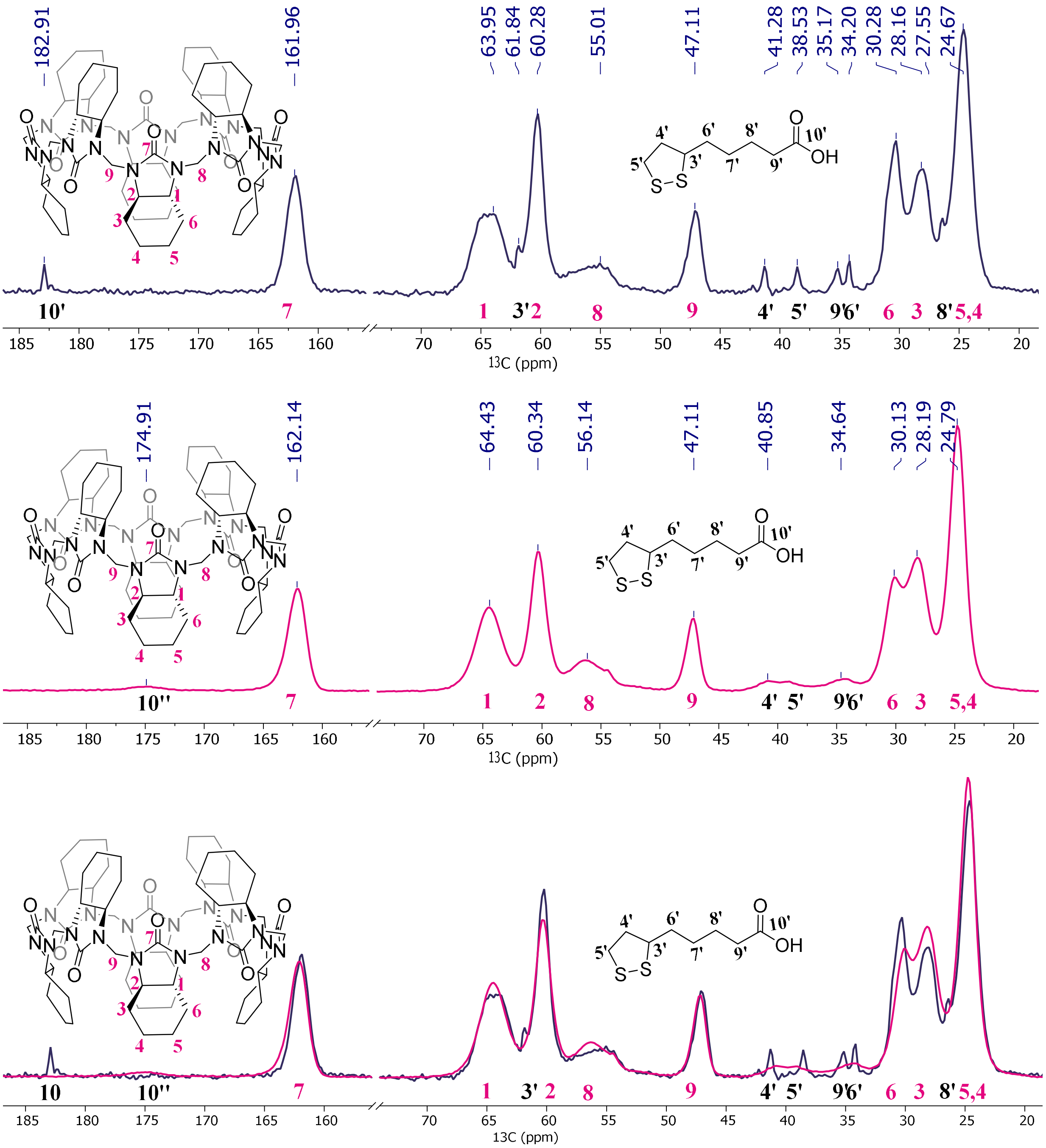

Figure S59. ^{13}C CP MAS NMR individual (upper, middle) and superimposed (lower) spectra of cycHC[8] + α-lipoic acid mixtures obtained without applying additional force (dark blue) and by milling (pink). There are noticeable changes in the cycHC[8] signal intensities, namely, C1, C8, C3, and C5 have been enhanced relative to others, which could point to decreasing conformational flexibility**.** Carboxyl group signal (10') of **9** is strongly influenced by presence of cycHC and bocomes more shealded (10'').

Upon interaction of α-lipoic acid **9** with cycHC[8] and cycHC[6] clearly visible sharp signals in the range of 32–42 ppm broaden upon milling with cycHC[*n*], suggesting a change in the close environment of **9**. Besides, the carboxylic group signal of C10' shifts from 183 ppm to 175 ppm (C10''), indicating additional shielding of the carboxyl groups, which may be caused by disaggregation of **9** and outer surface binding during physisorbtion

6. Regeneration of the sorbent

The reusability of cycHC[8] was investigated by comparing its extraction performance after four sorption–desorption cycles. The sorption step was performed analogously to the extraction procedure. Recovery procedure was not optimized and the main objective was to learn whether cycHC[8] maintains its ability to sorb the guest after washing. The desorption stage involved rinsing of 0.3–0.8 g of the material 4 times with 450 mL milli-Q water. A vaccum pressure of 25 psi was used to filter the water. After that, the filtered solid was dried for 6 hours in an oven at 110-120°C. The temperature was sufficient to eliminate the water content from the solid without destroying its morphology. The dried macrocyclic compound was then vacuum dried for additional 3 hours. The main mechanical losses of the material occured during transferring the solid from / to the centrifuge vessel and milling jar, not during filtration. The dried macrocycle was milled using the standard procedure and utilized in the subsequent cycle.

Table S21. Extraction performance of fresh and regenerated cycHC[8] in binding 1,3-dithiolane in a 5 molar excess of host

Sorption-desorption cycle	*%R*	<*%R*>[a]
**1**	79.0<br>78.9<br>78.7	**78.9±0.2**
**2**	74.4<br>74.4<br>73.3	**74.0±0.6**
**3**	70.4<br>69.5<br>67.0	**69±1.8**
**4**	69.0<br>68.9<br>68.6	**68.8±0.2**

[a] The average *%R* value is provided as mean value ± standard deviation between parallel experiments (n=3)

The structural stability of the regenerated cycHC[8] was investigated by ^{1}H NMR in $CDCl_3$ to see whether the macrocycle was affected by extraction, desorption, and milling steps. Apart from cycHC[8], no other significant signal was identified in the NMR spectrum, demonstrating that cycHC[8] is stable through all of the phases outlined and that the macrocyclic solid formed after regeneration is still of high purity (Figure S60).

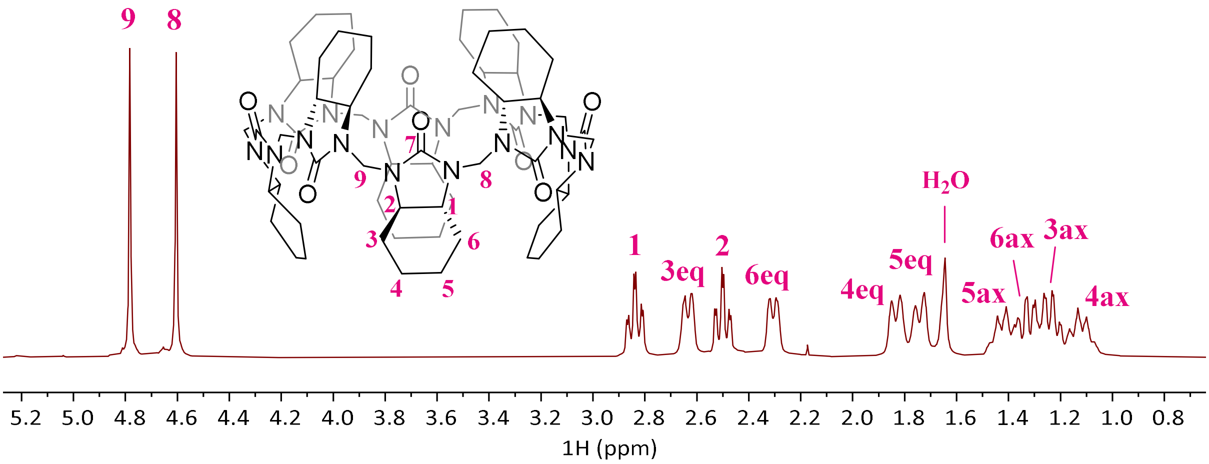

Figure S60. ^{1}H NMR spectrum of cycHC[8] in $CDCl_3$after 4 cycles of regeneration.

References

Bondi, A. (1964). van der Waals Volumes and Radii. *J. Phys. Chem.* 68, 441–451. doi:10.1021/j100785a001.

Brynn Hibbert, D., and Thordarson, P. (2016). The death of the Job plot, transparency, open science and online tools, uncertainty estimation methods and other developments in supramolecular chemistry data analysis. *Chem. Commun.* 52, 12792–12805. doi:10.1039/C6CC03888C.

Carpenter, A. E., Jones, T. R., Lamprecht, M. R., Clarke, C., Kang, I. H., Friman, O., et al. (2006). CellProfiler: image analysis software for identifying and quantifying cell phenotypes. *Genome Biol.* 7, R100–R100. doi:10.1186/gb-2006-7-10-r100.

Clark, R. C., and Reid, J. S. (1995). The analytical calculation of absorption in multifaceted crystals. *Acta Crystallogr., Sect. A: Found. Crystallogr.* 51, 887–897. doi:10.1107/S0108767395007367.

CrysAlisPro. Version 1.171.38.43 (2014). Agilent Technologies.

Dolomanov, O. V., Bourhis, L. J., Gildea, R. J., Howard, J. A. K., and Puschmann, H. (2009). OLEX2: a complete structure solution, refinement and analysis program. *J. Appl. Crystallogr.* 42, 339–341. doi:10.1107/S0021889808042726.

Kaabel, S., and Aav, R. (2018). Templating Effects in the Dynamic Chemistry of Cucurbiturils and Hemicucurbiturils. *Isr. J. Chem.* 58, 296–313. doi:10.1002/ijch.201700106.

Lees, H., Vaher, M., and Kaljurand, M. (2017). Development and comparison of HPLC and MEKC methods for the analysis of cyclic sulfur mustard degradation products. *Electrophoresis* 38, 1075–1082. doi:10.1002/elps.201600418.

Macrae, C. F., Edgington, P. R., McCabe, P., Pidcock, E., Shields, G. P., Taylor, R., et al. (2006). Mercury: visualization and analysis of crystal structures. *J. Appl. Crystallogr.* 39, 453–457. doi:10.1107/S002188980600731X.

McQuin, C., Goodman, A., Chernyshev, V., Kamentsky, L., Cimini, B. A., Karhohs, K. W., et al. (2018). CellProfiler 3.0: Next-generation image processing for biology. *PLoS Biol.* 16, e2005970–e2005970. doi:10.1371/journal.pbio.2005970.

Mecozzi, S., and Rebek, Jr., Julius (1998). The 55 % Solution: A Formula for Molecular Recognition in the Liquid State. *Chem. Eur. J.* 4, 1016–1022. doi:10.1002/(SICI)1521-3765(19980615)4:6<1016::AID-CHEM1016>3.0.CO;2-B.

Minor, W., Cymborowski, M., Otwinowski, Z., and Chruszcz, M. (2006). HKL-3000: the integration of data reduction and structure solution - from diffraction images to an initial model in minutes. *Acta Crystallogr., Sect. D: Biol. Crystallogr.* 62, 859–866. doi:10.1107/S0907444906019949.

Persistence of Vision Raytracer. Version 3.7 (2004). Persistence of Vision Pty. Ltd. Available at: http://www.povray.org/download/.

Prigorchenko, E., Öeren, M., Kaabel, S., Fomitšenko, M., Reile, I., Järving, I., et al. (2015). Template-controlled synthesis of chiral cyclohexylhemicucurbit[8]uril. *Chem. Commun.* 51, 10921–10924. doi:10.1039/c5cc04101e.

PubChem [Internet]. Bethesda (MD): National Library of Medicine (US), National Center for Biotechnology Information; 2004-. Available at: https://pubchem.ncbi.nlm.nih.gov/.

Rowland, R. S., and Taylor, R. (1996). Intermolecular Nonbonded Contact Distances in Organic Crystal Structures: Comparison with Distances Expected from van der Waals Radii. *J. Phys. Chem.* 100, 7384–7391. doi:10.1021/jp953141+.

Schmidtchen, F. P. (2006). "Isothermal Titration Calorimetry in Supramolecular Chemistry," in *Analytical Methods in Supramolecular Chemistry* (John Wiley & Sons, Ltd), 55–78. doi:https://doi.org/10.1002/9783527610273.ch3.

Sheldrick, G. M. (2015a). Crystal structure refinement with SHELXL. *Acta Crystallogr., Sect. C: Cryst. Struct. Commun.* 71, 3–8. doi:10.1107/S2053229614024218.

Sheldrick, G. M. (2015b). SHELXT - Integrated space-group and crystal-structure determination. *Acta Crystallogr., Sect. A: Found. Crystallogr.* 71, 3–8. doi:10.1107/S2053273314026370.

Thordarson, P. (2011). Determining association constants from titration experiments in supramolecular chemistry. *Chem. Soc. Rev.* 40, 1305–1323. doi:10.1039/C0CS00062K.

Turnbull, W. B., and Daranas, A. H. (2003). On the Value of c: Can Low Affinity Systems Be Studied by Isothermal Titration Calorimetry? *J. Am. Chem. Soc.* 125, 14859–14866. doi:10.1021/ja036166s.
